# Impacts of digital third-wave interventions for adults on wellbeing and mental health outcomes: A systematic review and meta-analysis of randomized controlled trials

**DOI:** 10.1371/journal.pdig.0001764

**Published:** 2026-10-01

**Authors:** Tom C. Gordon, Andrew H. Kemp, Josh Hope-Bell, Darren J. Edwards

**Affiliations:** 1 Department of Public Health, Swansea University, Swansea, United Kingdom; 2 School of Psychology, Swansea University, Swansea, United Kingdom; 3 Division of Psychological Medicine and Clinical Neurosciences, Cardiff University, Cardiff, United Kingdom; Universidade Federal de Sao Paulo, BRAZIL

## Abstract

To synthesise the efficacy of digital third-wave process-based interventions (e.g., ACT, Mindfulness, Compassion) for improving wellbeing and ameliorating mental health symptoms in adults. A prospectively registered systematic review (OSF: 10.17605/OSF.IO/5RMU2) and random-effects meta-analysis of randomised controlled trials (RCTs) was conducted across six databases through January 2026. Certainty of evidence was assessed using the GRADE framework. Interventions (*k* = 147; *N* = 26,391) yielded small-to-moderate immediate post-intervention improvements in wellbeing (*k* = 46, *g* = 0.36), depression (*k* = 110, *g* = 0.44), anxiety (*k* = 93, *g* = 0.40), stress (*k* = 66, *g* = 0.46), quality of life (*k* = 32, *g* = 0.30), and psychological flexibility (*k* = 51, *g* = 0.41). Heterogeneity was substantial across most outcomes (*I2* > 75%). Benefits were observed across healthy (61 trials; *n* = 15,770), mental health (41 trials; *n* = 6,130), and medical samples (46 trials; *n* = 4,491), with population type not significantly moderating immediate post-intervention effects. Comparator type significantly moderated several analyses, with smaller effects against active than inactive controls. Guidance did not significantly moderate immediate post-intervention effects, although it predicted larger short-term depression effects (*QM* = 4.17, *p* = .048). At short-term follow-up, effects remained statistically supported across all outcomes, while only depression, anxiety, and stress remained statistically supported at medium-term follow-up. GRADE certainty was moderate for most immediate post-intervention outcomes and low for quality of life. Digital third-wave interventions produced small-to-moderate average benefits across symptom-focused, wellbeing-focused, and process outcomes. Effects were smaller against active comparators, indicating that pooled benefits should be interpreted in relation to comparator intensity. Future research should prioritise rigorous active controls, longer follow-up, and direct tests of whether changes in psychological flexibility mediate improvements in symptoms and wellbeing.

## Author summary

Digital programmes based on acceptance, mindfulness, compassion, and related approaches are increasingly used to support adult mental health, but their overall benefits and durability remain uncertain. We brought together evidence from 147 randomised controlled trials involving 26,391 participants. We found that these programmes produced small-to-moderate average improvements in wellbeing, depression, anxiety, stress, quality of life, and people’s ability to respond flexibly to difficult thoughts and emotions immediately after the intervention. Benefits were observed among healthy adults and people with mental or physical health conditions. However, effects were generally smaller when programmes were compared with active alternatives, such as structured psychological support, rather than inactive controls such as waiting lists. Improvements in depression, anxiety, and stress were maintained over the short term, but longer-term evidence was limited and less consistent. Results also varied substantially between studies, and most trials had some methodological concerns, meaning that the precise size of benefit remains uncertain. Our findings suggest that these digital programmes can provide useful mental health support, but future trials should compare them with credible active treatments and establish how benefits can be maintained over time.

## Introduction

Common mental disorders (CMDs), largely comprising depressive and anxiety disorders, remain the leading contributors to global disability. In 2019, mental disorders accounted for 125.3 million years lived with disability globally, with depressive and anxiety disorders representing the largest contributors to mental-disorder burden, accounting for 37.3% and 22.9% mental-disorder disability-adjusted life years (DALYs) respectively [1]. Lifetime prevalence estimates for major depression are substantial, with prospective studies suggesting rates exceeding 30% and cross-national retrospective surveys estimating 14.6% in high-income countries and 11.1% in low- to middle-income countries, alongside an approximately 2:1 female-to-male prevalence ratio [2,3]. Depression also follows a variable and often recurrent course, with 70–90% of patients with major depressive disorder (MDD) in population-based samples recovering within one year, but around 80% of remitted patients experiencing at least one recurrence during their lifetime [4]. Anxiety disorders, including panic disorder, generalised anxiety disorder, social anxiety disorder, and specific phobias, are similarly prevalent, affecting up to one third of adults and commonly co-occurring with depression, compounding functional impairment and healthcare burden [5]. Beyond diagnosable CMDs, a substantial proportion of adults experience subthreshold psychological distress that still carries meaningful impairment and risk of progression [6,7]. Although digitally delivered third-wave interventions are increasingly used to address this broader spectrum of distress at scale, evidence is heterogeneous and fragmented across outcomes, populations, and intervention families, and existing reviews have not synthesised these approaches as a unified process-based class. Accordingly, the present systematic review and meta-analysis integrates RCT evidence on digital third-wave, process-based interventions in adults to clarify their effects on prespecified mental health outcomes and to examine key sources of variability in effects.

Third-wave, process-based therapies conceptualise distress through the construct of psychological flexibility. Psychological flexibility refers to the capacity to remain in contact with present-moment experiences, including distressing thoughts and emotions, while pursuing meaningful action guided by personal values [8–10]. Low psychological flexibility is characterised by patterns such as experiential avoidance, cognitive fusion, and behavioural rigidity that undermine valued action, which can maintain or exacerbate symptoms over time [11]. Across observational evidence, psychological flexibility shows robust associations with both reduced illbeing and enhanced wellbeing, supporting its value as a transdiagnostic, modifiable target for mental health promotion [12,13].

Experimental and intervention evidence also indicates that targeting psychological flexibility can yield meaningful improvements across both symptom reduction and positive functioning. For example, a multisite RCT of ACT with university students found greater psychological flexibility, wellbeing, and school engagement, alongside lower stress, anxiety, and depressive symptoms, compared with wait-list control [14]. This pattern aligns with the core claim of process-based approaches, that training mindfulness, acceptance, cognitive defusion, and values-oriented behaviour can simultaneously reduce distress and strengthen wellbeing, rather than focusing narrowly on symptom elimination [8,9].

Digital delivery has the potential to extend these approaches at scale. Digital third-wave interventions deliver therapeutic content through websites, smartphone applications, chatbots, videoconferencing, and increasingly immersive platforms such as virtual reality. Programmes commonly include brief multimedia lessons, guided practices (e.*g*., audio mindfulness exercises), interactive worksheets, and ecological prompts that encourage rehearsal in daily life, with guidance ranging from fully automated formats to therapist-supported messaging or blended care [15]. These delivery options are particularly relevant because traditional one-to-one psychological services are unlikely to meet population-level need, and access to psychological treatment is often constrained by barriers including availability, cost, time, transport, and stigma [16,17]. Internet-based interventions may therefore improve access to evidence-based psychological support while reducing reliance on face-to-face therapist time, although their role alongside traditional services depends on effective implementation and dissemination [18].

Evidence to date suggests that digital third-wave interventions are promising, but effects are heterogeneous and the literature remains fragmented across outcomes, populations, and intervention families. Prior meta-analyses of digital interventions delivered via apps report small benefits for depression and anxiety, with substantial heterogeneity [19,20]. Online mindfulness-based interventions have similarly shown modest improvements across mental health outcomes [21]. Syntheses focusing on digitally delivered ACT indicate improvements in psychological flexibility and mental health symptoms, although effects appear to vary by outcome, comparator type, and delivery format [22,23]. In parallel, broader reviews of third-wave therapies for depression suggest potential comparability with treatment-as-usual, yet conclusions have often been limited by study quality, diversity of interventions, and imprecision [24]. Taken together, these findings support the plausibility of benefit while underscoring uncertainty about the size and consistency of effects across populations, outcomes, and intervention families.

A further limitation of existing evidence is that reviews have frequently focused on single modalities (e.*g*., mindfulness apps) or single outcome classes (e.*g*., depressive symptoms), rather than synthesising digital third-wave interventions as a process-based class. This matters because contemporary intervention science increasingly emphasises mechanisms and processes that cut across diagnostic categories and delivery formats [8,25]. In addition, intervention effects may plausibly differ by participant population (healthy, mental health condition, medical condition), the degree of human support, intervention family (e.*g*., ACT vs mindfulness-based), comparator type (active vs inactive), and programme characteristics such as duration and intensity. Comparator type may also shape observed effects, as control condition design can influence effect-size estimates in psychological intervention trials, with wait-list controls tending to produce larger effects than more structured or active comparators [26,27]. Such potential moderators have rarely been examined together in a unified framework.

Against this background, the present systematic review and meta-analysis synthesises RCTs of digitally delivered third-wave, process-based psychological interventions in adults, compared with both active and inactive control conditions. The primary objective is to estimate pooled effects on two co-primary outcomes, subjective wellbeing and depressive symptom severity. Secondary objectives are to estimate pooled effects on anxiety, perceived stress, psychological flexibility, and quality of life (QoL). For all outcomes, we examine effects immediately post-intervention and, where data permit, at prespecified follow-up windows (short-term ≤3 months; medium-term >3–6 months; long-term >6 months). To inform interpretation and implementation, we will explore whether effect sizes vary according to prespecified sources of heterogeneity, including population group (healthy, mental health condition, medical condition), intervention modality, therapist involvement (guided vs unguided), and comparator type (active vs inactive), using subgroup analyses and, where sufficient studies contribute, meta-regression. By integrating outcomes reflecting both reductions in distress and improvements in wellbeing, alongside process measures, the review aims to provide a comprehensive, decision-relevant summary of the likely benefits and contextual moderators of digital third-wave interventions, to inform clinical practice, commissioning, and future intervention development [28].

## Methods

### Reporting standards and protocol

This systematic review and meta-analysis has been reported according to the Preferred Reporting Items for Systematic Reviews and Meta-Analyses (PRISMA) [29]. The protocol was prospectively registered on the Open Science Framework (OSF; https://doi.org/10.17605/OSF.IO/5RMU2), specifying eligibility criteria, outcomes, time windows, measurement hierarchy, and planned analyses. The completed PRISMA checklist is provided as S1 Checklist in S1 Appendix.

### Eligibility criteria

The eligibility was determined using the Population Intervention Comparison Outcome Study-type (PICOS) framework [30]. This review included RCTs exploring the effectiveness of digital third-wave interventions for improving mental health outcomes in adults. Detailed inclusion and exclusion criteria are described in Table 1.

**Table 1:** The eligibility criteria used for decision-making about the inclusion of studies in this systematic review and meta-analysis.

Population	Included populations consisted of adults (18+) who were either: (1) individuals without a diagnosed mental health condition at the time of study enrolment, as reported by the study authors, or (2) individuals with an official clinical diagnosis (by an accredited mental health practitioner) of ongoing anxiety, stress, or depression, or where studies identified and reported existing anxiety, stress, or depression using mental health psychometric measures. This inclusion criterion was kept open without requiring specific psychometric cut-off scores, recognising variability in standardisation across mental health measures. Populations were excluded if their condition or treatments could confound the outcomes. This included populations with neurological conditions such as Alzheimer’s disease, dementia, or brain injury, as well as those who were pregnant.
**Intervention**	Studies evaluating digitally delivered third-wave, process-based psychological interventions were included. Eligible interventions explicitly targeted one or more core third-wave processes, including mindfulness, acceptance, cognitive defusion or decentring, values clarification and committed action, self-compassion, or related contextual behavioural processes. Recognised third-wave approaches included Acceptance and Commitment Therapy (ACT), Dialectical Behaviour Therapy (DBT), Mindfulness-Based Cognitive Therapy (MBCT), Mindfulness-Based Stress Reduction (MBSR), Compassion-Focused Therapy (CFT), third-wave behavioural activation, and other eligible process-based programmes. Positive psychology protocols were eligible where they explicitly incorporated one or more of these third-wave processes. Studies focusing solely on traditional or non-digital formats of these therapies were excluded. Interventions that did not primarily target third-wave principles, such as psychoeducation-only apps or general mental health support platforms, were not included. Interventions that combined third-wave approaches with pharmacological treatments as a central component of the study design were excluded.
**Comparison**	All eligible RCTs evaluating digital third-wave interventions were included regardless of the type of control condition. Both active controls (for example, alternative psychotherapeutic or non-specific digital interventions) and inactive controls (for example waitlist, sham interventions, or no intervention) were accepted. In the primary analyses, data from studies using both active and inactive comparators were pooled.
**Outcome**	We extracted psychometric outcomes prespecified in the review protocol, focusing on: (1) subjective wellbeing, (2) depressive symptoms, (3) anxiety symptoms, (4) perceived stress/psychological distress, (5) psychological flexibility/inflexibility, and (6) QoL. Specific outcome measures/questionnaires extracted for each construct are reported in Text A in S4 Appendix. The primary timepoint was the immediate post-intervention assessment. Follow-up outcomes were extracted and categorised as short-term (≤3 months), medium-term (>3–6 months), and long-term (>6 months) when sufficient data were available for synthesis, using prespecified time windows to support comparability across trials.
**Study-type**	RCTs were eligible (including studies described as pilot or feasibility trials) provided they evaluated an eligible digital third-wave intervention versus an eligible comparator and reported at least one eligible outcome. Parallel-group, cluster-randomised, and crossover RCTs were eligible, with cluster and crossover designs handled using Cochrane-recommended approaches to unit-of-analysis issues. Studies that were not randomised were excluded. Additional criteria included the availability of full-text papers. No restrictions were placed on publication status, language, or length of follow-up; non-English full texts were translated where feasible, and reports that could not be translated were listed as studies awaiting classification. Published data were used where possible; authors were contacted where feasible when data were reported as collected but not in an extractable form.

### Search strategy and selection process

A systematic search was conducted across PubMed/MEDLINE, PsycINFO, PsycArticles, the Cochrane Library (CENTRAL), Scopus, and EMBASE from database inception to January 2026. No restrictions were placed on publication status. For the search, a combination of MeSH terms and keywords were used. The “title and abstract fields only” and “all fields” filters were used (see Text A in S2 Appendix).

Initial screening and full-text review were conducted independently in duplicate by two blinded reviewers (TCG and JH-B). Discrepancies were resolved through discussion. Non-English full texts were translated where feasible. Authors were contacted (two attempts, two weeks apart) for unavailable full texts or unextractable data. Studies were excluded only if eligibility could not be confirmed after these attempts.

### Data extraction and quality assessment

Data extraction was conducted in duplicate using a standardised form developed for this review (see Table A in S3 Appendix). The extraction template was informed by JBI guidance but adapted to capture specific intervention characteristics (e.*g*., third-wave modality, guidance intensity) and outcome data required for the planned meta-analyses. Extracted variables included citation details, population characteristics, intervention specifics, and psychometric outcome data.

Risk of bias was assessed independently by two reviewers (TCG and JH-B) using the Cochrane Risk of Bias 2 (RoB 2) tool [31]. Assessments were made at the result level for each outcome covering five domains: randomisation process, deviations from intended interventions, missing outcome data, outcome measurement, and selection of the reported result (see Figs A–D in S5 Appendix). Discrepancies were resolved through consultation with a third reviewer (DJE). Visualisations were generated using ‘robvis’ [32]. Risk-of-bias judgements were incorporated via sensitivity analyses excluding results at overall high risk of bias.

### Meta-analysis

Meta-analyses were conducted using Comprehensive Meta-Analysis software [33]. Pooled effect estimates were calculated when at least two studies provided data for the same outcome construct. To avoid unit-of-analysis errors, each study contributed at most one effect size per outcome at each time point selected using a prespecified hierarchy (see Text A in S4 Appendix). Immediate post-intervention means, standard deviations, and sample sizes were extracted to calculate standardised mean differences (SMDs) using Hedges’ *g* to allow for the synthesis of continuous outcomes across different scales [34–36]. All outcomes were oriented so that positive values indicated improvement. To facilitate high-resolution reporting, forest plots and funnel plots were created using the Metafor package [37] in R [38]. For immediate post-intervention analyses with at least ten effect-size estimates, contour-enhanced funnel plots were visually inspected to assess possible small-study effects and publication bias, consistent with protocol and guidance that funnel-plot methods have limited power when fewer than ten studies contribute [39,40]. Formal regression tests for funnel-plot asymmetry were not used because effect sizes expressed as standardised mean differences are naturally correlated with their standard errors, which can distort asymmetry tests [40,41]. Interpretation also considered heterogeneity, influence diagnostics, and fixed-effect versus random-effects sensitivity analyses.

Random-effects models were used to account for clinical and methodological heterogeneity. Within CMA, random-effects models used DerSimonian-Laird between-study variance estimation, with 95% confidence intervals calculated using the normal approximation. Summary effects were reported with 95% confidence intervals (CI) and, where feasible, 95% prediction intervals (PI) [42]. The primary analysis focused on immediate post-intervention outcomes, defined as assessments conducted at programme completion, pooling all eligible comparators. Follow-up outcomes were synthesised using prespecified windows [43] defined as short-term (≤3 months), medium-term (>3–6 months), and long-term (>6 months). Timepoints with fewer than two comparable effect-size estimates for a given outcome were not meta-analysed.

### Assessment of heterogeneity and moderators

Statistical heterogeneity was assessed using the *I²* statistic, which describes the proportion of total variability in observed effect estimates attributable to between-study heterogeneity rather than sampling error. *I²* values were interpreted using Cochrane’s overlapping thresholds: values of 0% to 40% suggest low heterogeneity, 30% to 60% represent moderate heterogeneity, 50% to 90% represent substantial heterogeneity, and 75% to 100% indicate considerable heterogeneity [42].

Prespecified subgroup analyses were conducted to examine whether intervention effects differed by population group (healthy, mental health condition, medical condition), intervention type (ACT, DBT-informed, MBCT, MBSR, compassion-focused therapy, and other eligible third-wave programmes), comparator type (active versus inactive), and guidance (guided versus unguided). Between-subgroup differences were evaluated using between-group *Q* statistics to test whether pooled effects differed across subgroup levels [42]. In addition, categorical moderators were formally tested using mixed-effects meta-regression models, with moderation assessed using the omnibus QM statistic, which evaluates whether inclusion of a moderator explains a significant proportion of between-study heterogeneity [42]. Consistent with Cochrane guidance and standard recommendations for regression modelling in meta-analysis [42,44], formal mixed-effects meta-regression was undertaken only when at least ten effect-size estimates were available for a given outcome, timepoint, and moderator. This threshold referred to the full moderator analysis, not to each individual subgroup level. Subgroup estimates with smaller level-specific *k* were retained for descriptive completeness but interpreted as exploratory.

### Sensitivity analyses and certainty of evidence

Sensitivity analyses were conducted to test the robustness of the primary results. As a robustness check, change-from-baseline effects were synthesised separately to account for differences in standardisation metrics between change scores and final values. Where standard deviations of change were not reported, they were imputed assuming baseline–post correlations of 0.3, 0.5, and 0.7. We further assessed robustness by comparing random-effects models with fixed-effect models to examine whether the choice of model influenced statistical significance, and by repeating meta-analyses after excluding results judged at overall high risk of bias. Finally, for main analyses with substantial heterogeneity (*I*^*2*^ ≥ 75%) and at least ten contributing studies, influence diagnostics were used to examine whether any single study had a disproportionate impact on the pooled effect estimate or on measures of heterogeneity [45].

The certainty of evidence for immediate post-intervention outcomes was assessed using the GRADE framework, evaluating risk of bias, inconsistency, indirectness, imprecision, and publication bias. Imprecision was assessed using a smallest important effect threshold of 0.20 standardised mean difference [46]. Confidence intervals extending below this threshold were treated as serious imprecision.

## Results

### Study selection

The database search identified 9,087 records. After removal of duplicates (*n* = 2,234), 6,853 titles and abstracts were screened. A total of 189 full-text reports were assessed for eligibility, of which 42 were excluded for prespecified reasons (see Fig 1). In total, 147 RCTs were included, contributing data to at least one meta-analysis. A complete list of included studies is provided in Text A in S6 Appendix.

**Fig 1:**
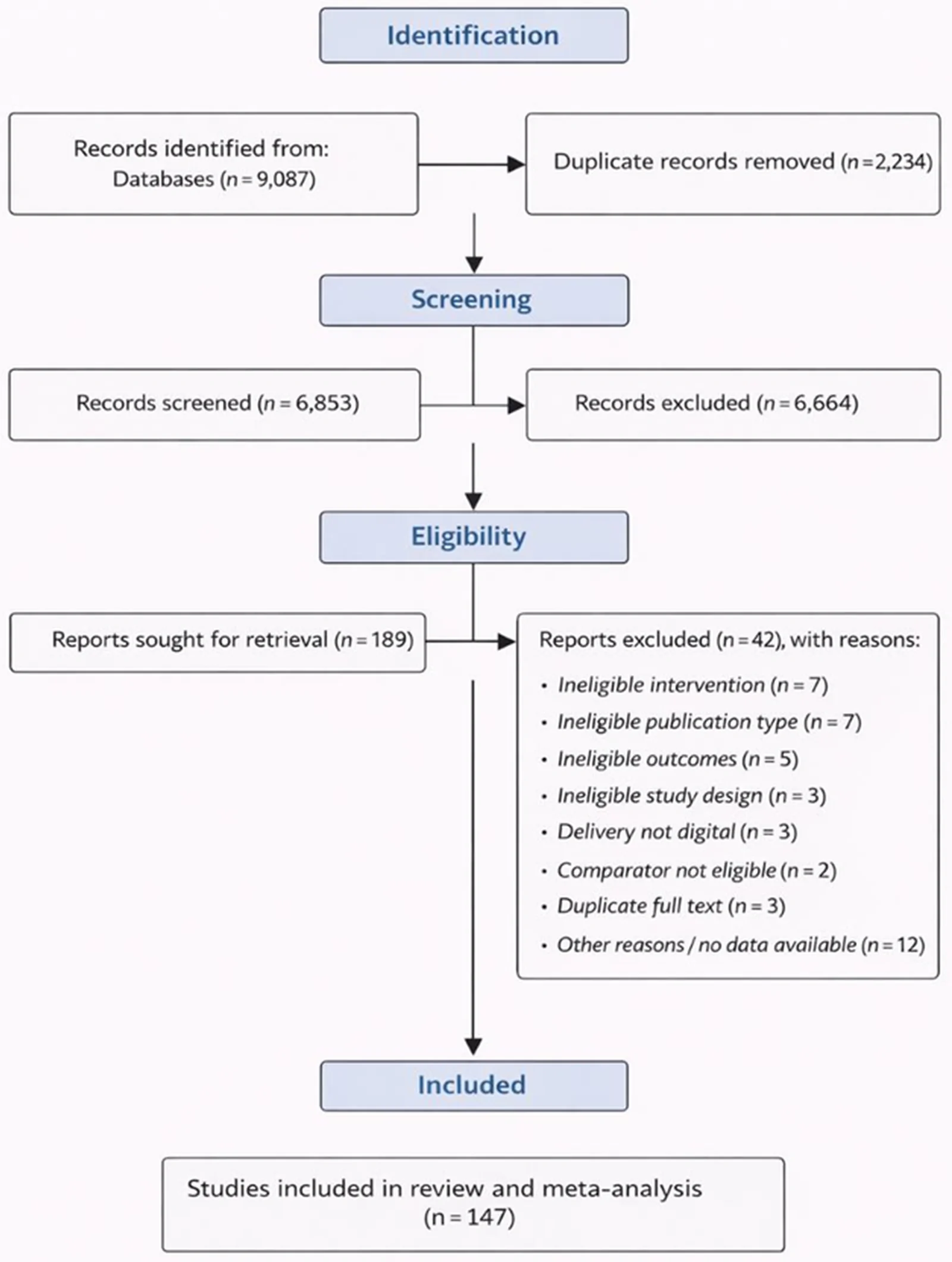
PRISMA flow diagram.

### Study characteristics

Characteristics of included studies are summarised in Table 2. The extraction dataset comprised 147 RCTs with an estimated 26,391 participants (median trial size 99, IQR 74.5 to 150.0; range 30–4,411). Population categories were not mutually exclusive because one trial included both a healthy caregiver cohort and a medical condition cohort [47]. Trials recruited healthy participants (61 trials; *n* = 15,770), participants with a mental health condition (41 trials; *n* = 6,130), and participants with a medical condition (46 trials; *n* = 4,491).

**Table 2:** Characteristics of included studies (population, intervention type, delivery format, comparator, outcomes, timepoints).

Characteristic	Value
Included trials (*k*)	147
Total participants (*N*)	26,391
Sample size per trial	Median 99 (IQR 74.5–150.0); range 30–4,411
**Population type**	
Healthy	61 (41.5%)
Mental health condition	41 (27.9%)
Medical condition	46 (31.3%)
**Intervention family (intervention arms)**	
ACT-based	64 (43.5%)
Mindfulness-based interventions (MBI)	46 (31.3%)
Mindfulness-Based Cognitive Therapy (MBCT)	9 (6.1%)
Mindfulness-Based Stress Reduction (MBSR)	11 (7.5%)
Mindfulness-integrative variants	8 (5.4%)
DBT-informed	5 (3.4%)
CFT	4 (2.7%)
**Comparator type (by study)**	
Inactive control only	97 (66.0%)
Active control only	26 (17.7%)
Active and inactive controls	24 (16.3%)
**Therapist involvement (intervention arms)**	
Guided	83 (56.5%)
Unguided	60 (40.8%)
Mixed (guided + unguided)	4 (2.7%)
**Outcomes assessed (immediate post-intervention)**	
Wellbeing	46 (31.3%)
Depression	110 (74.8%)
Anxiety	93 (63.3%)
Stress	66 (44.9%)
Psychological flexibility	51 (34.7%)
Quality of life	32 (21.8%)
**Follow-up assessments available**	
Short-term (≤3 months)	62 (42.2%)
Medium-term (>3–6 months)	27 (18.4%)
Long-term (>6 months)	12 (8.2%)

Note. Counts are number of trials (k) unless otherwise stated. Population categories are not mutually exclusive because one RCT included both healthy caregiver and medical condition cohorts.

At the trial level, interventions were most commonly ACT-based programmes (64 trials) and mindfulness-based programmes (74 trials; including mindfulness-based interventions, MBSR, MBCT, and mindfulness-integrative variants), with smaller numbers evaluating DBT-informed (5 trials) and compassion-focused (4 trials) interventions. Most trials compared the intervention against inactive controls only (97 trials), while 26 used active controls only and 24 included both active and inactive comparators.

Interventions were delivered as guided programmes only (83 trials), unguided programmes only (60 trials), or both guided and unguided variants within the same trial (4 trials). Programme duration was generally brief (reported in 131 trials; median 7 weeks, IQR 4–8; range 0.7 to 24 weeks), with intervention frequency and intensity varying substantially across programmes. All trials reported immediate post-intervention outcomes; follow-up assessments were available for 62 trials at short-term, 27 at medium-term, and 12 at long-term timepoints. Immediately post-intervention, sufficient studies contributed to meta-analysis for wellbeing (*k* = 46), depression (*k* = 110), anxiety (*k* = 93), stress (*k* = 66), psychological flexibility (*k* = 51), and QoL (*k* = 32).

### Risk of bias in included studies

Risk of bias visualisations are presented in Figs A–D in S5 Appendix, with study-level judgements available on OSF. Across studies, the most common sources of bias were concerns arising from the randomisation process, deviations from intended interventions, and missing outcome data. 3 studies were rated overall ‘low risk’, 120 as ‘some concerns’, and 24 as ‘high risk’. Risk-of-bias judgements were incorporated via sensitivity analyses that repeated meta-analyses after excluding results judged at overall high risk of bias.

### Primary outcome measures

#### Wellbeing.

Immediately post-intervention, digital third-wave interventions were associated with moderate improvements in wellbeing (*k* = 46, *g* = 0.36, 95% CI [0.24, 0.49], *p* < .001). Heterogeneity was substantial (*I²* = 82%, PI [−0.34, 1.07]), influenced primarily by a single outlier trial evaluating Mindful Self-Compassion [48] (*g* > 5.0). Sensitivity analyses confirmed that this study disproportionately drove between-study variance. Its exclusion reduced heterogeneity from 82% to 60% and attenuated the pooled estimate to *g* = 0.30 (95% CI [0.22, 0.39]), thou*g*h the effect remained statistically significant. Regarding comparator type, effects were markedly larger relative to inactive controls (*k* = 38, *g* = 0.44, 95% CI [0.27, 0.60], *p* < .001) but were si*g*nificantly attenuated when com*p*ared against active controls (*k* = 12, *g* = 0.15, 95% CI [0.06, 0.24], *p* = .002).

Benefits were observed transdiagnostically, with significant improvements found in Healthy (*k* = 19, *g* = 0.32, 95% CI [0.19, 0.46], *p* < .001), mental health condition (*k* = 12, *g* = 0.36, 95% CI [0.19, 0.54], *p* < .001), and medical condition samples (*k* = 15, *g* = 0.53, 95% CI [0.13, 0.93], *p* = .010). Effects were evident in both guided interventions (*k* = 25, *g* = 0.49, 95% CI [0.27, 0.72], *p* < .001) and unguided formats (*k* = 21, *g* = 0.28, 95% CI [0.16, 0.41], *p* < .001), although guidance was not supported as a significant immediate post-intervention moderator in formal meta-regression. Across intervention families, consistent benefits were observed for ACT (*k* = 20, *g* = 0.34, 95% CI [0.23, 0.45], *p* < .001), MBCT (*k* = 5, *g* = 0.21, 95% CI [0.12, 0.31], *p* < .001), and broader mindfulness-based interventions (*k* = 10, *g* = 0.26, 95% CI [0.05, 0.46], *p* = .015).

At short-term follow-up (≤3 months), effects were smaller but remained significant overall (*k* = 17, *g* = 0.18, 95% CI [0.05, 0.31], *p* = .006; *I²* = 66%). Interventions showed a statistically reliable effect when compared with inactive controls (*g* = 0.33, *p* = .002), but not when compared with active controls (*g* = 0.02, *p* = .615). Benefits were also observed in mental health samples (*g* = 0.28, *p* = .021). Guided interventions showed a statistically reliable effect (*g* = 0.38, *p* = .012). The unguided subgroup estimate was smaller and not statistically reliable (*g* = 0.09, *p* = .168). However, guidance was not a significant short-term moderator. By medium-term follow-up, the overall pooled effect was no longer statistically distinguishable from zero (*k* = 7, *g* = 0.12, 95% CI [−0.06, 0.30], *p* = .203). However, the ACT subgroup demonstrated a small but statistically reliable effect at this stage (*k* = 4, *g* = 0.24, 95% CI [0.06, 0.42], *p* = .010). At long-term follow-up, a small pooled effect was observed (*k* = 3, *g* = 0.20, 95% CI [0.001, 0.405], *p* = .049), although this estimate was based on limited evidence derived exclusively from guided ACT interventions (Fig 2).

**Fig 2:**
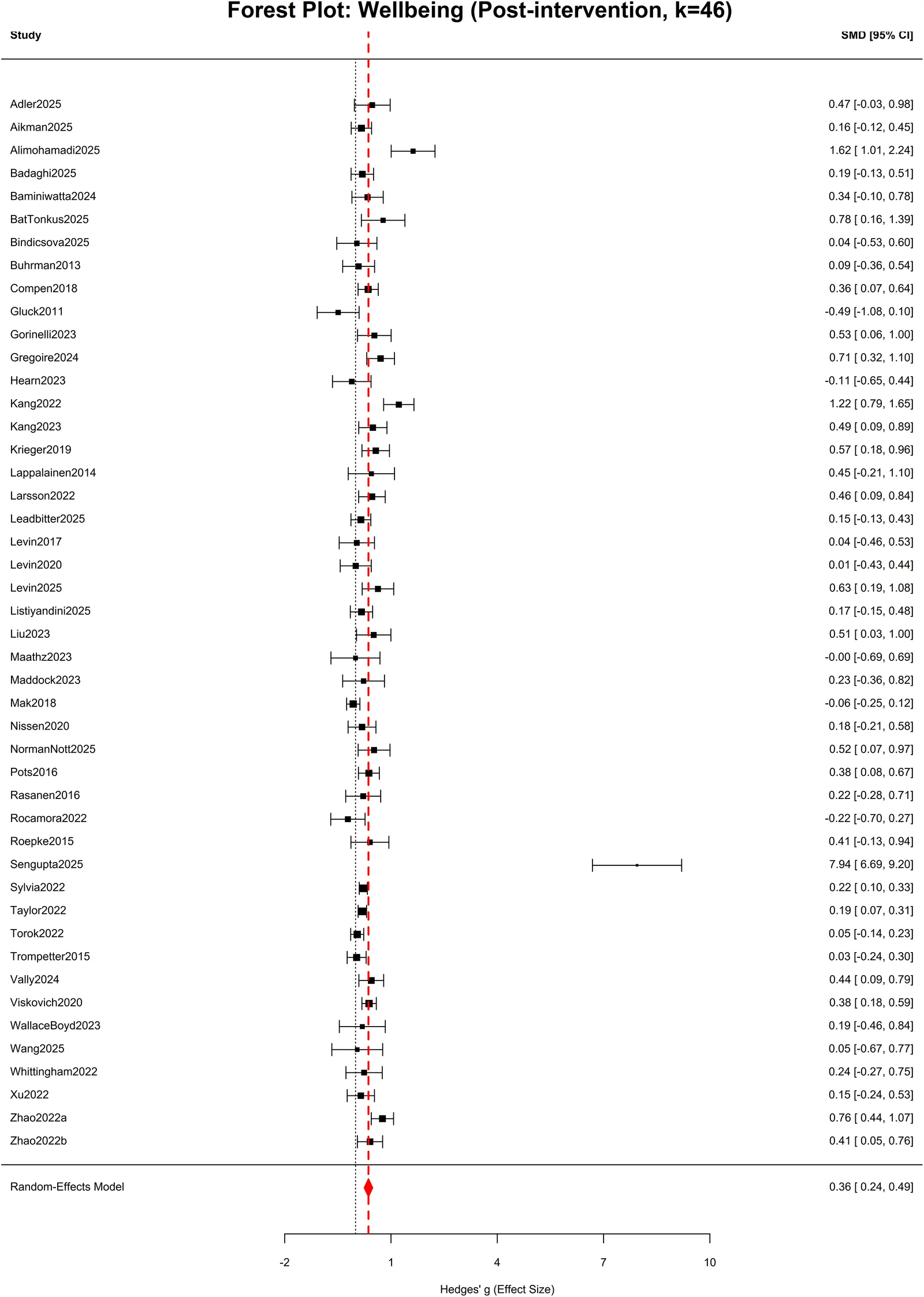
Wellbeing forest plots (immediate post-intervention).

#### Depression.

Regarding depressive symptoms, immediate post-intervention results indicated moderate reductions overall (*k* = 110, *g* = 0.44, 95% CI [0.37, 0.52], *p* < .001), with substantial heterogeneity (*I²* = 78%, PI [−0.21, 1.10]). Effects were significantly smaller when interventions were compared with active controls (*k* = 30, *g* = 0.20, 95% CI [0.11, 0.30], *p* < .001) than inactive comparators (*k* = 89, *g* = 0.51, 95% CI [0.42, 0.60], *p* < .001), with comparator type supported as a significant moderator in meta-regression (*QM* = 9.42, *p* = .003).

Reductions were robust across all populations, including Healthy (*k* = 37, *g* = 0.46, 95% CI [0.35, 0.56], *p* < .001), mental health condition (*k* = 36, *g* = 0.44, 95% CI [0.31, 0.56], *p* < .001), and medical condition samples (*k* = 37, *g* = 0.42, 95% CI [0.24, 0.60], *p* < .001), with population type not supported as a significant moderator in meta-regression. Effects were similar in guided (*k* = 65, *g* = 0.47, 95% CI [0.35, 0.58], *p* < .001) and unguided formats (*k* = 45, *g* = 0.42, 95% CI [0.33, 0.52], *p* < .001), with guidance also not supported as a significant immediate post-intervention moderator. Statistically reliable reductions were observed across several intervention families, including ACT (*k* = 53, *g* = 0.40, 95% CI [0.31, 0.50], *p* < .001), broader mindfulness-based interventions (*k* = 31, *g* = 0.43, 95% CI [0.30, 0.57], *p* < .001), MBCT (*k* = 7, *g* = 0.73, 95% CI [0.40, 1.07], *p* < .001), mindfulness-CBT integrative programmes (*k* = 6, *g* = 0.34, 95% CI [0.09, 0.58], *p* = .006), and DBT-informed interventions (*k* = 5, *g* = 0.33, 95% CI [0.08, 0.58], *p* = .010). However, intervention family was a significant moderator in meta-regression, driven primarily by the single compassion-focused trial with an unusually large effect [48]. Exclusion of this study did not materially change the pooled estimate.

At short-term follow-up, reductions in depression were maintained (*k* = 41, *g* = 0.38, 95% CI [0.27, 0.48], *p* < .001; *I²* = 69%). Effects were observed when interventions were compared with both active controls (*g* = 0.20, *p* = .002) and inactive controls (*g* = 0.44, *p* < .001), and across all population subgroups. Both guided interventions (*k* = 20, *g* = 0.49, 95% CI [0.35, 0.64], *p* < .001) and unguided formats (*k* = 21, *g* = 0.28, 95% CI [0.14, 0.42], *p* < .001) showed statistically reliable effects at this stage. By medium-term follow-up, a smaller but statistically reliable effect persisted (*k* = 19, *g* = 0.30, 95% CI [0.14, 0.46], *p* < .001). Effects were statistically reliable when compared with inactive controls (*g* = 0.31, *p* < .001), while the active-control estimate was similar in magnitude but not statistically reliable (*k* = 8, *g* = 0.28, *p* = .087). ACT (*k* = 9, *g* = 0.29, *p* = .013) and MBCT (*k* = 5, *g* = 0.44, *p* = .029) continued to demonstrate benefits. At long-term follow-up, the overall pooled estimate was small and imprecise (*k* = 5, *g* = 0.08, 95% CI [−0.30, 0.46], *p* = .670), providing limited evidence for maintenance beyond the medium term (Fig 3).

**Fig 3:**
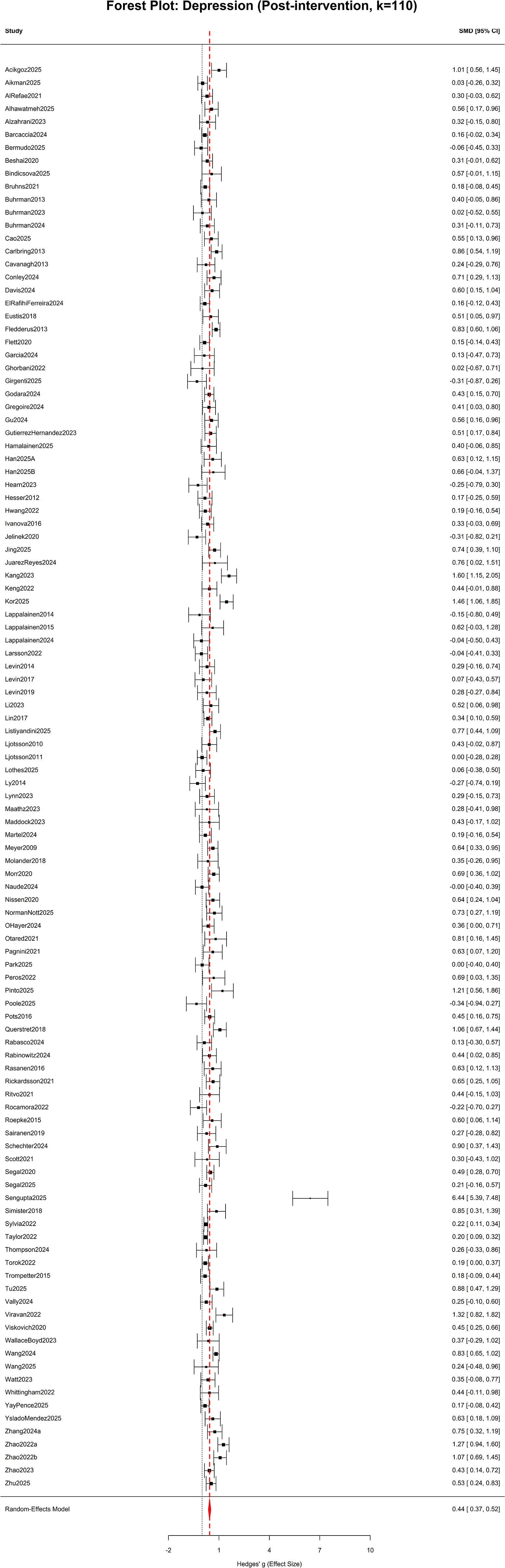
Depression forest plots (immediate post-intervention).

### Secondary outcome measures

#### Anxiety.

For anxiety, digital third-wave interventions were associated with significant reductions immediately post-intervention (*k* = 93, *g* = 0.40, 95% CI [0.32, 0.48], *p* < .001). Effects were significantly smaller when interventions were compared with active controls (*k* = 29, *g* = 0.16, 95% CI [0.11, 0.21], *p* < .001) than inactive controls (*k* = 74, *g* = 0.47, 95% CI [0.37, 0.57], *p* < .001), consistent with comparator type being a significant moderator in meta-regression (*QM* = 9.96, *p* = .002).

Reductions were evident across healthy (*k* = 37, *g* = 0.36, 95% CI [0.27, 0.46], *p* < .001), mental health condition (*k* = 26, *g* = 0.44, 95% CI [0.27, 0.62], *p* < .001), and medical condition samples (*k* = 30, *g* = 0.37, 95% CI [0.18, 0.56], *p* < .001). Effects were also evident in both guided (*k* = 61, *g* = 0.42, 95% CI [0.31, 0.53], *p* < .001) and unguided interventions (*k* = 32, *g* = 0.37, 95% CI [0.25, 0.48], *p* < .001). ACT (*k* = 45, *g* = 0.38, 95% CI [0.27, 0.49], *p* < .001) and broader mindfulness-based interventions (*k* = 27, *g* = 0.39, 95% CI [0.26, 0.52], *p* < .001) both demonstrated statistically reliable reductions. Intervention family was a significant moderator in meta-regression, driven primarily by the single compassion-focused trial with an unusually large effect [48], although leave-one-out sensitivity analysis showed that its exclusion did not materially alter the pooled anxiety effect.

At short-term follow-up, reductions in anxiety were maintained (*k* = 36, *g* = 0.40, 95% CI [0.27, 0.53], *p* < .001), with effects observed against both active (*g* = 0.21, *p* = .009) and inactive controls (*g* = 0.46, *p* < .001). Both guided interventions (*k* = 20, *g* = 0.48, 95% CI [0.32, 0.64], *p* < .001) and unguided formats (*k* = 16, *g* = 0.30, 95% CI [0.10, 0.51], *p* = .004) showed statistically reliable effects at this timepoint. At medium-term follow-up, the pooled effect was smaller but remained statistically reliable (*k* = 15, *g* = 0.23, 95% CI [0.13, 0.34], *p* < .001). Effects were observed against inactive controls (*g* = 0.28, *p* < .001), whereas the active-control estimate was not statistically significant (*k* = 7, *g* = 0.15, 95% CI [−0.03, 0.34], *p* = .098). Guided interventions retained an effect (*k* = 14, *g* = 0.25, 95% CI [0.14, 0.37], *p* < .001). By long-term follow-up, the overall estimate was small and not statistically significant (*k* = 3, *g* = 0.05, 95% CI [−0.36, 0.47], *p* = .800), reflecting limited evidence at this stage.

#### Stress.

Immediate post-intervention analyses for stress revealed moderate reductions overall (*k* = 66, *g* = 0.46, 95% CI [0.36, 0.55], *p* < .001), but with substantial heterogeneity (*I²* = 80%, PI [−0.20, 1.11]). Effects were observed against both inactive controls (*k* = 50, *g* = 0.52, 95% CI [0.40, 0.63], *p* < .001) and active controls (*k* = 23, *g* = 0.30, 95% CI [0.18, 0.42], *p* < .001). Reductions were also observed across Healthy (*k* = 43, *g* = 0.42, 95% CI [0.33, 0.50], *p* < .001) and mental health condition samples (*k* = 12, *g* = 0.44, 95% CI [0.26, 0.63], *p* < .001), while the estimate for medical condition samples was larger but highly heterogeneous (*k* = 11, *g* = 0.76, 95% CI [0.21, 1.32], *p* = .007). Effects were evident in both guided (*k* = 35, *g* = 0.50, 95% CI [0.33, 0.68], *p* < .001) and unguided formats (*k* = 31, *g* = 0.43, 95% CI [0.33, 0.53], *p* < .001), and for both broader mindfulness-based interventions (*k* = 26, *g* = 0.47, 95% CI [0.36, 0.58], *p* < .001) and ACT (*k* = 22, *g* = 0.34, 95% CI [0.23, 0.45], *p* < .001).

At short-term follow-up, reductions in stress were maintained overall (*k* = 25, *g* = 0.35, 95% CI [0.20, 0.50], *p* < .001; *I²* = 80%, PI [−0.28, 0.99]). Effects were reliable against inactive controls (*g* = 0.51, *p* < .001), but not active controls (*g* = 0.07, *p* = .094), with comparator type supported as a significant short-term moderator in meta-regression (*QM* = 14.03, *p* < .001). Effects were also observed for both guided (*g* = 0.43) and unguided formats (*g* = 0.26). At medium-term follow-up, a moderate pooled effect was observed (*k* = 10, *g* = 0.30, 95% CI [0.16, 0.44], *p* < .001), including against both inactive controls (*k* = 5, *g* = 0.31, 95% CI [0.14, 0.48], *p* < .001) and active controls (*k* = 5, *g* = 0.31, 95% CI [0.08, 0.55], *p* = .009). Guided interventions retained an effect (*k* = 8, *g* = 0.37, 95% CI [0.21, 0.52], *p* < .001). By long-term follow-up, the overall estimate was negligible (*k* = 3, *g* = 0.02, 95% CI [−0.53, 0.58], *p* = .931), indicating insufficient evidence to support long-term maintenance of stress reduction.

#### Psychological flexibility.

Immediately post-intervention, digital third-wave interventions were associated with improvements in psychological flexibility (*k* = 51, *g* = 0.41, 95% CI [0.27, 0.54], *p* < .001). Improvements were evident relative to inactive controls (*k* = 43, *g* = 0.47, 95% CI [0.32, 0.61], *p* < .001), but not relative to active controls (*k* = 11, *g* = 0.05, 95% CI [−0.16, 0.27], *p* = .614), consistent with comparator type being a significant moderator in meta-regression (*QM* = 5.37, *p* = .021). Gains were observed across Healthy (*k* = 14, *g* = 0.38, 95% CI [0.18, 0.57], *p* < .001), mental health (*k* = 19, *g* = 0.46, 95% CI [0.19, 0.72], *p* < .001), and medical condition samples (*k* = 18, *g* = 0.36, 95% CI [0.13, 0.58], *p* = .002). Both guided (*k* = 33, *g* = 0.34, 95% CI [0.17, 0.51], *p* < .001) and unguided formats (*k* = 18, *g* = 0.52, 95% CI [0.32, 0.73], *p* < .001) showed effects. ACT accounted for the majority of trials and demonstrated a statistically reliable effect (*k* = 47, *g* = 0.38, 95% CI [0.24, 0.52], *p* < .001).

At short-term follow-up, improvements remained statistically significant (*k* = 21, *g* = 0.54, 95% CI [0.25, 0.83], *p* < .001), with effects observed relative to both active (*g* = 0.35, *p* = .001) and inactive controls (*g* = 0.58, *p* = .001). Improvements were evident in Healthy (*g* = 0.46, *p* < .001) and mental health samples (*g* = 0.87, *p* = .004), while the estimate for medical condition samples was not statistically significant (*g* = 0.45, *p* = .101). Effects were also observed in both guided (*g* = 0.38, *p* = .010) and unguided interventions (*g* = 0.96, *p* = .003). By medium-term follow-up, the pooled estimate was small and not statistically distinguishable from zero (*k* = 9, *g* = 0.05, 95% CI [−0.17, 0.26], *p* = .680). At long-term follow-up, the pooled estimate remained small and not statistically significant (*k* = 4, *g* = 0.08, 95% CI [−0.25, 0.41], *p* = .648).

#### Quality of life.

Quality of life improved immediately post-intervention overall (*k* = 32, *g* = 0.30, 95% CI [0.19, 0.42], *p* < .001), with effects evident relative to inactive controls (*k* = 27, *g* = 0.32, 95% CI [0.19, 0.45], *p* < .001), but not active controls (*k* = 6, *g* = 0.15, 95% CI [−0.06, 0.36], *p* = .170). Gains were observed in mental health (*k* = 11, *g* = 0.25, 95% CI [0.04, 0.47], *p* = .023) and medical condition samples (*k* = 17, *g* = 0.31, 95% CI [0.14, 0.48], *p* < .001), and effects were also evident in both guided (*k* = 25, *g* = 0.27, 95% CI [0.15, 0.39], *p* < .001) and unguided interventions (*k* = 7, *g* = 0.42, 95% CI [0.11, 0.73], *p* = .008). ACT accounted for the majority of trials and showed a statistically reliable effect (*k* = 22, *g* = 0.30, 95% CI [0.14, 0.45], *p* < .001).

At short-term follow-up, improvements remained statistically significant (*k* = 11, *g* = 0.47, 95% CI [0.15, 0.78], *p* = .003). Effects were evident relative to inactive controls (*k* = 8, *g* = 0.58, 95% CI [0.19, 0.96], *p* = .003), whereas estimates against active controls were not statistically reliable (*k* = 3, *g* = 0.13, 95% CI [−0.17, 0.44], *p* = .395). Improvements were observed in medical condition samples (*k* = 6, *g* = 0.73, 95% CI [0.28, 1.17], *p* = .001), and guided interventions retained an effect (*k* = 8, *g* = 0.48, 95% CI [0.10, 0.86], *p* = .013). By medium-term (*k* = 7, *g* = 0.08, 95% CI [−0.11, 0.26], *p* = .409) and long-term follow-up (*k* = 3, *g* = 0.09, 95% CI [−0.30, 0.48], *p* = .635), pooled estimates were small and not statistically distinguishable from zero, suggesting limited durability for this outcome.

#### Key results summary.

Across outcomes, digitally delivered third-wave interventions produced small-to-moderate improvements immediately post-intervention, with pooled effects ranging from *g* = 0.30 for QoL (95% CI [0.19, 0.42]) to *g* = 0.46 for stress (95% CI [0.36, 0.55]). Effects were also observed for depression (*g* = 0.44, 95% CI [0.37, 0.52]), psychological flexibility (*g* = 0.41, 95% CI [0.27, 0.54]), anxiety (*g* = 0.40, 95% CI [0.32, 0.48]), and wellbeing (*g* = 0.36, 95% CI [0.24, 0.49]). Heterogeneity was substantial for most symptom and process outcomes (I² typically >75%), whereas QoL showed comparatively lower heterogeneity (*I²* = 58% immediately post-intervention). Prediction intervals were notably wide across all outcomes, crossing the null in every instance, which indicates that while pooled effects are positive, the expected effects in any single future application remain highly variable.

At short-term follow-up, statistically supported improvements persisted for depression, anxiety, stress, psychological flexibility, wellbeing, and QoL. At medium-term follow-up, statistical support was maintained for depression, anxiety, and stress, though effects for wellbeing, psychological flexibility, and QoL were no longer statistically supported at this interval. Long-term data were sparse across the literature, however a statistically supported effect was observed for wellbeing, whereas depression, anxiety, stress, psychological flexibility, and QoL did not demonstrate sustained improvement at the longest follow-up intervals.

These findings indicate that digital third-wave interventions yield immediate post-intervention benefits across both symptom-focused and wellbeing-oriented domains. While evidence suggests these benefits are maintained into the medium-term for depression, anxiety, and stress, longer-term evidence remains limited and requires further investigation. Detailed results for all prespecified subgroup analyses across all outcome domains and time windows are provided in Text A and Tables A–D in S7 Appendix (Table 3).

**Table 3:** Immediate post-intervention pooled effect sizes (Hedges’ *g*), heterogeneity (*I²*), and prediction intervals by outcome and timepoint.

Outcome	Timepoint	*k*	Hedges’ *g*	95% *CI*	*p*	*I²* (%)	Prediction interval
**Wellbeing**	Immediate post-intervention	46	0.36	0.24 to 0.49	<.001	82	−0.34 to 1.07
	Short-term	17	0.18	0.05 to 0.31	.006	66	−0.23 to 0.59
	Medium-term	7	0.12	−0.06 to 0.30	.203	56	−0.27 to 0.50
	Long-term	3	0.20	0.001 to 0.405	.049	0	0.00 to 0.40
**Depression**	Immediate post-intervention	110	0.44	0.37 to 0.52	<.001	78	−0.21 to 1.10
	Short-term	41	0.38	0.27 to 0.48	<.001	69	−0.14 to 0.89
	Medium-term	19	0.30	0.14 to 0.46	<.001	74	−0.30 to 0.89
	Long-term	5	0.08	−0.30 to 0.46	.670	75	−0.73 to 0.90
**Anxiety**	Immediate post-intervention	93	0.40	0.32 to 0.48	<.001	79	−0.25 to 1.04
	Short-term	36	0.40	0.27 to 0.53	<.001	80	−0.26 to 1.06
	Medium-term	15	0.23	0.13 to 0.34	<.001	35	−0.02 to 0.49
	Long-term	3	0.05	−0.36 to 0.47	.800	76	−0.70 to 0.80
**Stress**	Immediate post-intervention	66	0.46	0.36 to 0.55	<.001	80	−0.20 to 1.11
	Short-term	25	0.35	0.20 to 0.50	<.001	80	−0.28 to 0.99
	Medium-term	10	0.30	0.16 to 0.44	<.001	37	0.01 to 0.59
	Long-term	3	0.02	−0.53 to 0.58	.931	77	−0.98 to 1.02
**Psychological Flexibility**	Immediate post-intervention	51	0.41	0.27 to 0.54	<.001	81	−0.44 to 1.26
	Short-term	21	0.54	0.25 to 0.83	<.001	88	−0.70 to 1.78
	Medium-term	9	0.05	−0.17 to 0.26	.680	63	−0.49 to 0.58
	Long-term	4	0.08	−0.25 to 0.41	.648	55	−0.50 to 0.66
**Quality of Life**	Immediate post-intervention	32	0.30	0.19 to 0.42	<.001	58	−0.19 to 0.79
	Short-term	11	0.47	0.15 to 0.78	.003	74	−0.47 to 1.40
	Medium-term	7	0.08	−0.11 to 0.26	.409	28	−0.23 to 0.39
	Long-term	3	0.09	−0.30 to 0.48	.635	80	−0.61 to 0.80

### Meta-regression and sensitivity analyses

Random-effects meta-regression models were conducted to investigate source-specific heterogeneity across eligible outcome-timepoint analyses (see Text A and Table A in S8 Appendix). To address dependencies in multi-arm trials, a Split-N correction was applied to shared intervention group sample sizes to ensure accurate variance estimation and prevent unit-of-analysis errors. Comparator type was a significant moderator for several outcomes. Immediately post-intervention, meta-regression confirmed significantly larger effect sizes in comparisons against inactive controls relative to active controls for depression (*QM* = 9.42, *p* = .003) and anxiety (*QM* = 9.96, *p* = .002). At short-term follow-up, comparator type significantly moderated depression (*QM* = 4.45, *p* = .041), wellbeing (*QM* = 5.16, *p* = .037), and stress (*QM* = 14.03, *p* < .001). In contrast, population type was not a significant predictor for any primary outcome immediately post-intervention (all *p* values > .35), providing statistical support for the transdiagnostic efficacy of these interventions across healthy, medical, and mental health samples. These moderator findings should be interpreted as exploratory, particularly for the short-term comparator models for wellbeing and stress, where active-control subgroup estimates were based on relatively few comparisons (*k* = 6 and *k* = 9, respectively).

Regarding intervention characteristics, intervention family was a significant moderator for depression and anxiety (*p* < .001). Examination of regression coefficients indicated that these moderator findings were driven by exceptionally large effect sizes in the compassion-focused therapy subgroup, which comprised a single outlier trial [48]. Leave-one-out analyses showed that excluding this study reduced heterogeneity but did not alter the direction or statistical significance of the pooled depression or anxiety effects. Therapist involvement (guidance) did not significantly moderate any outcome immediately post-intervention (all *p* values were .236 or greater), but emerged as a significant *p*redictor for depression outcomes at short-term follow-up (*QM* = 4.17, *p* = .048), with significantly smaller effects in unguided formats (*B* = -0.21).

The robustness of these findings was confirmed through multiple sensitivity analyses. Excluding studies at high risk of bias did not significantly alter the magnitude or direction of pooled effects for any primary outcome (see Text A and Tables A–B in S9 Appendix). Similarly, effect sizes derived from pre-post change scores were consistent with those from immediate post-intervention comparisons (*g* differences < 0.05; see Text A and Table A in S10 Appendix). Fixed-effect and random-effects models produced effects in the same direction across all main analyses, with immediate post-intervention estimates remaining statistically significant under both models (fixed-effect g range = 0.26 to 0.43; random-effects g range = 0.30 to 0.46; see Text A and Table A in S11 Appendix). Funnel plots for all immediate post-intervention outcomes are presented in Figs B, D, F, H, J, and L in S12 Appendix. Visual inspection suggested possible small-study effects for some outcomes, particularly where isolated large positive effects were present, although this pattern was not consistent across outcomes. These findings did not suggest that the pooled conclusions were explained by small-study effects alone. For the outlier trial [48], the published means and standard deviations, outcome direction, and Hedges’ g calculations were rechecked. No extraction, direction-coding, or calculation errors were identified. Finally, leave-one-out analyses confirmed that exclusion of this trial reduced heterogeneity but did not alter the direction or statistical significance of the primary pooled effects, including wellbeing (*g* = 0.30, 95% CI [0.22, 0.39]), depression (*g* = 0.42, 95% CI [0.36, 0.49]), anxiety (*g* = 0.37, 95% CI [0.30, 0.44]), and stress (*g* = 0.42, 95% CI [0.34, 0.49]) (see Text A and Table A in S13 Appendix).

### Certainty of evidence

The certainty of evidence for immediate post-intervention outcomes was assessed using the GRADE framework (Table 4). Evidence was rated as moderate for wellbeing, depression, anxiety, stress, and psychological flexibility, and low for QoL. For wellbeing, depression, anxiety, stress, and psychological flexibility, the main limitation was inconsistency, as heterogeneity was substantial (*I²* > 75%) and prediction intervals crossed the null, which resulted in a one-level GRADE downgrade for serious inconsistency. A second downgrade for inconsistency was not applied because influence diagnostics and sensitivity analyses indicated that pooled effects remained statistically significant after exclusion of influential studies. QoL was not downgraded for inconsistency because heterogeneity was lower (*I²* = 57.9%), but it retained the risk-of-bias downgrade from the original assessment because 7 of 32 contributing effect estimates were from studies rated at high risk of bias. QoL was also downgraded for imprecision because the lower confidence limit (0.19) extended below the prespecified smallest important effect threshold (SMD = 0.20). No serious concerns were identified regarding indirectness. Publication bias was considered in the GRADE assessment, but no additional downgrades were applied because funnel-plot asymmetry was inconsistent across outcomes, fixed-effect sensitivity analyses did not alter the primary conclusions, and possible asymmetry was difficult to distinguish from substantial heterogeneity and identified outlying effects.

**Table 4:** GRADE Summary of Findings for primary outcomes (immediate post-intervention).

Outcome	Studies (*k*)	Participants (*N*)	Pooled effect (Hedges’ *g*, 95% *CI*)	Heterogeneity (*I²*)	Certainty (GRADE)	Downgrade rationale
**Wellbeing**	46	7,485	0.36 (0.24 to 0.49)	82.3%	**Moderate**	−1 inconsistency (substantial heterogeneity; *PI* crosses no effect)
**Depression**	110	15,102	0.44 (0.37 to 0.52)	77.8%	**Moderate**	−1 inconsistency (substantial heterogeneity; *PI* crosses no effect)
**Anxiety**	93	13,812	0.40 (0.32 to 0.48)	78.7%	**Moderate**	−1 inconsistency (substantial heterogeneity; *PI* crosses no effect)
**Stress**	66	10,544	0.46 (0.36 to 0.55)	79.9%	**Moderate**	−1 inconsistency (substantial heterogeneity; *PI* crosses no effect)
**Psychological flexibility**	51	5,492	0.41 (0.27 to 0.54)	81.4%	**Moderate**	−1 inconsistency (substantial heterogeneity; *PI* crosses no effect)
**QoL**	32	3,184	0.30 (0.19 to 0.42)	57.9%	**Low**	−1 risk of bias (high proportion of high-RoB results); −1 imprecision (lower CI extended below prespecified SMD 0.20 threshold)

## Discussion

This review synthesised evidence from 147 RCTs (26,391 participants) evaluating digitally delivered third-wave, process-based psychological interventions across healthy samples and samples with mental and medical conditions. Immediately post-intervention, digital third-wave interventions were associated with statistically supported improvements in wellbeing (*g* = 0.36), reductions in depressive symptoms (*g* = 0.44), anxiety (*g* = 0.40) and stress (*g* = 0.46), alongside improvements in psychological flexibility (*g* = 0.41) and QoL (*g* = 0.30). Across outcomes, pooled effects were small-to-moderate in magnitude and consistently favoured intervention immediately post-intervention, indicating reliable average benefits across both symptom-focused and wellbeing-focused domains.

Heterogeneity was substantial across most immediate post-intervention syntheses, and prediction intervals were often wide and crossed the null. This does not negate the pooled mean effects, but indicates that average effects should not be treated as uniform expectations across settings. Confidence intervals quantify uncertainty around the pooled mean, whereas prediction intervals reflect the likely dispersion of true effects across settings, populations and implementations [49–51]. Practically, these results indicate that digital third-wave interventions are beneficial on average, but that realised effects are likely to vary meaningfully across contexts, consistent with substantial variation in programme content, delivery platforms, guidance intensity, engagement supports, and comparator intensity across trials.

These interpretations are further supported by prespecified sensitivity analyses and certainty-of-evidence assessments. Across outcomes, exclusion of results judged at overall high risk of bias generally produced pooled estimates that were similar in magnitude and direction to the primary analyses. However, robustness varied by timepoint. While immediate post-intervention results remained stable, the short-term wellbeing estimate was attenuated and was no longer statistically significant after exclusion of high risk-of-bias results (*p =* .051). The medium-term QoL estimate was also reduced but was not statistically significant in either the primary or sensitivity analysis. Influence diagnostics identified specific outliers contributing disproportionately to heterogeneity, most notably a trial evaluating Mindful Self-Compassion [48] which reported exceptionally large effect sizes. Leave-one-out analyses confirmed that while removing such outliers substantially reduced residual heterogeneity (e.*g*., reducing *I²* for wellbeing from 82% to 60%), it did not alter the statistical significance or direction of the primary pooled effects. Sensitivity analyses based on change-from-baseline effect sizes yielded comparable results across a range of plausible pre–post correlation assumptions (*r* = 0.30–0.70), confirming that conclusions were not dependent on the choice of effect metric or imputation strategy.

Consistent with these findings, certainty of evidence was rated as moderate for five immediate post-intervention outcomes and low for QoL using the GRADE framework. Downgrading was driven primarily by statistical inconsistency, reflecting substantial residual heterogeneity and wide prediction intervals. For QoL, certainty was also downgraded for risk of bias and imprecision because the lower confidence limit fell just below the prespecified smallest important effect threshold. This pattern is expected in a synthesis spanning diverse populations, intervention formats and comparator contexts, and indicates that while average effects are reliable, their magnitude is likely to vary across settings. From a decision-making perspective, the results support cautious confidence in the presence of benefit immediately post-intervention, alongside recognition that implementation context remains an important determinant of realised impact.

One consistent finding was the pattern of smaller pooled effects against active controls than against inactive controls, alongside formal moderation support in several key outcome-timepoint subgroups. This means that pooled average effects should be interpreted alongside comparator intensity, rather than as uniform estimates of superiority over credible alternatives. Comparator type significantly moderated immediate post-intervention effects for depression, anxiety, and psychological flexibility, with larger effects in inactive-control trials in each case. This pattern was also observed at short-term follow-up for depression, wellbeing, and stress. These results mirror the subgroup syntheses. Immediately post-intervention, effects against active controls were smaller but statistically supported for wellbeing (*g* = 0.15), depression (*g* = 0.20), anxiety (*g* = 0.16), and stress (*g* = 0.30), while QoL was smaller and not statistically supported (*g* = 0.15). Inactive-control contrasts were consistently larger across all outcomes. This pattern is expected, as active controls often include credible therapeutic ingredients such as structured activity, psychoeducation, self-monitoring or skills practice. Importantly, several outcomes retained statistically supported incremental benefits even under active-control conditions, strengthening the real-world relevance of these findings where choices are more often between plausible alternatives than between intervention and no support. This comparator separation also represents a methodological advance over syntheses dominated by minimal-control contrasts [52].

These findings align with broader evidence that waitlist and minimal-control designs can inflate apparent psychotherapy effects relative to care-as-usual or other credible comparators [53]. In the present review, inactive-control estimates remain informative for contexts characterised by limited provision, long waits or minimal support. However, the active-control estimates provide more conservative and arguably more realistic benchmarks for settings where credible alternatives are available. Notably, the moderation by comparator type was also observed at short-term follow-up for depression, wellbeing, and stress, indicating that inflation from minimal controls is a robust and pervasive factor influencing effect magnitude into the early follow-up period.

Across outcomes, follow-up syntheses indicated partial attenuation over time, with important differences across domains. Reductions in depressive symptoms, anxiety and stress remained statistically supported at short- and medium-term follow-up, although effect sizes were smaller than immediately post-intervention, whereas long-term estimates were small and imprecise and did not demonstrate sustained statistical support. Wellbeing and QoL showed statistically supported improvements immediately post-intervention and short-term follow-up, but statistical support was not maintained at the medium-term interval for either outcome. A small but statistically supported effect for wellbeing was observed at long-term follow-up, although this estimate was based on only three trials and should therefore be interpreted cautiously. In contrast, psychological flexibility demonstrated a distinct trajectory, with robust effects immediately post-intervention and short-term follow-up that were not sustained at medium- or long-term follow-up. This divergence between the maintenance of symptom reduction and the attenuation of process-based gains may partly reflect limited follow-up power and measurement heterogeneity. Because this review synthesised aggregate outcome trajectories rather than within-person mediation, these findings should not be interpreted as demonstrating a mechanism-outcome gap. Rather, they are consistent with ACT mediation reviews and internet-based ACT syntheses that support psychological flexibility as a plausible treatment process while emphasising the need for stronger longitudinal and process-measure evidence [8,54–56].

Moderator analyses helped identify sources of the substantial heterogeneity observed across outcomes. Intervention family significantly moderated immediate post-intervention effects for depression (QM = 126.12, p < .001) and anxiety (QM = 106.30, p < .001). These findings should be interpreted cautiously because several intervention-family categories had small study counts. For depression and anxiety, the compassion-focused category included one trial, which evaluated Mindful Self-Compassion in participants with chronic skin conditions and elevated psychological distress [48]. The estimates for this category were very large (depression: g = 6.44; anxiety: g = 5.49), making this single trial a major contributor to the significant intervention-family moderation. These estimates are unlikely to represent typical effects for compassion-focused digital interventions and should therefore be treated as hypothesis-generating until replicated in larger, methodologically robust trials.

Guidance did not significantly moderate any immediate post-intervention outcome, indicating that therapist involvement was not a general determinant of effect size at programme completion. The only statistically supported guidance moderation was observed for depression at short-term follow-up (QM = 4.17, p = .048), where effects were smaller in unguided formats (B = -0.21). This suggests that therapist involvement may be more relevant to the maintenance of depressive symptom gains than to immediate intervention effects, although this pattern was not consistent across outcomes. Supportive accountability offers one explanation for this finding, as human support may improve engagement through accountability and expectations to a trusted support person [57]. Broader digital mental health literature also suggests that social support and persuasive design features may be relevant to engagement and adherence, while showing that their effects on clinical outcomes remain uncertain and difficult to evaluate because engagement metrics are reported inconsistently [58,59]. Future trials should report guidance content, adherence, and engagement metrics more consistently to clarify when human support adds value to digital third-wave interventions.

Population type moderated anxiety and depression outcomes at short-term follow-up, indicating that clinical samples (mental health and medical conditions) may derive larger short-term benefits than healthy samples. However, no statistically significant moderation was observed immediately post-intervention, suggesting that the transdiagnostic logic of third-wave and process-based approaches holds during the active treatment phase [8,10,60]. It also suggests that implementation decisions may be better guided by factors such as comparator context, intervention modality and delivery supports than by broad population category alone.

Several limitations constrain inference and point to priorities for future research. First, most studies were judged to raise risk-of-bias concerns, with 120 of 147 trials (82%) rated as ‘some concerns’, 24 (16%) as ‘high risk’, and 3 (2%) as ‘low risk’. Outcomes were also primarily self-reported, reducing certainty about effect magnitude. This profile limits confidence in the exact magnitude of effects, particularly because some concerns commonly reflected incomplete reporting of allocation concealment, self-reported outcomes in unblinded behavioural trials, limited prespecification of analyses, and missing outcome data. However, sensitivity analyses excluding high-risk studies generally produced effects of similar magnitude and direction, supporting the robustness of the main immediate post-intervention conclusions. Accordingly, the findings should be interpreted as evidence of probable average benefit rather than precise implementation benchmarks. Second, follow-up evidence, particularly long-term, remains sparse across outcomes, limiting precision about durability and especially about incremental benefits against active comparators at later timepoints. Third, the evidence base is dominated by ACT-based and mindfulness-based programmes, with comparatively few trials of DBT-informed and compassion-focused interventions, limiting the precision and generalisability of modality-specific estimates. Potential small-study effects also remain a limitation, particularly for outcomes with visible funnel-plot asymmetry (see Figs B, D, F, H, J, and L in S12 Appendix). However, fixed-effect sensitivity analyses and leave-one-out analyses indicated that the direction and statistical significance of the main pooled effects were not dependent on these patterns (see Text A and Table A in S11 Appendix and Text A and Table A in S13 Appendix), suggesting that small-study effects are more relevant to the interpretation of effect magnitude than to the presence of an overall benefit. Module completion, platform usage, adherence, and broader engagement metrics were also reported inconsistently across trials and therefore could not be examined in meta-regression, despite their likely relevance to digital intervention effects [15,59,61]. Finally, wide prediction intervals and substantial heterogeneity indicate that average effects should not be treated as uniform expectations. Instead, heterogeneity should be treated as a target for explanation, including better reporting of engagement, adherence, comparator content and implementation conditions.

### Clinical implications

Overall, these findings support digitally delivered third-wave interventions as scalable programmes that yield reliable average improvements in wellbeing, depressive symptoms, anxiety, stress, psychological flexibility and QoL immediately post-intervention. Several benefits persisted across follow-up windows, although effects were consistently smaller when interventions were evaluated against active comparators. The most decision-relevant messages are that pooled effects are consistently smaller against active controls than inactive controls, and that effects do not differ systematically across broad population types, supporting a transdiagnostic framing in digital delivery contexts. Future trials should prioritise well-specified active comparators, longer follow-up with adequate power, consistent reporting of adherence and engagement metrics, and mechanistic analyses testing whether within-person changes in theorised process targets, particularly psychological flexibility, account for downstream changes in symptoms and wellbeing, and whether such process changes are maintained or require structured booster strategies over time.

## Supporting information

S1 Appendix PRISMA 2020 checklist. (DOCX)

S2 Appendix Search strategies. (DOCX)

S3 Appendix Extraction sheet. (DOCX)

S4 Appendix Outcome measurement hierarchy. (DOCX)

S5 Appendix Risk of bias. (DOCX)

S6 Appendix Full reference list for the 147 studies included in this meta-analysis. (DOCX)

S7 Appendix Full subgroup analyses. (DOCX)

S8 Appendix Meta-regression results. (DOCX)

S9 Appendix High-risk-of-bias exclusion sensitivity analysis. (DOCX)

S10 Appendix Sensitivity analyses using change-score standardised mean differences and standard deviation imputations. (DOCX)

S11 Appendix Sensitivity analyses comparing fixed-effect and random-effects models. (DOCX)

S12 Appendix Full forest plots and funnel plots for all immediate post-intervention meta-analyses. (DOCX)

S13 Appendix Influence diagnostics and leave-one-out analyses. (DOCX)

## References

[1] Collaborators G. M. D. Global, regional, and national burden of 12 mental disorders in 204 countries and territories, 1990–2019: A systematic analysis for the Global Burden of Disease Study 2019. The Lancet Psychiatry. 2022;9(2):137–50. 10.1016/S2215-0366(21)00395-3 PMC8776563 35026139

[2] Bromet E, Andrade LH, Hwang I, Sampson NA, Alonso J, de Girolamo G, et al. Cross-national epidemiology of DSM-IV major depressive episode. BMC Med. 2011;9:90. doi: 10.1186/1741-7015-9-90 21791035 PMC3163615

[3] Marx W, Penninx BWJH, Solmi M, Furukawa TA, Firth J, Carvalho AF, et al. Major depressive disorder. Nat Rev Dis Primers. 2023;9(1):44. doi: 10.1038/s41572-023-00454-1 37620370

[4] Otte C, Gold SM, Penninx BW, Pariante CM, Etkin A, Fava M, et al. Major depressive disorder. Nat Rev Dis Primers. 2016;2:16065. doi: 10.1038/nrdp.2016.65 27629598

[5] Bandelow B, Michaelis S. Epidemiology of anxiety disorders in the 21st century. Dialogues Clin Neurosci. 2015;17(3):327–35. doi: 10.31887/DCNS.2015.17.3/bbandelow 26487813 PMC4610617

[6] Lee YY, Stockings EA, Harris MG, Doi SAR, Page IS, Davidson SK, et al. The risk of developing major depression among individuals with subthreshold depression: A systematic review and meta-analysis of longitudinal cohort studies. Psychol Med. 2019;49(1):92–102. doi: 10.1017/S0033291718000557 29530112

[7] Zhang R, Peng X, Song X, Long J, Wang C, Zhang C, et al. The prevalence and risk of developing major depression among individuals with subthreshold depression in the general population. Psychol Med. 2023;53(8):3611–20. doi: 10.1017/S0033291722000241 35156595 PMC10277767

[8] Hayes SC, Hofmann SG. The third wave of cognitive behavioral therapy and the rise of process-based care. World Psychiatry. 2017;16(3):245–6. doi: 10.1002/wps.20442 28941087 PMC5608815

[9] Hayes SC, Luoma JB, Bond FW, Masuda A, Lillis J. Acceptance and commitment therapy: Model, processes and outcomes. Behav Res Ther. 2006;44(1):1–25. doi: 10.1016/j.brat.2005.06.006 16300724

[10] Hofmann SG, Hayes SC. The future of intervention science: Process-based therapy. Clin Psychol Sci. 2019;7(1):37–50. doi: 10.1177/2167702618772296 PMC6350520 30713811

[11] Bond FW, Hayes SC, Baer RA, Carpenter KM, Guenole N, Orcutt HK, et al. Preliminary psychometric properties of the Acceptance and Action Questionnaire-II: A revised measure of psychological inflexibility and experiential avoidance. Behav Ther. 2011;42(4):676–88. doi: 10.1016/j.beth.2011.03.007 22035996

[12] Gloster AT, Meyer AH, Lieb R. Psychological flexibility as a malleable public health target: Evidence from a representative sample. Journal of Contextual Behavioral Science. 2017;6(2):166–71. doi: 10.1016/j.jcbs.2017.02.003

[13] Kashdan TB, Rottenberg J. Psychological flexibility as a fundamental aspect of health. Clin Psychol Rev. 2010;30(7):865–78. doi: 10.1016/j.cpr.2010.03.001 21151705 PMC2998793

[14] Grégoire S, Lachance L, Bouffard T, Dionne F. The use of acceptance and commitment therapy to promote mental health and school engagement in university students: A multisite randomized controlled trial. Behav Ther. 2018;49(3):360–72. doi: 10.1016/j.beth.2017.10.003 29704966

[15] Perski O, Blandford A, West R, Michie S. Conceptualising engagement with digital behaviour change interventions: A systematic review using principles from critical interpretive synthesis. Transl Behav Med. 2017;7(2):254–67. doi: 10.1007/s13142-016-0453-1 27966189 PMC5526809

[16] Kazdin AE, Blase SL. Rebooting psychotherapy research and practice to reduce the burden of mental illness. Perspect Psychol Sci. 2011;6(1):21–37. doi: 10.1177/1745691610393527 26162113

[17] Mohr DC, Ho J, Duffecy J, Baron KG, Lehman KA, Jin L, et al. Perceived barriers to psychological treatments and their relationship to depression. J Clin Psychol. 2010;66(4):394–409. doi: 10.1002/jclp.20659 20127795 PMC2907887

[18] Andersson G, Titov N. Advantages and limitations of Internet-based interventions for common mental disorders. World Psychiatry. 2014;13(1):4–11. doi: 10.1002/wps.20083 24497236 PMC3918007

[19] Firth J, Torous J, Nicholas J, Carney R, Pratap A, Rosenbaum S, et al. The efficacy of smartphone-based mental health interventions for depressive symptoms: A meta-analysis of randomized controlled trials. World Psychiatry. 2017;16(3):287–98. doi: 10.1002/wps.20472 28941113 PMC5608852

[20] Linardon J, Cuijpers P, Carlbring P, Messer M, Fuller-Tyszkiewicz M. The efficacy of app-supported smartphone interventions for mental health problems: A meta-analysis of randomized controlled trials. World Psychiatry. 2019;18(3):325–36. doi: 10.1002/wps.20673 31496095 PMC6732686

[21] Spijkerman MPJ, Pots WTM, Bohlmeijer ET. Effectiveness of online mindfulness-based interventions in improving mental health: A review and meta-analysis of randomised controlled trials. Clin Psychol Rev. 2016;45:102–14. doi: 10.1016/j.cpr.2016.03.009 27111302

[22] Han A, Kim TH. The effects of internet-based acceptance and commitment therapy on process measures: Systematic review and meta-analysis. J Med Internet Res. 2022;24(8):e39182. doi: 10.2196/39182 36040783 PMC9472046

[23] Klimczak KS, San Miguel GG, Mukasa MN, Twohig MP, Levin ME. A systematic review and meta-analysis of self-guided online acceptance and commitment therapy as a transdiagnostic self-help intervention. Cogn Behav Ther. 2023;52(3):269–94. doi: 10.1080/16506073.2023.2178498 36847182

[24] Churchill R, Moore THM, Furukawa TA, Caldwell DM, Davies P, Jones H, et al. “Third wave” cognitive and behavioural therapies versus treatment as usual for depression. Cochrane Database Syst Rev. 2013;2013(10):CD008705. doi: 10.1002/14651858.CD008705.pub2 24142810 PMC13035257

[25] Hayes SC, Hofmann SG, Stanton CE, Carpenter JK, Sanford BT, Curtiss JE, et al. The role of the individual in the coming era of process-based therapy. Behav Res Ther. 2019;117:40–53. doi: 10.1016/j.brat.2018.10.005 30348451

[26] Michopoulos I, Furukawa TA, Noma H, Kishimoto S, Onishi A, Ostinelli EG, et al. Different control conditions can produce different effect estimates in psychotherapy trials for depression. J Clin Epidemiol. 2021;132:59–70. doi: 10.1016/j.jclinepi.2020.12.012 33338564

[27] Mohr DC, Ho J, Hart TL, Baron KG, Berendsen M, Beckner V, et al. Control condition design and implementation features in controlled trials: A meta-analysis of trials evaluating psychotherapy for depression. Transl Behav Med. 2014;4(4):407–23. doi: 10.1007/s13142-014-0262-3 25584090 PMC4286544

[28] Organization WH. World mental health report: Transforming mental health for all. World Health Organization. 2022.

[29] Page MJ, McKenzie JE, Bossuyt PM, Boutron I, Hoffmann TC, Mulrow CD, et al. The PRISMA 2020 statement: An updated guideline for reporting systematic reviews. Int J Surg. 2021;88:105906. doi: 10.1016/j.ijsu.2021.105906 33789826

[30] Higgins JPT, Thomas J, Chandler J, Cumpston M, Li T, Page MJ, et al. Cochrane Handbook for Systematic Reviews of Interventions. 6.5 ed. Cochrane; 2024.

[31] Sterne JAC, Savović J, Page MJ, Elbers RG, Blencowe NS, Boutron I, et al. RoB 2: A revised tool for assessing risk of bias in randomised trials. BMJ. 2019;366:l4898. doi: 10.1136/bmj.l4898 31462531

[32] McGuinness LA, Higgins JPT. Risk-of-bias VISualization (robvis): An R package and Shiny web app for visualizing risk-of-bias assessments. Res Synth Methods. 2021;12(1):55–61. doi: 10.1002/jrsm.1411 32336025

[33] Borenstein M. Comprehensive meta‐analysis software. Systematic reviews in health research: meta‐analysis in context. 2022. p. 535–48.

[34] Bathe C, Fennen L, Heering T, Greif A, Dubbeldam R. Training interventions to reduce the risk of injury to the lower extremity joints during landing movements in adult athletes: A systematic review and meta-analysis. BMJ Open Sport Exerc Med. 2023;9(2):e001508. doi: 10.1136/bmjsem-2022-001508 37304892 PMC10254820

[35] Cuijpers P, Noma H, Karyotaki E, Vinkers CH, Cipriani A, Furukawa TA. A network meta-analysis of the effects of psychotherapies, pharmacotherapies and their combination in the treatment of adult depression. World Psychiatry. 2020;19(1):92–107. doi: 10.1002/wps.20701 31922679 PMC6953550

[36] Karkou V, Aithal S, Zubala A, Meekums B. Effectiveness of dance movement therapy in the treatment of adults with depression: A systematic review with meta-analyses. Front Psychol. 2019;10:936. doi: 10.3389/fpsyg.2019.00936 31130889 PMC6509172

[37] Viechtbauer W. Conducting Meta-Analyses in R with the metafor Package. J Stat Soft. 2010;36(3). doi: 10.18637/jss.v036.i03

[38] Team R. C. R A language and environment for statistical computing. R Foundation for Statistical Computing; 2020.

[39] Page MJ, Higgins JP, Sterne JA. Chapter 13: Assessing risk of bias due to missing evidence in a meta-analysis. Cochrane handbook for systematic reviews of interventions. 2024.

[40] Sterne JAC, Sutton AJ, Ioannidis JPA, Terrin N, Jones DR, Lau J, et al. Recommendations for examining and interpreting funnel plot asymmetry in meta-analyses of randomised controlled trials. BMJ. 2011;343:d4002. doi: 10.1136/bmj.d4002 21784880

[41] Zwetsloot P-P, Van Der Naald M, Sena ES, Howells DW, IntHout J, De Groot JA, et al. Standardized mean differences cause funnel plot distortion in publication bias assessments. Elife. 2017;6:e24260. doi: 10.7554/eLife.24260 28884685 PMC5621838

[42] Deeks JJ, Higgins JP, Altman DG, Group CSM. Analysing data and undertaking meta‐analyses. Cochrane handbook for systematic reviews of interventions. 2019. p. 241–84.

[43] McKenzie JE, Brennan SE, Ryan RE, Thomson HJ, Johnston RV, Thomas J. Defining the criteria for including studies and how they will be grouped for the synthesis. Cochrane handbook for systematic reviews of interventions. 2019. p. 33–65.

[44] Thompson SG, Higgins JP. How should meta‐regression analyses be undertaken and interpreted?. Statistics in Medicine. 2002;21(11):1559–73. 10.1002/sim.1187 12111920

[45] Borenstein M, Higgins JPT, Hedges LV, Rothstein HR. Basics of meta-analysis: I2 is not an absolute measure of heterogeneity. Res Synth Methods. 2017;8(1):5–18. doi: 10.1002/jrsm.1230 28058794

[46] Cohen J. Statistical power analysis for the behavioral sciences. Academic Press; 2013.

[47] Pagnini F, Phillips D, Haulman A, Bankert M, Simmons Z, Langer E. An online non-meditative mindfulness intervention for people with ALS and their caregivers: A randomized controlled trial. Amyotroph Lateral Scler Frontotemporal Degener. 2022;23(1–2):116–27. doi: 10.1080/21678421.2021.1928707 34027769

[48] Sengupta A, Wagani R. Mindful self-compassion for psychological distress associated with skin conditions: An online intervention study. Indian J Dermatol Venereol Leprol. 2025;91(1):71–5. doi: 10.25259/IJDVL_451_2023 38314971

[49] Higgins JPT, Thompson SG, Spiegelhalter DJ. A re-evaluation of random-effects meta-analysis. J R Stat Soc Ser A Stat Soc. 2009;172(1):137–59. doi: 10.1111/j.1467-985X.2008.00552.x 19381330 PMC2667312

[50] IntHout J, Ioannidis JPA, Rovers MM, Goeman JJ. Plea for routinely presenting prediction intervals in meta-analysis. BMJ Open. 2016;6(7):e010247. doi: 10.1136/bmjopen-2015-010247 27406637 PMC4947751

[51] Riley RD, Higgins JPT, Deeks JJ. Interpretation of random effects meta-analyses. BMJ. 2011;342:d549. doi: 10.1136/bmj.d549 21310794

[52] Groot J, MacLellan A, Butler M, Todor E, Zulfiqar M, Thackrah T, et al. The effectiveness of fully automated digital interventions in promoting mental well-being in the general population: Systematic review and meta-analysis. JMIR Ment Health. 2023;10:e44658. doi: 10.2196/44658 37856172 PMC10623223

[53] Cuijpers P, Miguel C, Harrer M, Ciharova M, Karyotaki E. The overestimation of the effect sizes of psychotherapies for depression in waitlist controlled trials: A meta-analytic comparison with usual care controlled trials. Epidemiol Psychiatr Sci. 2024;33:e56. doi: 10.1017/S2045796024000611 39501660 PMC11561681

[54] Han A, Kim TH. Efficacy of internet-based acceptance and commitment therapy for depressive symptoms, anxiety, stress, psychological distress, and quality of life: Systematic review and meta-analysis. J Med Internet Res. 2022;24(12):e39727. doi: 10.2196/39727 36485030 PMC9789494

[55] Stockton D, Kellett S, Berrios R, Sirois F, Wilkinson N, Miles G. Identifying the underlying mechanisms of change during acceptance and commitment therapy (ACT): A systematic review of contemporary mediation studies. Behav Cogn Psychother. 2019;47(3):332–62. doi: 10.1017/S1352465818000553 30284528

[56] Thompson EM, Destree L, Albertella L, Fontenelle LF. Internet-based acceptance and commitment therapy: A transdiagnostic systematic review and meta-analysis for mental health outcomes. Behav Ther. 2021;52(2):492–507. doi: 10.1016/j.beth.2020.07.002 33622516

[57] Mohr DC, Cuijpers P, Lehman K. Supportive accountability: A model for providing human support to enhance adherence to eHealth interventions. J Med Internet Res. 2011;13(1):e30. doi: 10.2196/jmir.1602 21393123 PMC3221353

[58] Mutter A, Bücheler M, Idrees AR, Domhardt M, Baumeister H. Persuasive design principle of social support in digital interventions targeting mental health symptoms: A systematic review and meta-analysis. BMJ Open. 2024;14(11):e086728. doi: 10.1136/bmjopen-2024-086728 39521463 PMC11552557

[59] Valentine L, Hinton JDX, Bajaj K, Boyd L, O’Sullivan S, Sorenson RP, et al. A meta-analysis of persuasive design, engagement, and efficacy in 92 RCTs of mental health apps. NPJ Digit Med. 2025;8(1):229. doi: 10.1038/s41746-025-01567-5 40301581 PMC12041226

[60] Dindo L, Van Liew JR, Arch JJ. Acceptance and commitment therapy: A transdiagnostic behavioral intervention for mental health and medical conditions. Neurotherapeutics. 2017;14(3):546–53. doi: 10.1007/s13311-017-0521-3 28271287 PMC5509623

[61] Eysenbach G. The law of attrition. J Med Internet Res. 2005;7(1):e11. doi: 10.2196/jmir.7.1.e11 15829473 PMC1550631

