# Supplementary material for: Impacts of digital third-wave interventions for adults on wellbeing and mental health outcomes: A systematic review and meta-analysis of randomized controlled trials
Source: PLOS Digit Health. 2026 Oct 1;5(10):e0001764. doi: 10.1371/journal.pdig.0001764 (PMC13630215; doi:10.1371/journal.pdig.0001764)
Supplement: Supplementary file 2 [file pdig.0001764.s002.docx]

**S2 Appendix. Search strategies.**

Text A.

The search will include studies published from database inception to January 2026. No restrictions will be placed on publication status. A combination of MeSH terms and keywords will be used across all databases. In addition, the following groups of search terms will be employed:

PubMed:

( ("mental health"[All Fields] OR "mental well-being"[All Fields] OR "mental wellbeing"[All Fields] OR "psychological well-being"[All Fields] OR "psychological wellbeing"[All Fields] OR "psychological distress"[All Fields] OR "emotional distress"[All Fields] OR "mood disorder*"[All Fields] OR "mood instability"[All Fields] OR "depressive symptoms"[All Fields] OR "depressive disorder*"[All Fields] OR depression[MeSH Terms] OR "depression"[All Fields] OR "anxiety symptoms"[All Fields] OR "anxiety disorder*"[All Fields] OR anxiety[MeSH Terms] OR "anxiety"[All Fields] OR "stress"[All Fields] OR "chronic stress"[All Fields] OR "perceived stress"[All Fields] OR "psychological resilience"[All Fields] OR "psychological vulnerability"[All Fields] OR "psychopathology"[All Fields] OR "psychosocial outcomes"[All Fields] OR "psychosocial functioning"[All Fields] OR "emotional dysregulation"[All Fields] OR "emotional regulation"[All Fields] OR "emotional well-being"[All Fields] OR "emotional wellbeing"[All Fields] OR "quality of life"[All Fields] OR "QoL"[All Fields] OR "life satisfaction"[All Fields] OR "satisfaction with life"[All Fields] OR "self-efficacy"[All Fields] OR "mental disorder*"[All Fields] OR "psychological symptom*"[All Fields] OR "affective disorder*"[All Fields] OR "emotional instability"[All Fields] OR "subclinical depress*"[All Fields] OR "subthreshold depress*"[All Fields] OR "subclinical anxiety"[All Fields] OR "subthreshold anxiety"[All Fields] OR "psychological flexibility"[All Fields] OR "acceptance and action"[All Fields] OR AAQ[All Fields] OR "depression scale"[All Fields] OR "anxiety scale"[All Fields] OR "stress scale"[All Fields] OR "psychometric outcomes"[All Fields] OR "mental health outcomes"[All Fields] OR "treatment response"[All Fields] OR "treatment outcome"[All Fields] OR "emotional stability"[All Fields] OR "social functioning"[All Fields] OR "life satisfaction scores"[All Fields] OR "self-report measures of well being"[All Fields] OR "self-report measures of well-being"[All Fields] OR "distress tolerance"[All Fields] OR "mindfulness skills"[All Fields] OR decentering[All Fields] OR "cognitive defusion"[All Fields] OR "eudaimonic wellbeing"[All Fields] OR "hedonic wellbeing"[All Fields] OR "subjective wellbeing"[All Fields] OR "positive affect"[All Fields] ) AND ( "third-wave therap*"[All Fields] OR "third wave CBT"[All Fields] OR "third-wave CBT"[All Fields] OR "3rd wave therapies"[All Fields] OR "mindfulness-based cognitive therapy"[All Fields] OR MBCT[All Fields] OR "compassion-focused therapy"[All Fields] OR "compassion-focussed therapy"[All Fields] OR CFT[All Fields] OR "metacognitive therapy"[All Fields] OR "behavioral activation"[All Fields] OR "behavioural activation"[All Fields] OR "mindfulness-based stress reduction"[All Fields] OR MBSR[All Fields] OR "mindfulness interventions"[All Fields] OR "contextual behavioural therapies"[All Fields] OR "contextual behavioral therapies"[All Fields] OR "acceptance-based intervention*"[All Fields] OR "process-based CBT"[All Fields] OR "process-based therapies"[All Fields] OR "mindful self-compassion"[All Fields] OR "self-compassion training"[All Fields] OR "positive psychotherapy"[All Fields] OR "mindfulness based positive psychology"[All Fields] OR "Mindfulness based strength practice"[All Fields] OR "values-based therap*"[All Fields] OR "Values based positive psychology"[All Fields] OR "radical acceptance"[All Fields] OR "distress tolerance training"[All Fields] OR "mindful self-compassion program*"[All Fields] OR "third-wave transdiagnostic therap*"[All Fields] OR "ACT"[All Fields] OR "dialectical behavior therapy"[All Fields] OR "dialectical behaviour therapy"[All Fields] OR "DBT"[All Fields] OR "contextual CBT"[All Fields] OR "Compassion focused positive psychology"[All Fields] OR "Self compassion positive psychology"[All Fields] OR "Acceptance and Commitment Therapy"[MeSH Terms] OR "Dialectical Behavior Therapy"[MeSH Terms] ) AND ( digital[All Fields] OR online[All Fields] OR eHealth[All Fields] OR "e-health"[All Fields] OR mHealth[All Fields] OR "m-health"[All Fields] OR "mobile health"[All Fields] OR telehealth[All Fields] OR teletherapy[All Fields] OR telepsychology[All Fields] OR telepsychiatry[All Fields] OR telemental health[All Fields] OR "web-based"[All Fields] OR "internet-based"[All Fields] OR "technology-mediated interventions"[All Fields] OR "mobile app"[All Fields] OR "smartphone intervention"[All Fields] OR "virtual therapy"[All Fields] OR "virtual intervention"[All Fields] OR VR[All Fields] OR "tele-counselling"[All Fields] OR "tele-counseling"[All Fields] OR "remote therapy"[All Fields] OR "remote mental health delivery"[All Fields] OR "digital behavior change interventions"[All Fields] OR "digital behaviour change interventions"[All Fields] OR "AI-driven therap*"[All Fields] OR computer-based[All Fields] OR "computer-based therapy"[All Fields] OR "internet delivered"[All Fields] OR "digital intervention*"[All Fields] OR "e mental health"[All Fields] OR "e-mental health"[All Fields] OR "electronic mental health"[All Fields] OR "digital mental health tools"[All Fields] OR "app-based therap*"[All Fields] OR "digital self-help"[All Fields] OR "guided internet intervention*"[All Fields] OR "automated therap*"[All Fields] OR "chatbot intervention*"[All Fields] ) AND ( "randomised controlled trial"[All Fields] OR "randomized controlled trial"[All Fields] OR "randomised controlled trial"[Publication Type] OR "randomized controlled trial"[Publication Type] OR "controlled clinical trial"[Publication Type] OR "clinical trial"[Publication Type] OR "randomised study"[All Fields] OR "randomized study"[All Fields] OR RCT[All Fields] OR "controlled clinical trial"[All Fields] OR "clinical trial"[All Fields] OR "systematic review"[All Fields] OR "cross-over trial"[All Fields] OR "crossover trial"[All Fields] OR "sham-controlled"[All Fields] OR "random allocation"[All Fields] OR "randomly assigned"[All Fields] OR "double-blind procedure"[All Fields] OR "waitlist control"[All Fields] OR "randomised experiment"[All Fields] OR "randomized experiment"[All Fields] ) )

PsycINFO and PsycArticles (Accessed through EBSCOhost):

( ( TI(( "mental health" OR "mental well-being" OR "mental wellbeing" OR "psychological well-being" OR "psychological wellbeing" OR "psychological distress" OR "emotional distress" OR mood disorder* OR "mood instability" OR "depressive symptoms" OR depressive disorder* OR depression OR "anxiety symptoms" OR anxiety disorder* OR anxiety OR stress OR "chronic stress" OR "perceived stress" OR "psychological resilience" OR "psychological vulnerability" OR psychopathology OR "psychosocial outcomes" OR "psychosocial functioning" OR "emotional dysregulation" OR "emotional regulation" OR "emotional well-being" OR "emotional wellbeing" OR "quality of life" OR QoL OR "life satisfaction" OR "satisfaction with life" OR self-efficacy OR mental disorder* OR psychological symptom* OR affective disorder* OR "emotional instability" OR subclinical depress* OR subthreshold depress* OR "subclinical anxiety" OR "subthreshold anxiety" OR "psychological flexibility" OR "acceptance and action" OR AAQ OR "depression scale" OR "anxiety scale" OR "stress scale" OR "psychometric outcomes" OR "mental health outcomes" OR "treatment response" OR "treatment outcome" OR "emotional stability" OR "social functioning" OR "life satisfaction scores" OR "self-report measures of well being" OR "self-report measures of well-being" OR "distress tolerance" OR "mindfulness skills" OR decentering OR "cognitive defusion" OR "eudaimonic wellbeing" OR "hedonic wellbeing" OR "subjective wellbeing" OR "positive affect" )) OR AB(( "mental health" OR "mental well-being" OR "mental wellbeing" OR "psychological well-being" OR "psychological wellbeing" OR "psychological distress" OR "emotional distress" OR mood disorder* OR "mood instability" OR "depressive symptoms" OR depressive disorder* OR depression OR "anxiety symptoms" OR anxiety disorder* OR anxiety OR stress OR "chronic stress" OR "perceived stress" OR "psychological resilience" OR "psychological vulnerability" OR psychopathology OR "psychosocial outcomes" OR "psychosocial functioning" OR "emotional dysregulation" OR "emotional regulation" OR "emotional well-being" OR "emotional wellbeing" OR "quality of life" OR QoL OR "life satisfaction" OR "satisfaction with life" OR self-efficacy OR mental disorder* OR psychological symptom* OR affective disorder* OR "emotional instability" OR subclinical depress* OR subthreshold depress* OR "subclinical anxiety" OR "subthreshold anxiety" OR "psychological flexibility" OR "acceptance and action" OR AAQ OR "depression scale" OR "anxiety scale" OR "stress scale" OR "psychometric outcomes" OR "mental health outcomes" OR "treatment response" OR "treatment outcome" OR "emotional stability" OR "social functioning" OR "life satisfaction scores" OR "self-report measures of well being" OR "self-report measures of well-being" OR "distress tolerance" OR "mindfulness skills" OR decentering OR "cognitive defusion" OR "eudaimonic wellbeing" OR "hedonic wellbeing" OR "subjective wellbeing" OR "positive affect" )) ) AND ( ( TI(( "third-wave therap*" OR "third wave CBT" OR "third-wave CBT" OR "3rd wave therapies" OR "mindfulness-based cognitive therapy" OR MBCT OR "compassion-focused therapy" OR "compassion-focussed therapy" OR CFT OR "metacognitive therapy" OR "behavioral activation" OR "behavioural activation" OR "mindfulness-based stress reduction" OR MBSR OR "mindfulness interventions" OR "contextual behavioural therapies" OR "contextual behavioral therapies" OR "acceptance-based intervention*" OR "process-based CBT" OR "process-based therapies" OR "mindful self-compassion" OR "self-compassion training" OR "positive psychotherapy" OR "mindfulness based positive psychology" OR "Mindfulness based strength practice" OR "values-based therap*" OR "Values based positive psychology" OR "radical acceptance" OR "distress tolerance training" OR "mindful self-compassion program*" OR "third-wave transdiagnostic therap*" OR ACT OR "dialectical behavior therapy" OR "dialectical behaviour therapy" OR DBT OR "contextual CBT" OR "Compassion focused positive psychology" OR "Self compassion positive psychology" )) OR AB(( "third-wave therap*" OR "third wave CBT" OR "third-wave CBT" OR "3rd wave therapies" OR "mindfulness-based cognitive therapy" OR MBCT OR "compassion-focused therapy" OR "compassion-focussed therapy" OR CFT OR "metacognitive therapy" OR "behavioral activation" OR "behavioural activation" OR "mindfulness-based stress reduction" OR MBSR OR "mindfulness interventions" OR "contextual behavioural therapies" OR "contextual behavioral therapies" OR "acceptance-based intervention*" OR "process-based CBT" OR "process-based therapies" OR "mindful self-compassion" OR "self-compassion training" OR "positive psychotherapy" OR "mindfulness based positive psychology" OR "Mindfulness based strength practice" OR "values-based therap*" OR "Values based positive psychology" OR "radical acceptance" OR "distress tolerance training" OR "mindful self-compassion program*" OR "third-wave transdiagnostic therap*" OR ACT OR "dialectical behavior therapy" OR "dialectical behaviour therapy" OR DBT OR "contextual CBT" OR "Compassion focused positive psychology" OR "Self compassion positive psychology" )) ) AND ( ( TI(( digital OR online OR eHealth OR "e-health" OR mHealth OR "m-health" OR "mobile health" OR telehealth OR teletherapy OR telepsychology OR telepsychiatry OR telemental health OR "web-based" OR "internet-based" OR "technology-mediated interventions" OR "mobile app" OR "smartphone intervention" OR "virtual therapy" OR "virtual intervention" OR VR OR "tele-counselling" OR "tele-counseling" OR "remote therapy" OR "remote mental health delivery" OR "digital behavior change interventions" OR "digital behaviour change interventions" OR AI-driven therap* OR computer-based OR "computer-based therapy" OR "internet delivered" OR "digital intervention*" OR "e mental health" OR "e-mental health" OR "electronic mental health" OR "digital mental health tools" OR "app-based therap*" OR "digital self-help" OR "guided internet intervention*" OR "automated therap*" OR "chatbot intervention*" )) OR AB(( digital OR online OR eHealth OR "e-health" OR mHealth OR "m-health" OR "mobile health" OR telehealth OR teletherapy OR telepsychology OR telepsychiatry OR telemental health OR "web-based" OR "internet-based" OR "technology-mediated interventions" OR "mobile app" OR "smartphone intervention" OR "virtual therapy" OR "virtual intervention" OR VR OR "tele-counselling" OR "tele-counseling" OR "remote therapy" OR "remote mental health delivery" OR "digital behavior change interventions" OR "digital behaviour change interventions" OR AI-driven therap* OR computer-based OR "computer-based therapy" OR "internet delivered" OR "digital intervention*" OR "e mental health" OR "e-mental health" OR "electronic mental health" OR "digital mental health tools" OR "app-based therap*" OR "digital self-help" OR "guided internet intervention*" OR "automated therap*" OR "chatbot intervention*" )) ) AND ( ( TI(( "randomised controlled trial" OR "randomized controlled trial" OR RCT OR "randomised study" OR "randomized study" OR "controlled clinical trial" OR "clinical trial" OR "systematic review" OR "cross-over trial" OR "crossover trial" OR "sham-controlled" OR "random allocation" OR "randomly assigned" OR "double-blind procedure" OR "waitlist control" OR "randomised experiment" OR "randomized experiment" )) OR AB(( "randomised controlled trial" OR "randomized controlled trial" OR RCT OR "randomised study" OR "randomized study" OR "controlled clinical trial" OR "clinical trial" OR "systematic review" OR "cross-over trial" OR "crossover trial" OR "sham-controlled" OR "random allocation" OR "randomly assigned" OR "double-blind procedure" OR "waitlist control" OR "randomised experiment" OR "randomized experiment" )) )

Cochrane Library:

( ("mental health" OR "mental well-being" OR "mental wellbeing" OR "psychological well-being" OR "psychological wellbeing" OR "psychological distress" OR "emotional distress" OR "mood disorder*" OR "mood instability" OR "depressive symptoms" OR "depressive disorder*" OR depression OR "anxiety symptoms" OR "anxiety disorder*" OR anxiety OR stress OR "chronic stress" OR "perceived stress" OR "psychological resilience" OR "psychological vulnerability" OR psychopathology OR "psychosocial outcomes" OR "psychosocial functioning" OR "emotional dysregulation" OR "emotional regulation" OR "emotional well-being" OR "emotional wellbeing" OR "quality of life" OR QoL OR "life satisfaction" OR "satisfaction with life" OR "self-efficacy" OR "mental disorder*" OR "psychological symptom*" OR "affective disorder*" OR "emotional instability" OR "subclinical depress*" OR "subthreshold depress*" OR "subclinical anxiety" OR "subthreshold anxiety" OR "psychological flexibility" OR "acceptance and action" OR AAQ OR "depression scale" OR "anxiety scale" OR "stress scale" OR "psychometric outcomes" OR "mental health outcomes" OR "treatment response" OR "treatment outcome" OR "emotional stability" OR "social functioning" OR "life satisfaction scores" OR "self-report measures of well being" OR "self-report measures of well-being" OR "distress tolerance" OR "mindfulness skills" OR decentering OR "cognitive defusion" OR "eudaimonic wellbeing" OR "hedonic wellbeing" OR "subjective wellbeing" OR "positive affect")) AND ( ("third-wave therap*" OR "third wave CBT" OR "third-wave CBT" OR "3rd wave therapies" OR "mindfulness-based cognitive therapy" OR MBCT OR "compassion-focused therapy" OR "compassion-focussed therapy" OR CFT OR "metacognitive therapy" OR "behavioural activation" OR "mindfulness-based stress reduction" OR MBSR OR "mindfulness interventions" OR "contextual behavioural therapies" OR "contextual behavioural therapies" OR "contextual behavioral therapies" OR "acceptance-based intervention*" OR "process-based CBT" OR "process-based therapies" OR "mindful self-compassion" OR "self-compassion training" OR "positive psychotherapy" OR "mindfulness based positive psychology" OR "values-based therap*" OR "radical acceptance" OR "distress tolerance training" OR "mindful self-compassion program*" OR "third-wave transdiagnostic therap*" OR "ACT" OR "dialectical behavior therapy" OR "dialectical behaviour therapy" OR DBT OR "contextual CBT" OR "Compassion focused positive psychology" OR "Self compassion positive psychology")) AND (digital OR online OR eHealth OR "e-health" OR mHealth OR "m-health" OR "mobile health" OR telehealth OR teletherapy OR telepsychology OR telepsychiatry OR telemental health OR "web-based" OR "internet-based" OR "technology-mediated interventions" OR "mobile app" OR "smartphone intervention" OR "virtual therapy" OR "virtual intervention" OR VR OR "tele-counselling" OR "tele-counseling" OR "remote therapy" OR "remote mental health delivery" OR "digital behavior change interventions" OR "digital behaviour change interventions" OR "AI-driven therap*" OR "computer-based" OR "computer-based therapy" OR "internet delivered" OR "digital intervention*" OR "e mental health" OR "e-mental health" OR "electronic mental health" OR "digital mental health tools" OR "app-based therap*" OR "digital self-help" OR "guided internet intervention*" OR "automated therap*" OR "chatbot intervention*") AND ("randomised controlled trial" OR "randomized controlled trial" OR RCT OR "randomised study" OR "randomized study" OR "controlled clinical trial" OR "clinical trial" OR "systematic review" OR "cross-over trial" OR "crossover trial" OR "sham-controlled" OR "random allocation" OR "randomly assigned" OR "double-blind procedure" OR "waitlist control" OR "randomised experiment" OR "randomized experiment")

Scopus:

ALL( "mental health" OR "mental well-being" OR "mental wellbeing" OR "psychological well-being" OR "psychological wellbeing" OR "psychological distress" OR "emotional distress" OR "mood disorder*" OR "mood instability" OR "depressive symptoms" OR "depressive disorder*" OR depression OR "anxiety symptoms" OR "anxiety disorder*" OR anxiety OR stress OR "chronic stress" OR "perceived stress" OR "psychological resilience" OR "psychological vulnerability" OR psychopathology OR "psychosocial outcomes" OR "psychosocial functioning" OR "emotional dysregulation" OR "emotional regulation" OR "emotional well-being" OR "emotional wellbeing" OR "quality of life" OR QoL OR "life satisfaction" OR "satisfaction with life" OR "self-efficacy" OR "mental disorder*" OR "psychological symptom*" OR "affective disorder*" OR "emotional instability" OR "subclinical depress*" OR "subthreshold depress*" OR "subclinical anxiety" OR "subthreshold anxiety" OR "psychological flexibility" OR "acceptance and action" OR AAQ OR "depression scale" OR "anxiety scale" OR "stress scale" OR "psychometric outcomes" OR "mental health outcomes" OR "treatment response" OR "treatment outcome" OR "emotional stability" OR "social functioning" OR "life satisfaction scores" OR "self-report measures of well being" OR "self-report measures of well-being" OR "distress tolerance" OR "mindfulness skills" OR decentering OR "cognitive defusion" OR "eudaimonic wellbeing" OR "hedonic wellbeing" OR "subjective wellbeing" OR "positive affect" ) AND TITLE-ABS-KEY( "third-wave therap*" OR "third wave CBT" OR "third-wave CBT" OR "3rd wave therapies" OR "mindfulness-based cognitive therapy" OR MBCT OR "compassion-focused therapy" OR "compassion-focussed therapy" OR CFT OR "metacognitive therapy" OR "behavioural activation" OR "behavioral activation" OR "mindfulness-based stress reduction" OR MBSR OR "mindfulness interventions" OR "contextual behavioural therapies" OR "contextual behavioral therapies" OR "acceptance-based intervention*" OR "process-based CBT" OR "process-based therapies" OR "mindful self-compassion" OR "self-compassion training" OR "positive psychotherapy" OR "mindfulness based positive psychology" OR "values-based therap*" OR "Values based positive psychology" OR "radical acceptance" OR "distress tolerance training" OR "mindful self-compassion program*" OR "third-wave transdiagnostic therap*" OR "Acceptance and Commitment Therapy" OR "Dialectical Behavior Therapy" OR "Dialectical Behaviour Therapy" OR DBT OR "contextual CBT" OR "Compassion focused positive psychology" OR "Self compassion positive psychology" OR ACT ) AND TITLE-ABS-KEY( digital OR online OR eHealth OR "e-health" OR mHealth OR "m-health" OR "mobile health" OR telehealth OR teletherapy OR telepsychology OR telepsychiatry OR telemental health OR "web-based" OR "internet-based" OR "technology-mediated interventions" OR "mobile app" OR "smartphone intervention" OR "virtual therapy" OR "virtual intervention" OR VR OR "tele-counselling" OR "tele-counseling" OR "remote therapy" OR "remote mental health delivery" OR "digital behavior change interventions" OR "digital behaviour change interventions" OR "AI-driven therap*" OR "computer-based" OR "computer-based therapy" OR "internet delivered" OR "digital intervention*" OR "e mental health" OR "electronic mental health" ) AND TITLE-ABS-KEY( "randomised controlled trial" OR "randomized controlled trial" OR RCT OR "randomised study" OR "randomized study" OR "controlled clinical trial" OR "clinical trial" OR "systematic review" OR "cross-over trial" OR "crossover trial" OR "sham-controlled" OR "random allocation" OR "randomly assigned" OR "double-blind procedure" OR "waitlist control" )

EMBASE:

('mental health'/exp OR 'mental health' OR 'mental well-being'/exp OR 'mental well-being' OR 'mental wellbeing'/exp OR 'mental wellbeing' OR 'psychological well-being'/exp OR 'psychological well-being' OR 'psychological wellbeing'/exp OR 'psychological wellbeing' OR 'psychological distress'/exp OR 'psychological distress' OR 'emotional distress'/exp OR 'emotional distress' OR 'mood disorder*' OR 'mood instability'/exp OR 'mood instability' OR 'depressive symptoms'/exp OR 'depressive symptoms' OR 'depressive disorder*' OR 'depression'/exp OR depression OR 'anxiety symptoms' OR 'anxiety disorder*' OR 'anxiety'/exp OR anxiety OR 'stress'/exp OR stress OR 'chronic stress'/exp OR 'chronic stress' OR 'perceived stress'/exp OR 'perceived stress' OR 'psychological resilience'/exp OR 'psychological resilience' OR 'psychological vulnerability'/exp OR 'psychological vulnerability' OR 'psychopathology'/exp OR psychopathology OR 'psychosocial outcomes' OR 'psychosocial functioning'/exp OR 'psychosocial functioning' OR 'emotional dysregulation'/exp OR 'emotional dysregulation' OR 'emotional regulation'/exp OR 'emotional regulation' OR 'emotional well-being'/exp OR 'emotional well-being' OR 'emotional wellbeing'/exp OR 'emotional wellbeing' OR 'quality of life'/exp OR 'quality of life' OR qol OR 'life satisfaction'/exp OR 'life satisfaction' OR 'satisfaction with life' OR 'self-efficacy'/exp OR 'self-efficacy' OR 'mental disorder*' OR 'psychological symptom*' OR 'affective disorder*' OR 'emotional instability'/exp OR 'emotional instability' OR 'subclinical depress*' OR 'subthreshold depress*' OR 'subclinical anxiety' OR 'subthreshold anxiety' OR 'psychological flexibility'/exp OR 'psychological flexibility' OR 'acceptance and action' OR aaq OR 'depression scale'/exp OR 'depression scale' OR 'anxiety scale'/exp OR 'anxiety scale' OR 'stress scale'/exp OR 'stress scale' OR 'psychometric outcomes' OR 'mental health outcomes' OR 'treatment response'/exp OR 'treatment response' OR 'treatment outcome'/exp OR 'treatment outcome' OR 'emotional stability'/exp OR 'emotional stability' OR 'social functioning'/exp OR 'social functioning' OR 'life satisfaction scores' OR 'self-report measures of well being' OR 'distress tolerance'/exp OR 'distress tolerance' OR 'mindfulness skills' OR 'decentering'/exp OR decentering OR 'cognitive defusion'/exp OR 'cognitive defusion' OR 'eudaimonic wellbeing'/exp OR 'eudaimonic wellbeing' OR 'hedonic wellbeing'/exp OR 'hedonic wellbeing' OR 'subjective wellbeing'/exp OR 'subjective wellbeing' OR 'positive affect'/exp OR 'positive affect') AND (('third-wave therap*':ti,ab,kw OR 'third wave cbt':ti,ab,kw OR 'third-wave cbt':ti,ab,kw OR '3rd wave therapies':ti,ab,kw OR 'mindfulness-based cognitive therapy':ti,ab,kw OR mbct:ti,ab,kw OR 'compassion-focused therapy':ti,ab,kw OR 'compassion-focussed therapy':ti,ab,kw OR cft:ti,ab,kw OR 'metacognitive therapy':ti,ab,kw OR 'behavioural activation':ti,ab,kw OR 'behavioral activation':ti,ab,kw OR 'mindfulness-based stress reduction':ti,ab,kw OR mbsr:ti,ab,kw OR 'mindfulness interventions':ti,ab,kw OR 'contextual behavioural therapies':ti,ab,kw OR 'contextual behavioral therapies':ti,ab,kw OR 'acceptance-based intervention*':ti,ab,kw OR 'process-based cbt':ti,ab,kw OR 'process-based therapies':ti,ab,kw OR 'mindful self-compassion':ti,ab,kw OR 'self-compassion training':ti,ab,kw OR 'positive psychotherapy':ti,ab,kw OR 'mindfulness based positive psychology':ti,ab,kw OR 'values-based therap*':ti,ab,kw OR 'values based positive psychology':ti,ab,kw OR 'radical acceptance':ti,ab,kw OR 'distress tolerance training':ti,ab,kw OR 'mindful self-compassion program*':ti,ab,kw OR 'third-wave transdiagnostic therap*':ti,ab,kw OR acceptance:ti,ab,kw) AND 'commitment therapy':ti,ab,kw OR 'dialectical behavior therapy':ti,ab,kw OR 'dialectical behaviour therapy':ti,ab,kw OR dbt:ti,ab,kw OR 'contextual cbt':ti,ab,kw OR 'compassion focused positive psychology':ti,ab,kw OR 'self compassion positive psychology':ti,ab,kw OR act:ti,ab,kw) AND (digital:ti,ab,kw OR online:ti,ab,kw OR ehealth:ti,ab,kw OR 'e-health':ti,ab,kw OR mhealth:ti,ab,kw OR 'm-health':ti,ab,kw OR 'mobile health':ti,ab,kw OR telehealth:ti,ab,kw OR teletherapy:ti,ab,kw OR telepsychology:ti,ab,kw OR telepsychiatry:ti,ab,kw OR 'telemental health':ti,ab,kw OR 'web-based':ti,ab,kw OR 'internet-based':ti,ab,kw OR 'technology-mediated interventions':ti,ab,kw OR 'mobile app':ti,ab,kw OR 'smartphone intervention':ti,ab,kw OR 'virtual therapy':ti,ab,kw OR 'virtual intervention':ti,ab,kw OR vr:ti,ab,kw OR 'tele-counselling':ti,ab,kw OR 'tele-counseling':ti,ab,kw OR 'remote therapy':ti,ab,kw OR 'remote mental health delivery':ti,ab,kw OR 'digital behavior change interventions':ti,ab,kw OR 'digital behaviour change interventions':ti,ab,kw OR 'ai-driven therap*':ti,ab,kw OR 'computer-based':ti,ab,kw OR 'computer-based therapy':ti,ab,kw OR 'internet delivered':ti,ab,kw OR 'digital intervention*':ti,ab,kw OR 'e mental health':ti,ab,kw OR 'electronic mental health':ti,ab,kw) AND ('randomised controlled trial':ti,ab,kw OR 'randomized controlled trial':ti,ab,kw OR rct:ti,ab,kw OR 'randomised study':ti,ab,kw OR 'randomized study':ti,ab,kw OR 'controlled clinical trial':ti,ab,kw OR 'clinical trial':ti,ab,kw OR 'systematic review':ti,ab,kw OR 'cross-over trial':ti,ab,kw OR 'crossover trial':ti,ab,kw OR 'sham-controlled':ti,ab,kw OR 'random allocation':ti,ab,kw OR 'randomly assigned':ti,ab,kw OR 'double-blind procedure':ti,ab,kw OR 'waitlist control':ti,ab,kw)
