# Supplementary material for: Impacts of digital third-wave interventions for adults on wellbeing and mental health outcomes: A systematic review and meta-analysis of randomized controlled trials
Source: PLOS Digit Health. 2026 Oct 1;5(10):e0001764. doi: 10.1371/journal.pdig.0001764 (PMC13630215; doi:10.1371/journal.pdig.0001764)
Supplement: Supplementary file 3 [file pdig.0001764.s003.docx]

**S3 Appendix. Extraction sheet.**

Table A. Data extraction form.

| Category | Data Field | Description |
| --- | --- | --- |
| Study Identification | Study ID | Unique study identifier: first author + year (e.g., Smith 2023). Use consistently across all rows for that trial. |
| Study Identification | DOI | Digital Object Identifier for the primary trial report (leave blank if not available). |
| Intervention | Intervention Type | Intervention family/modality code (e.g., ACT, MBCT, MBSR, Mindfulness-based, DBT, CFT). Use your predefined coding rules. |
| Population | Population Type | Categorised as: Healthy, Mental health condition, or Medical condition (match your protocol definitions exactly). |
| Comparator | Comparator Type | Categorised as: Active, Inactive, or Multi-arm (use Multi-arm when the trial includes more than one eligible comparator arm in the extraction). |
| Intervention | Therapist Involvement | Guidance level for the extracted arm: Guided or Unguided. Use “Control” only for control/comparator arms (so guidance analyses can exclude/handle these cleanly). |
| Outcomes | Specific Outcome | Outcome domain for the row: Wellbeing, Depression, Anxiety, Stress, Psychological flexibility, Quality of life. |
| Outcomes | Outcome Measure Tool | Name of the psychometric instrument used for that domain (e.g., PHQ-9, GAD-7, PSS, AAQ-II, WHOQOL-BREF, MHC-SF). |
| Outcomes | Timepoint | Assessment timing: Baseline, Post-intervention, Short-term (≤3 months), Medium-term (>3–6 months), Long-term (>6 months). Use these exact labels. |
| Arm Identification | Group | Verbatim arm label as reported in the paper (e.g., programme name, “care as usual”, “waitlist control”). Keep as close to the study’s wording as possible. |
| Arm Identification | Streamlined Group | Standardised arm grouping used for analysis: Intervention, Active Control, Inactive Control. |
| Statistical Data | n | Sample size for this arm at this timepoint for the extracted measure (use observed n for that timepoint/measure where reported). |
| Statistical Data | Mean | Mean score for this arm at this timepoint on the extracted measure. |
| Statistical Data | SD | Standard deviation for this arm at this timepoint on the extracted measure. |
| Outcome Orientation | Units/Score Direction | Score direction after harmonisation: specify whether “Higher = better” or “Higher = worse” (or equivalent). This should reflect the coding used to orient effects consistently. |
| Intervention Dose | Intervention Duration | Intervention duration in weeks (numeric where possible). If the intervention is session/module-based, encode duration as weeks where available (or leave blank if not reported). |
