# Supplementary material for: Impacts of digital third-wave interventions for adults on wellbeing and mental health outcomes: A systematic review and meta-analysis of randomized controlled trials
Source: PLOS Digit Health. 2026 Oct 1;5(10):e0001764. doi: 10.1371/journal.pdig.0001764 (PMC13630215; doi:10.1371/journal.pdig.0001764)
Supplement: Supplementary file 4 [file pdig.0001764.s004.docx]

**S4 Appendix. Outcome measurement hierarchy.**

Text A.

To avoid unit-of-analysis errors, when studies reported multiple measures for the same outcome construct, a single measure was selected for meta-analysis according to the following hierarchy, as prespecified in the review protocol (OSF DOI: 10.17605/OSF.IO/5RMU2):

**Subjective Wellbeing**

- *Priority 1:* Warwick-Edinburgh Mental Well-being Scale (WEMWBS-14)
- *Priority 2:* Short Warwick-Edinburgh Mental Well-being Scale (WEMWBS-7)
- *Priority 3:* Mental Health Continuum – Short Form (MHC-SF)
- *Priority 4:* WHO-5 Well-being Index
- *Priority 5:* Satisfaction with Life Scale (SWLS)
- *Priority 6:* Ryff Psychological Well-Being Scales

**Depressive Symptoms**

- *Priority 1:* Patient Health Questionnaire-9 (PHQ-9)
- *Priority 2:* Beck Depression Inventory (BDI-II)
- *Priority 3:* Center for Epidemiologic Studies Depression Scale (CES-D)
- *Priority 4:* Hospital Anxiety and Depression Scale – Depression (HADS-D)
- *Priority 5:* DASS-21 Depression Subscale

**Anxiety Symptoms**

- *Priority 1:* Generalised Anxiety Disorder-7 (GAD-7)
- *Priority 2:* State-Trait Anxiety Inventory – State (STAI-S)
- *Priority 3:* Hospital Anxiety and Depression Scale – Anxiety (HADS-A)
- *Priority 4:* DASS-21 Anxiety Subscale
- *Priority 5:* Beck Anxiety Inventory (BAI)

**Perceived Stress**

- *Priority 1:* Perceived Stress Scale (PSS-10)
- *Priority 2:* Perceived Stress Scale (PSS-14)
- *Priority 3:* Kessler Psychological Distress Scale (K-10)
- *Priority 4:* CORE-OM (Total)
- *Priority 5:* General Health Questionnaire (GHQ-12) or Distress Questionnaire-5 (DQ-5)

**Psychological Flexibility**

- *Priority 1:* Comprehensive assessment of Acceptance and Commitment Therapy processes (CompACT)
- *Priority 2:* Acceptance and Action Questionnaire (AAQ-II)
- *Priority 3:* Multidimensional Psychological Flexibility Inventory (MPFI) – Flexibility Subscale
- *Priority 4:* Committed Action Questionnaire (CAQ)

**Quality of Life**

- *Priority 1:* EQ-5D-5L (VAS)
- *Priority 2:* SF-12 Mental Component Score (MCS)
- *Priority 3:* SF-36 Mental Component Score (MCS)
- *Priority 4:* WHOQOL-BREF (Psychological Domain)
