# Supplementary material for: Impacts of digital third-wave interventions for adults on wellbeing and mental health outcomes: A systematic review and meta-analysis of randomized controlled trials
Source: PLOS Digit Health. 2026 Oct 1;5(10):e0001764. doi: 10.1371/journal.pdig.0001764 (PMC13630215; doi:10.1371/journal.pdig.0001764)
Supplement: Supplementary file 6 [file pdig.0001764.s006.docx]

**S6 Appendix. Full reference list for the 147 studies included in this meta-analysis.**

Text A.

1. Acikgoz, F., & Karaca, A. (2025). The effect of a mindfulness-based cognitive therapy program on depression, anxiety, stress, and cognitive flexibility in nursing students: A randomized clinical trial. Journal of Professional Nursing, 56, 94-103. https://doi.org/10.1016/j.profnurs.2024.12.005

2. Adler, A., Sinai, D., & Hamiel, D. (2025). Third-wave CBT online intervention for older adults inspired by ACT principles: An evaluation of feasibility and preliminary effectiveness in adults age 75 and older. Clinical gerontologist, 48(4), 633-643. https://doi.org/10.1080/07317115.2024.2446460

3. Aikman, S. N., Verhaeghen, P., & Ram, N. (2025). Mindfulness from me to we: A randomized control trial on the effects of an 8-week mindfulness intervention on mindfulness, well-being, mental health, compassion, and prosociality. American Journal of Orthopsychiatry. https://doi.org/10.1037/ort0000828

4. Al-Refae, M., Al-Refae, A., Munroe, M., Sardella, N. A., & Ferrari, M. (2021). A self-compassion and mindfulness-based cognitive mobile intervention (Serene) for depression, anxiety, and stress: Promoting adaptive emotional regulation and wisdom. Frontiers in Psychology, 12, 648087. https://doi.org/10.3389/fpsyg.2021.648087

5. Alhawatmeh, H., Abusaif, L., Alharrasi, M., AbuAlrub, R. F., & Hamaideh, S. H. (2025). Holistic healing through mindfulness: An experimental study addressing psychological challenges among nurses caring for patients with COVID-19. Journal of Holistic Nursing, 08980101251323021. https://doi.org/10.1177/08980101251323021

6. Alimohamadi, A., Ghasemzadeh, M., Baberi, F., Rostami, H., Sirois, F., Matin, H., ... & Nikfar, E. (2025). Evaluating the effectiveness of compassion‐focused therapy on psychological outcomes in medical students: A pilot randomized controlled trial. British Journal of Health Psychology, 30(4), e70029. https://doi.org/10.1111/bjhp.70029

7. Alzahrani, A. M., Hakami, A., AlHadi, A., Al-Maflehi, N., Aljawadi, M. H., Alotaibi, R. M., ... & Almigbal, T. H. (2023). The effectiveness of mindfulness training in improving medical students’ stress, depression, and anxiety. Plos one, 18(10), e0293539. https://doi.org/10.1371/journal.pone.0293539

8. Badaghi, N., Kwakkenbos, L., Prins, J., Donders, R., Kelders, S., & Speckens, A. (2025). Randomized Controlled Trial of Group‐Blended and Individual‐Unguided Online Mindfulness‐Based Cognitive Therapy to Reduce Psychological Distress in People With Cancer. Psycho‐Oncology, 34(9), e70286. https://doi.org/10.1002/pon.70286

9. Baminiwatta, A., Fernando, R., Solangaarachchi, I., Abayabandara‐Herath, T., Wickremasinghe, A. R., & Hapangama, A. (2024). Improving psychological well‐being among healthcare workers during the COVID‐19 pandemic with an online mindfulness intervention: A randomised waitlist‐controlled trial. International Journal of Psychology, 59(3), 410-418. https://doi.org/10.1002/ijop.13118

10. Barcaccia, B., Medvedev, O. N., Pallini, S., Mastandrea, S., & Fagioli, S. (2024). Examining mental health benefits of a brief online mindfulness intervention: A randomised controlled trial. Mindfulness, 15(4), 835-843. https://doi.org/10.1007/s12671-024-02331-8

11. Bendelin, N., Gerdle, B., Blom, M., Södermark, M., & Andersson, G. (2021). Internet-delivered acceptance and commitment therapy added to multimodal pain rehabilitation: a cluster randomized controlled trial. Journal of clinical medicine, 10(24), 5872. https://doi.org/10.3390/jcm10245872

12. Bermudo-Gallaguet, A., Ariza, M., Agudelo, D., Camins-Vila, N., Boldó, M., Peters, S., ... & Mataró, M. (2025). Effects of mindfulness and exercise on growth factors, inflammation, and stress markers in chronic stroke: the MindFit project randomized clinical trial. Journal of Clinical Medicine, 14(8), 2580. https://doi.org/10.3390/jcm14082580

13. Beshai, S., Bueno, C., Yu, M., Feeney, J. R., & Pitariu, A. (2020). Examining the effectiveness of an online program to cultivate mindfulness and self-compassion skills (Mind-OP): randomized controlled trial on Amazon's Mechanical Turk. Behaviour Research and Therapy, 134, 103724. https://doi.org/10.1016/j.brat.2020.103724

14. Bindicsova, I., Hides, L. M., Brain, K., Hodson, F. J., Jackson, P., Jensen, M. P., ... & Day, M. A. (2025). Internet-delivered mindfulness-based cognitive therapy (iMBCT) for chronic pain symptom management in adults: A pilot randomized controlled trial. The Journal of Pain, 32, 105429. https://doi.org/10.1016/j.jpain.2025.105429

15. Bruhns, A., Lüdtke, T., Moritz, S., & Bücker, L. (2021). A mobile-based intervention to increase self-esteem in students with depressive symptoms: randomized controlled trial. JMIR mHealth and uHealth, 9(7), e26498. https://doi.org/10.2196/26498

16. Buhrman, M., Skoglund, A., Husell, J., Bergström, K., Gordh, T., Hursti, T., ... & Andersson, G. (2013). Guided internet-delivered acceptance and commitment therapy for chronic pain patients: a randomized controlled trial. Behaviour research and therapy, 51(6), 307-315. https://doi.org/10.1016/j.brat.2013.02.010

17. Buhrman, M., Tillfors, M., Holländare, F., Lekström, E., Håkansson, A., & Boersma, K. (2023). Psychological treatment targeting acceptance and compassion in patients with chronic pain: A randomized controlled, internet-delivered, treatment trial. The Clinical Journal of Pain, 39(12), 672-685. https://doi.org/10.1097/AJP.0000000000001157

18. Buhrman, M., Hällström, H., Fridén, A., Kettis Moden, E., Grahn, G., Carlfjord, M., ... & McCracken, L. M. (2024). Guided internet‐based acceptance and commitment therapy for provoked vestibulodynia: A randomized controlled trial. European Journal of Pain, 28(7), 1185-1201. https://doi.org/10.1002/ejp.2253

19. Bäckman, L., Weineland, S., Vernmark, K., Radvogin, E., Bjälkebring, P., Enbuske, E., ... & Hursti, T. (2025). Internet‐Based Acceptance and Commitment Therapy With Interoceptive Exposure for Panic Disorder: A Randomized Controlled Trial and Working Alliance Analysis. Scandinavian Journal of Psychology. https://doi.org/10.1111/sjop.70045

20. Cao, Q., Du, X., Ruiz, F. J., Yu, X., & Ren, Z. (2025). Efficacy of applet-based acceptance and commitment therapy focused on repetitive negative thinking for adults with comorbid anxiety and depression: A randomized controlled trial with mediation analysis. General Hospital Psychiatry. https://doi.org/10.1016/j.genhosppsych.2025.09.006

21. Carlbring, P., Hägglund, M., Luthström, A., Dahlin, M., Kadowaki, Å., Vernmark, K., & Andersson, G. (2013). Internet-based behavioral activation and acceptance-based treatment for depression: a randomized controlled trial. Journal of affective disorders, 148(2-3), 331-337. https://doi.org/10.1016/j.jad.2012.12.020

22. Cavanagh, K., Strauss, C., Cicconi, F., Griffiths, N., Wyper, A., & Jones, F. (2013). A randomised controlled trial of a brief online mindfulness-based intervention. Behaviour research and therapy, 51(9), 573-578. https://doi.org/10.1016/j.brat.2013.06.003

23. Compen, F., Bisseling, E., Schellekens, M., Donders, R., Carlson, L., Van Der Lee, M., & Speckens, A. (2018). Face-to-face and internet-based mindfulness-based cognitive therapy compared with treatment as usual in reducing psychological distress in patients with cancer: a multicenter randomized controlled trial. Journal of Clinical Oncology, 36(23), 2413-2421. https://doi.org/10.1200/JCO.2017.76.566

24. Conley, C. S., Gonzales, C. H., Huguenel, B. M., Rauch, A. A., Kahrilas, I. J., Duffecy, J., & Silton, R. L. (2024). Benefits of a technology-delivered mindfulness intervention for psychological distress and positive wellbeing in depressed college students: Post-intervention and follow-up effects from an RCT. Mindfulness, 15(7), 1739-1758. https://doi.org/10.1007/s12671-024-02398-3

25. Davis, C. H., Donahue, M. L., Gaudiano, B. A., Uebelacker, L. A., Twohig, M. P., & Levin, M. E. (2024). Adding online storytelling-based acceptance and commitment therapy to antidepressant treatment for primary care patients: a randomized clinical trial. Cognitive behaviour therapy, 53(1), 48-69. https://doi.org/10.1080/16506073.2023.2265560

26. El Morr, C., Ritvo, P., Ahmad, F., Moineddin, R., & MVC Team. (2020). Effectiveness of an 8-week web-based mindfulness virtual community intervention for university students on symptoms of stress, anxiety, and depression: randomized controlled trial. JMIR mental health, 7(7), e18595. https://doi.org/10.2196/18595

27. El Rafihi-Ferreira, R., Hasan, R., Toscanini, A. C., Linares, I. M., Suzuki Borges, D., Brasil, I. P., ... & Morin, C. (2024). Acceptance and commitment therapy versus cognitive behavioral therapy for insomnia: A randomized controlled trial. Journal of consulting and clinical psychology, 92(6), 330. https://doi.org/10.1037/ccp0000881

28. El-Ashry, A. M., Elhay, E. S. A., Taha, S. M., Salem, E. S. A. E. H. E. S., & El-Sayed, M. M. (2023). Impact of virtual group-based acceptance and commitment therapy on social adjustment and work-family conflict among intern nurses: a randomized control trial. BMC psychiatry, 23(1), 552. https://doi.org/10.1186/s12888-023-05045-8

29. Eriksson, T., Germundsjö, L., Åström, E., & Rönnlund, M. (2018). Mindful self-compassion training reduces stress and burnout symptoms among practicing psychologists: A randomized controlled trial of a brief web-based intervention. Frontiers in psychology, 9, 419949. https://doi.org/10.3389/fpsyg.2018.02340

30. Eustis, E. H., Hayes-Skelton, S. A., Orsillo, S. M., & Roemer, L. (2018). Surviving and thriving during stress: a randomized clinical trial comparing a brief web-based therapist-assisted acceptance-based behavioral intervention versus waitlist control for college students. Behavior therapy, 49(6), 889-903. https://doi.org/10.1016/j.beth.2018.05.009

31. Fledderus, M., Bohlmeijer, E. T., Fox, J. P., Schreurs, K. M., & Spinhoven, P. (2013). The role of psychological flexibility in a self-help acceptance and commitment therapy intervention for psychological distress in a randomized controlled trial. Behaviour research and therapy, 51(3), 142-151. https://doi.org/10.1016/j.brat.2012.11.007

32. Flett, J. A., Hayne, H., Riordan, B. C., Thompson, L. M., & Conner, T. S. (2019). Mobile mindfulness meditation: a randomised controlled trial of the effect of two popular apps on mental health. Mindfulness, 10(5), 863-876. https://doi.org/10.1007/s12671-018-1050-9

33. Garcia, M. A., Rabinowitz, E. P., Levin, M. E., Shasteen, H., Allen, P. A., & Delahanty, D. L. (2023). Online acceptance and commitment therapy for chronic pain in a sample of people with Chiari malformation: A pilot study. Journal of Behavioral and Cognitive Therapy, 33(3), 152-168. https://doi.org/10.1016/j.jbct.2023.09.001

34. Ghorbani, S., Ashouri, A., Gharraee, B., & Farahani, H. (2022). Effectiveness of Online Group-mindfulness and Acceptance-based Therapy and Cognitive-behavioral Therapy on Misophonia. Iranian Journal of Psychiatry & Behavioral Sciences/Progress in Psychiatry & Behavioral Sciences, 16(2). https://doi.org/10.5812/ijpbs-120159

35. Girgenti, S. G., Dallasta, I., Lawrence, E., Merbach, D., Simon, J. Z., Llinas, R. H., ... & Marsh, E. B. (2025). Modified-mindfulness-based stress reduction as a treatment for cognitive recovery in patients with minor stroke: a randomized controlled pilot study. Frontiers in Neurology, 16, 1534480. https://doi.org/10.3389/fneur.2025.1534480

36. Glück, T. M., & Maercker, A. (2011). A randomized controlled pilot study of a brief web-based mindfulness training. BMC psychiatry, 11(1), 175. https://doi.org/10.1186/1471-244X-11-175

37. Godara, M., Hecht, M., & Singer, T. (2024). Training-related improvements in mental well-being through reduction in negative interpretation bias: A randomized trial of online socio-emotional dyadic and mindfulness interventions. Journal of Affective Disorders, 354, 662-672. https://doi.org/10.1016/j.jad.2024.03.037

38. González, C. R., Vega, B. R., Zarcero, M. T., Mediavilla, R., Molina, N. B., Fernández, R. P., ... & Tarrero, Á. P. (2022). Mindfulness based intervention through mobile app for colorectal cancer people awaiting surgery: A randomized clinical trial. Cirugía Española (English Edition), 100(12), 747-754. https://doi.org/10.1016/j.cireng.2022.08.008

39. Gorinelli, S., Gallego, A., Lappalainen, P., & Lappalainen, R. (2023). Virtual reality acceptance and commitment therapy intervention for social and public speaking anxiety: A randomized controlled trial. Journal of Contextual Behavioral Science, 28, 289-299. https://doi.org/10.1016/j.jcbs.2023.05.004

40. Grégoire, S., Beaulieu, F., Lachance, L., Bouffard, T., Vezeau, C., & Perreault, M. (2024). An online peer support program to improve mental health among university students: A randomized controlled trial. Journal of American College Health, 72(7), 2001-2013. https://doi.org/10.1080/07448481.2022.2099224

41. Gu, J., Cavanagh, K., & Strauss, C. (2018). Investigating the specific effects of an online mindfulness-based self-help intervention on stress and underlying mechanisms. Mindfulness, 9(4), 1245-1257. https://doi.org/10.1007/s12671-017-0867-y

42. Gu, C., Peng, Y., Lin, S., Yao, J., & Chen, X. (2024). Effects of a 4-week internet-delivered mindfulness-based cancer recovery program on anxiety, depression, and mindfulness among patients with breast cancer. Zhong nan da xue xue bao. Yi xue ban= Journal of Central South University. Medical Sciences, 49(7), 1130-1142. https://doi.org/10.11817/j.issn.1672-7347.2024.240028

43. Gutiérrez-Hernández, M. E., Fanjul Rodríguez, L. F., Díaz Megolla, A., Oyanadel, C., & Peñate Castro, W. (2023). The effect of daily meditative practices based on mindfulness and self-compassion on emotional distress under stressful conditions: A randomized controlled trial. European Journal of Investigation in Health, Psychology and Education, 13(4), 762-775. https://doi.org/10.3390/ejihpe13040058

44. Han, J., Zhao, Z., & Ren, Z. (2025). Effect and Mechanism of an ACT‐Based Psychological Resilience Intervention Targeting Students Failing in Postgraduate Entrance Examinations in China: A Randomized Controlled Trial. PsyCh Journal, 14(3), 344-356. https://doi.org/10.1002/pchj.825

45. Han, A., Oster, R., Yuen, H., Jenkins, J., Hawkins, J., & Edwards, L. (2025). Videoconference-delivered acceptance and commitment therapy for family caregivers of people with dementia: Pilot randomized controlled trial. JMIR Formative Research, 9, e67545. https://doi.org/10.2196/67545

46. Harper, S. (2024). Acceptance and commitment therapy for stress reduction in caregivers of children with autism spectrum disorder: a feasibility study (Doctoral dissertation, Fielding Graduate University). https://www.proquest.com/dissertations-theses/acceptance-commitment-therapy-stress-reduction/docview/3200610111/se-2

47. Hearn, J. H., & Finlay, K. A. (2018). Internet-delivered mindfulness for people with depression and chronic pain following spinal cord injury: a randomized, controlled feasibility trial. Spinal cord, 56(8), 750-761. https://doi.org/10.1038/s41393-018-0090-2

48. Hesser, H., Gustafsson, T., Lundén, C., Henrikson, O., Fattahi, K., Johnsson, E., ... & Andersson, G. (2012). A randomized controlled trial of Internet-delivered cognitive behavior therapy and acceptance and commitment therapy in the treatment of tinnitus. Journal of consulting and clinical psychology, 80(4), 649. https://doi.org/10.1037/a0027021

49. Hoge, E. A., Mete, M., Baker, A. W., Szuhany, K. L., Armstrong, C. H., Steinberg, M. H., ... & Simon, N. M. (2025). A randomized controlled trial comparing mindfulness to escitalopram for anxiety: In-person and remote, synchronous delivery pre and post COVID-19 pandemic. Journal of Affective Disorders, 384, 163-172. https://doi.org/10.1016/j.jad.2025.04.145

50. Huberty, J., Green, J., Glissmann, C., Larkey, L., Puzia, M., & Lee, C. (2019). Efficacy of the mindfulness meditation mobile app “calm” to reduce stress among college students: Randomized controlled trial. JMIR mHealth and uHealth, 7(6), e14273. https://doi.org/10.2196/14273

51. Hwang, H., Kim, S. M., Netterstrøm, B., & Han, D. H. (2022). The efficacy of a smartphone-based app on stress reduction: randomized controlled trial. Journal of medical Internet research, 24(2), e28703. https://doi.org/10.2196/28703

52. Hämäläinen, T., Lappalainen, P., Langrial, S. U., Lappalainen, R., & Kiuru, N. (2025). Mechanisms of change in an online acceptance and commitment therapy intervention for insomnia. Scientific Reports, 15(1), 2868. https://doi.org/10.1038/s41598-025-87018-3

53. Ivanova, E., Lindner, P., Ly, K. H., Dahlin, M., Vernmark, K., Andersson, G., & Carlbring, P. (2016). Guided and unguided Acceptance and Commitment Therapy for social anxiety disorder and/or panic disorder provided via the Internet and a smartphone application: A randomized controlled trial. Journal of anxiety disorders, 44, 27-35. https://doi.org/10.1016/j.janxdis.2016.09.012

54. Jelinek, L., Arlt, S., Moritz, S., Schröder, J., Westermann, S., & Cludius, B. (2020). Brief web-based intervention for depression: randomized controlled trial on behavioral activation. Journal of Medical Internet Research, 22(3), e15312. https://doi.org/10.2196/15312

55. Jing, S., Dai, Z., Liu, X., Yang, X., Cheng, J., Chen, T., ... & Wang, C. (2025). Effectiveness of neurofeedback-assisted and conventional 6-week web-based mindfulness interventions on mental health of Chinese nursing students: randomized controlled trial. Journal of medical Internet research, 27, e71741. https://doi.org/10.2196/71741

56. Juarez-Reyes, M., Martinez, E., Xiao, L., & Goldman Rosas, L. (2024). A randomized controlled trial of a culturally Adapted, Community-Based, remotely delivered mindfulness program for Latinx patients with breast cancer is acceptable and feasible while reducing anxiety. Global Advances in Integrative Medicine and Health, 13, 27536130241274240. https://doi.org/10.1177/27536130241274240

57. Kang, M. Y., Nan, J. K., & Yuan, Y. (2023). Effects and mechanisms of an online short‐term audio‐based mindfulness program on positive affect: A randomized controlled trial including exploratory moderator analyses. Applied Psychology: Health and Well‐Being, 15(3), 1150-1165. https://doi.org/10.1111/aphw.12431

58. Kang, M. Y., Nan, J. K., & Yuan, Y. (2023). Effectiveness of an online short-term Audio-based Mindfulness program on negative emotions during the COVID-19 pandemic: latent growth curve analyses of anxiety and moderated mediation effects of anxiety between Mindfulness and negative affect. Current Psychology, 42(34), 30049-30061. https://doi.org/10.1007/s12144-022-03902-5

59. Keng, S. L., Chin, J. W. E., Mammadova, M., & Teo, I. (2022). Effects of mobile app-based mindfulness practice on healthcare workers: a randomized active controlled trial. Mindfulness, 13(11), 2691-2704. https://doi.org/10.1007/s12671-022-01975-8

60. Kirk, U., & Axelsen, J. L. (2020). Heart rate variability is enhanced during mindfulness practice: A randomized controlled trial involving a 10-day online-based mindfulness intervention. PloS one, 15(12), e0243488. https://doi.org/10.1371/journal.pone.0243488

61. Kirk, U., Staiano, W., Hu, E., Ngnoumen, C., Kunkle, S., Shih, E., ... & Lee, L. (2023). App-based mindfulness for attenuation of subjective and physiological stress reactivity in a population with elevated stress: randomized controlled trial. JMIR mHealth and uHealth, 11, e47371. https://doi.org/10.2196/47371

62. Kor, P. P. K., Tsang, A. P. L., Lai, D. L. L., & Liu, J. Y. W. (2025). The effectiveness of a hybrid mindfulness-based intervention in reducing stress among informal caregivers of people with dementia: a randomized controlled trial. The Gerontologist, 65(12), gnaf281. https://doi.org/10.1093/geront/gnaf281

63. Krieger, T., Reber, F., von Glutz, B., Urech, A., Moser, C. T., Schulz, A., & Berger, T. (2019). An internet-based compassion-focused intervention for increased self-criticism: A randomized controlled trial. Behavior therapy, 50(2), 430-445. https://doi.org/10.1016/j.beth.2018.08.003

64. Lappalainen, P., Granlund, A., Siltanen, S., Ahonen, S., Vitikainen, M., Tolvanen, A., & Lappalainen, R. (2014). ACT Internet-based vs face-to-face? A randomized controlled trial of two ways to deliver Acceptance and Commitment Therapy for depressive symptoms: An 18-month follow-up. Behaviour research and therapy, 61, 43-54. https://doi.org/10.1016/j.brat.2014.07.006

65. Lappalainen, P., Langrial, S., Oinas-Kukkonen, H., Tolvanen, A., & Lappalainen, R. (2015). Web-based acceptance and commitment therapy for depressive symptoms with minimal support: a randomized controlled trial. Behavior modification, 39(6), 805-834. https://doi.org/10.1177/01454455155981

66. Lappalainen, P., Keinonen, K., Lappalainen, R., Selinheimo, S., Vuokko, A., Sainio, M., ... & Paunio, T. (2024). Online acceptance and commitment therapy (iACT) for adults with persistent physical symptoms–3-month follow-up study of a randomized controlled trial. Journal of Psychosomatic Research, 183, 111830. https://doi.org/10.1016/j.jpsychores.2024.111830

67. Larsson, A., Hartley, S., & McHugh, L. (2022). A randomised controlled trial of brief web-based acceptance and commitment Therapy on the general mental health, depression, anxiety and stress of college Students. Journal of Contextual Behavioral Science, 24, 10-17. https://doi.org/10.1016/j.jcbs.2022.02.005

68. Leadbitter, K., Langhorne, S., Smallman, R., Chu, P., Ellis, C., Harrison, L., ... & Green, J. (2025). Clinical effectiveness of an online psychoeducational and psychotherapeutic programme for caregivers of children newly diagnosed as autistic: a parallel, assessor-masked, randomised controlled trial in the UK (REACH-ASD). The Lancet Psychiatry, 12(4), 289-302. https://doi.org/10.1016/S2215-0366(25)00036-7

69. Levin, M. E., Pistorello, J., Seeley, J. R., & Hayes, S. C. (2014). Feasibility of a prototype web-based acceptance and commitment therapy prevention program for college students. Journal of American College Health, 62(1), 20-30. https://doi.org/10.1080/07448481.2013.843533

70. Levin, M. E., Haeger, J. A., Pierce, B. G., & Twohig, M. P. (2017). Web-based acceptance and commitment therapy for mental health problems in college students: A randomized controlled trial. Behavior modification, 41(1), 141-162. https://doi.org/10.1177/0145445516659645

71. Levin, M. E., Haeger, J., & Cruz, R. A. (2019). Tailoring acceptance and commitment therapy skill coaching in the moment through smartphones: Results from a randomized controlled trial. Mindfulness, 10(4), 689-699. https://doi.org/10.1007/s12671-018-1004-2

72. Levin, M. E., Krafft, J., Hicks, E. T., Pierce, B., & Twohig, M. P. (2020). A randomized dismantling trial of the open and engaged components of acceptance and commitment therapy in an online intervention for distressed college students. Behaviour Research and Therapy, 126, 103557. https://doi.org/10.1016/j.brat.2020.103557

73. Levin, M. E., Aller, T. B., Klimczak, K. S., Donahue, M. L., & Knudsen, F. M. (2025). Digital acceptance and commitment therapy for adults with chronic health conditions: Results from a waitlist-controlled trial. Behaviour Research and Therapy, 188, 104729. https://doi.org/10.1016/j.brat.2025.104729

74. Li, Y., Zhang, A. J., Meng, Y., Hofmann, S. G., Zhou, A. Y., & Liu, X. (2023). A randomized controlled trial of an online self-help mindfulness intervention for emotional distress: Serial mediating effects of mindfulness and experiential avoidance. Mindfulness, 14(3), 510-523. https://doi.org/10.1007/s12671-023-02083-x

75. Lin, J., Paganini, S., Sander, L., Lüking, M., Ebert, D. D., Buhrman, M., ... & Baumeister, H. (2017). An internet-based intervention for chronic pain: a three-arm randomized controlled study of the effectiveness of guided and unguided acceptance and commitment therapy. Deutsches Ärzteblatt International, 114(41), 681. https://doi.org/10.3238/arztebl.2017.0681

76. Listiyandini, R. A., Andriani, A., Afsari, N., Marfu’atun, E., Hafizah, N., Sholeh, A., ... & Newby, J. M. (2025). A culturally adapted internet-delivered mindfulness intervention with counsellor guidance for reducing distress among Indonesian university students: A randomised waitlist-controlled trial. Behaviour Research and Therapy, 104827. https://doi.org/10.1016/j.brat.2025.104827

77. Liu, L., Tian, L., Jiang, J., Zhang, Y., Chi, X., Liu, W., ... & Sun, P. (2023). Effect of an online mindfulness-based stress reduction intervention on postpandemic era nurses' subjective well-being, job burnout, and psychological adaptation. Holistic Nursing Practice, 37(5), 244-252. https://doi.org/10.1097/HNP.0000000000000603

78. Ljótsson, B., Falk, L., Vesterlund, A. W., Hedman, E., Lindfors, P., Rück, C., ... & Andersson, G. (2010). Internet-delivered exposure and mindfulness based therapy for irritable bowel syndrome–a randomized controlled trial. Behaviour research and therapy, 48(6), 531-539. https://doi.org/10.1016/j.brat.2010.03.003

79. Ljótsson, B., Hedman, E., Andersson, E., Hesser, H., Lindfors, P., Hursti, T., ... & Andersson, G. (2011). Internet-delivered exposure-based treatment vs. stress management for irritable bowel syndrome: a randomized trial. Official journal of the American College of Gastroenterology| ACG, 106(8), 1481-1491. https://doi.org/10.1038/ajg.2011.139

80. Lothes, J. E., Naseer, Z., & Perretti, B. (2025). The Effects of Mindfulness Practice on Anxiety and Test Anxiety in a College Student Population Utilizing a RCT and WLC. Psychological Reports, 00332941251358205. https://doi.org/10.1177/00332941251358205

81. Ly, K. H., Trüschel, A., Jarl, L., Magnusson, S., Windahl, T., Johansson, R., ... & Andersson, G. (2014). Behavioural activation versus mindfulness-based guided self-help treatment administered through a smartphone application: a randomised controlled trial. BMJ open, 4(1), e003440. https://doi.org/10.1136/bmjopen-2013-003440

82. Lynn, S., & Basso, J. C. (2023). Effects of a neuroscience-based mindfulness meditation program on psychological health: Pilot randomized controlled trial. JMIR Formative Research, 7(1), e40135. https://doi.org/10.2196/40135

83. Maathz, P., McCracken, L. M., Eriksson, V., Säde, F., Aneblom, G., Rikner, Å., ... & Buhrman, M. (2023). A feasibility trial of online acceptance and commitment therapy for women with provoked vestibulodynia. Scandinavian Journal of Pain, 23(3), 476-482. https://doi.org/10.1515/sjpain-2022-0146

84. Maddock, A., McGuigan, K., & McCusker, P. (2023). A randomised trial of mindfulness-based social work and self-care with social workers. Current Psychology, 42(11), 9170-9183. https://doi.org/10.1007/s12144-023-04410-w

85. Mak, W. W., Tong, A. C., Yip, S. Y., Lui, W. W., Chio, F. H., Chan, A. T., & Wong, C. C. (2018). Efficacy and moderation of mobile app–based programs for mindfulness-based training, self-compassion training, and cognitive behavioral psychoeducation on mental health: Randomized controlled noninferiority trial. JMIR mental health, 5(4), e60. https://doi.org/10.2196/mental.8597

86. Martel, M. E., Dionne, F., Pagé, M. G., & Choiniere, M. (2024). A randomized controlled trial comparing two guided self-help Acceptance and Commitment Therapy formats to education on pain. Journal of Contextual Behavioral Science, 32, 100760. https://doi.org/10.1016/j.jcbs.2024.100760

87. Meyer, B., Berger, T., Caspar, F., Beevers, C., Andersson, G., & Weiss, M. (2009). Effectiveness of a novel integrative online treatment for depression (Deprexis): randomized controlled trial. Journal of medical Internet research, 11(2), e1151. https://doi.org/10.2196/jmir.1151

88. Modrego-Alarcón, M., López-del-Hoyo, Y., García-Campayo, J., Pérez-Aranda, A., Navarro-Gil, M., Beltrán-Ruiz, M., ... & Montero-Marin, J. (2021). Efficacy of a mindfulness-based programme with and without virtual reality support to reduce stress in university students: A randomized controlled trial. Behaviour research and therapy, 142, 103866. https://doi.org/10.1016/j.brat.2021.103866

89. Molander, P., Hesser, H., Weineland, S., Bergwall, K., Buck, S., Jäder Malmlöf, J., ... & Andersson, G. (2018). Internet-based acceptance and commitment therapy for psychological distress experienced by people with hearing problems: a pilot randomized controlled trial. Cognitive behaviour therapy, 47(2), 169-184. https://doi.org/10.1080/16506073.2017.1365929

90. Moore, S., Mavaddat, N., Auret, K., Hassed, C., Chambers, R., Sinclair, C., ... & Ngo, H. (2024). The Western Australian medical schools mindfulness project: a randomised controlled trial. BMC Medical Education, 24(1), 1182. https://doi.org/10.1186/s12909-024-06128-0

91. Naude, C., Skvarc, D., Maunick, B., Evans, S., Romano, D., Chesterman, S., ... & Mikocka-Walus, A. (2025). Acceptance and commitment therapy for adults living with inflammatory bowel disease and distress: A randomized controlled trial. Official journal of the American College of Gastroenterology| ACG, 120(8), 1839-1851. https://doi.org/10.14309/ajg.0000000000003032

92. Nicolescu, S., Secară, E. C., Jiboc, N. M., & Băban, A. (2024). Oncovox: A randomised controlled trial of a web-based acceptance and commitment therapy for breast cancer patients. Journal of Contextual Behavioral Science, 32, 100729. https://doi.org/10.1016/j.jcbs.2024.100729

93. Nissen, E. R., O'Connor, M., Kaldo, V., Højris, I., Borre, M., Zachariae, R., & Mehlsen, M. (2020). Internet‐delivered mindfulness‐based cognitive therapy for anxiety and depression in cancer survivors: A randomized controlled trial. Psycho‐oncology, 29(1), 68-75. https://doi.org/10.1002/pon.5237

94. Norman-Nott, N., Briggs, N. E., Hesam-Shariati, N., Wilks, C. R., Schroeder, J., Diwan, A. D., ... & Gustin, S. M. (2025). Online dialectical behavioral therapy for emotion dysregulation in people with chronic pain: A randomized clinical trial. JAMA Network Open, 8(5), e256908. https://doi.org/10.1001/jamanetworkopen.2025.6908

95. O'Hayer, C. V., Smith, P. J., Drescher, C. F., Bruschwein, H., Nurse, C. N., Kushner, H. M., ... & Hoag, J. B. (2024). ACT with CF: A randomized trial of acceptance and commitment therapy vs supportive psychotherapy for adults with cystic fibrosis. General Hospital Psychiatry, 91, 212-222. https://doi.org/10.1016/j.genhosppsych.2024.11.011

96. Orosa-Duarte, Á., Mediavilla, R., Muñoz-Sanjose, A., Palao, Á., Garde, J., López-Herrero, V., ... & Rodríguez-Vega, B. (2021). Mindfulness-based mobile app reduces anxiety and increases self-compassion in healthcare students: a randomised controlled trial. Medical teacher, 43(6), 686-693. https://doi.org/10.1080/0142159X.2021.1887835

97. Otared, N., Moharrampour, N. G., Vojoudi, B., & Najafabadi, A. J. (2021). A group-based online acceptance and commitment therapy treatment for depression, anxiety symptoms and quality of life in healthcare workers during COVID-19 pandemic: A randomized controlled trial. International journal of psychology and psychological therapy, 21(3), 399-411. https://www.proquest.com/scholarly-journals/group-based-online-acceptance-commitment-therapy/docview/2587957666/se-2

98. Pagnini, F., Phillips, D., Haulman, A., Bankert, M., Simmons, Z., & Langer, E. (2022). An online non-meditative mindfulness intervention for people with ALS and their caregivers: a randomized controlled trial. Amyotrophic Lateral Sclerosis and Frontotemporal Degeneration, 23(1-2), 116-127. https://doi.org/10.1080/21678421.2021.1928707

99. Park, C., Song, H., Kim, H., Kim, E., Jo, H. J., Kim, J. J., ... & Kim, J. (2025). Efficacy of a Smartphone-Based Digital Therapeutic (Anzeilax) in Generalized Anxiety Disorder: Randomized Controlled Trial. Journal of medical Internet research, 27, e69981. https://doi.org/10.2196/69981

100. Peros, O. M. (2022). Self-Guided, Internet-Based Dialectical Behavior Therapy Skills for Transdiagnostic Emotion Dysregulation: A Randomized Controlled Pilot Study. Hofstra University. https://www.proquest.com/dissertations-theses/self-guided-internet-based-dialectical-behavior/docview/2645880954/se-2

101. Pinto, C., Norton, S., Cubi-Molla, P., Hurt, C., Chowdhury, S., Dooley, N., ... & Bogosian, A. (2025). Evaluating a digital mental health intervention for people with Parkinson’s (PACT): acceptability and feasibility randomised controlled trial. Aging & Mental Health, 29(9), 1694-1702. https://doi.org/10.1080/13607863.2025.2478508

102. Poole, N., Cope, S., Vanzan, S., Duffus, A., Williams, T., Mantovani, N., ... & Howard, R. (2025). Randomised controlled feasibility trial of online group acceptance and commitment therapy for functional cognitive disorder. BJPsych open, 11(3), e91. https://doi.org/10.1192/bjo.2025.33

103. Pots, W. T., Fledderus, M., Meulenbeek, P. A., ten Klooster, P. M., Schreurs, K. M., & Bohlmeijer, E. T. (2016). Acceptance and commitment therapy as a web-based interventionfor depressive symptoms: Randomised controlled trial. The British Journal of Psychiatry, 208(1), 69-77. https://doi.org/10.1192/bjp.bp.114.146068

104. Querstret, D., Cropley, M., & Fife-Schaw, C. (2018). The effects of an online mindfulness intervention on perceived stress, depression and anxiety in a non-clinical sample: a randomised waitlist control trial. Mindfulness, 9(6), 1825-1836. https://doi.org/10.1007/s12671-018-0925-0

105. Rabasco, A., Wallace, G. T., & Andover, M. (2025). Mindfulness for reducing everyday suicidal thoughts (Mind-REST): A daily mindfulness intervention for adults with suicidal ideation. Cognitive therapy and research, 49(1), 155-168. https://doi.org/10.1007/s10608-024-10516-7

106. Rabinowitz, E. P. (2025). Is Coaching Necessary for Acceptance and Commitment Therapy (ACT) Treatment Efficacy and Engagement? A Three-Group Randomized Controlled Trial of Online Act for Chronic Pain in Patients With Chiari Malformation. Kent State University. https://www.proquest.com/dissertations-theses/is-coaching-necessary-acceptance-commitment/docview/3250989847/se-2

107. Repo, S., Elovainio, M., Pyörälä, E., Iriarte-Lüttjohann, M., Tuominen, T., Härkönen, T., ... & Paunio, T. (2022). Comparison of two different mindfulness interventions among health care students in Finland: a randomised controlled trial: S. Repo et al. Advances in Health Sciences Education, 27(3), 709-734. https://doi.org/10.1007/s10459-022-10116-8

108. Rickardsson, J., Gentili, C., Holmström, L., Zetterqvist, V., Andersson, E., Persson, J., ... & Wicksell, R. K. (2021). Internet‐delivered acceptance and commitment therapy as microlearning for chronic pain: A randomized controlled trial with 1‐year follow‐up. European Journal of Pain, 25(5), 1012-1030. https://doi.org/10.1002/ejp.1723

109. Ritvo, P., Knyahnytska, Y., Pirbaglou, M., Wang, W., Tomlinson, G., Zhao, H., ... & Daskalakis, Z. (2021). Online mindfulness-based cognitive behavioral therapy intervention for youth with major depressive disorders: randomized controlled trial. Journal of medical Internet research, 23(3), e24380. https://doi.org/10.2196/24380

110. Roepke, A. M., Jaffee, S. R., Riffle, O. M., McGonigal, J., Broome, R., & Maxwell, B. (2015). Randomized controlled trial of SuperBetter, a smartphone-based/internet-based self-help tool to reduce depressive symptoms. Games for health journal, 4(3), 235-246. https://doi.org/10.1089/g4h.2014.0046

111. Räsänen, P., Lappalainen, P., Muotka, J., Tolvanen, A., & Lappalainen, R. (2016). An online guided ACT intervention for enhancing the psychological wellbeing of university students: A randomized controlled clinical trial. Behaviour research and therapy, 78, 30-42. https://doi.org/10.1016/j.brat.2016.01.001

112. Sairanen, E., Lappalainen, R., Lappalainen, P., Kaipainen, K., Carlstedt, F., Anclair, M., & Hiltunen, A. (2019). Effectiveness of a web-based Acceptance and Commitment Therapy intervention for wellbeing of parents whose children have chronic conditions: A randomized controlled trial. Journal of Contextual Behavioral Science, 13, 94-102. https://doi.org/10.1016/j.jcbs.2019.07.004

113. Schechter, M. S., Molzhon, A., Everhart, R. S., Kang, L., Weiskittle, R., Castleberry, B., & Thompson, N. J. (2025). Impact of UPLIFT, a group telehealth intervention, on symptoms of depression and anxiety in adults with CF. Journal of Cystic Fibrosis, 24(2), 319-325. https://doi.org/10.1016/j.jcf.2024.11.008

114. Scott, W., Guildford, B. J., Badenoch, J., Driscoll, E., Chilcot, J., Norton, S., ... & McCracken, L. M. (2021). Feasibility randomized‐controlled trial of online acceptance and commitment therapy for painful peripheral neuropathy in people living with HIV: the OPEN study. European Journal of Pain, 25(7), 1493-1507. https://doi.org/10.1002/ejp.1762

115. Segal, Z. V., Dimidjian, S., Beck, A., Boggs, J. M., Vanderkruik, R., Metcalf, C. A., ... & Levy, J. (2020). Outcomes of online mindfulness-based cognitive therapy for patients with residual depressive symptoms: a randomized clinical trial. JAMA psychiatry, 77(6), 563-573. https://doi.org/10.1001/jamapsychiatry.2019.4693

116. Segal, O., Hevron, H., Sher, H., Aderka, I. M., & Weinbach, N. (2025). A randomized controlled trial of an online dialectical behavior therapy skills intervention for war-related distress. Journal of Consulting and Clinical Psychology, 93(7), 500. https://doi.org/10.1037/ccp0000958

117. Sengupta, A., & Wagani, R. (2024). Mindful self-compassion for psychological distress associated with skin conditions: An online intervention study. Indian Journal of Dermatology, Venereology and Leprology, 91(1), 71-75. https://doi.org/10.25259/IJDVL_451_2023

118. Shahidi, S., Zargar, F., Khajelangi, H. A., & Tarrahi, M. J. (2023). The effect of internet-delivered mindfulness stress reduction combined with acceptance and commitment therapy on health anxiety and quality of life of caregiver of patients infected by COVID-19: A randomized clinical trial. International journal of community based nursing and midwifery, 11(2), 110. https://doi.org/10.30476/IJCBNM.2023.97271.2172

119. Simister, H. D., Tkachuk, G. A., Shay, B. L., Vincent, N., Pear, J. J., & Skrabek, R. Q. (2018). Randomized controlled trial of online acceptance and commitment therapy for fibromyalgia. The Journal of Pain, 19(7), 741-753. https://doi.org/10.1016/j.jpain.2018.02.004

120. Stjernswärd, S., & Hansson, L. (2017). Effectiveness and usability of a web-based mindfulness intervention for families living with mental illness. Mindfulness, 8(3), 751-764. https://doi.org/10.1007/s12671-016-0653-2

121. Sylvia, L. G., Lunn, M. R., Obedin-Maliver, J., McBurney, R. N., Nowell, W. B., Nosheny, R. L., ... & Nierenberg, A. A. (2022). Web-based mindfulness-based interventions for Well-being: Randomized Comparative Effectiveness Trial. Journal of medical Internet research, 24(9), e35620. https://doi.org/10.2196/35620

122. Taylor, H., Cavanagh, K., Field, A. P., & Strauss, C. (2022). Health care workers’ need for headspace: findings from a multisite definitive randomized controlled trial of an unguided digital mindfulness-based self-help app to reduce healthcare worker stress. JMIR mHealth and uHealth, 10(8), e31744. https://doi.org/10.2196/31744

123. Thompson, E. M., Albertella, L., Viskovich, S., Pakenham, K. I., & Fontenelle, L. F. (2024). Internet-based acceptance and commitment therapy for obsessive-compulsive symptoms: a randomized controlled trial. Behaviour Research and Therapy, 180, 104595. https://doi.org/10.1016/j.brat.2024.104595

124. Tonkuş, M. B., & Bilgin, H. (2026). Effects of mindfulness-based stress reduction on ICU nurses’ resilience and well-being. Intensive and Critical Care Nursing, 93, 104258. https://doi.org/10.1016/j.iccn.2025.104258

125. Torok, M., Han, J., McGillivray, L., Wong, Q., Werner-Seidler, A., O’Dea, B., ... & Christensen, H. (2022). The effect of a therapeutic smartphone application on suicidal ideation in young adults: findings from a randomized controlled trial in Australia. PLoS Medicine, 19(5), e1003978. https://doi.org/10.1371/journal.pmed.1003978

126. Trompetter, H. R., Bohlmeijer, E. T., Fox, J. P., & Schreurs, K. M. (2015). Psychological flexibility and catastrophizing as associated change mechanisms during online Acceptance & Commitment Therapy for chronic pain. Behaviour research and therapy, 74, 50-59. https://doi.org/10.1016/j.brat.2015.09.001

127. Tu, Y. H., Liu, C., Gao, Z. H., & Chen, H. (2025). The effects of short video app–guided mindfulness meditation on rumination, self-compassion, psychological flexibility, and depression among individuals experiencing entrepreneurial failure. Frontiers in Psychiatry, 16, 1706661. https://doi.org/10.3389/fpsyt.2025.1706661

128. Türkoğlu, N., & Kavuran, E. (2024). The effect of mindfulness-based stress reduction program on stress and quality of life in family caregivers of patients with cancer: randomized controlled trial. Mindfulness, 15(5), 1070-1079. https://doi.org/10.1007/s12671-024-02336-3

129. Vally, Z., Shah, H., Varga, S. I., Hassan, W., Kashakesh, M., Albreiki, W., & Helmy, M. (2024). An internet-delivered acceptance and commitment therapy program for anxious affect, depression, and wellbeing: A randomized, parallel, two-group, waitlist-controlled trial in a Middle Eastern sample of college students. Plos one, 19(12), e0313243. https://doi.org/10.1371/journal.pone.0313243

130. Viravan, N., Atsariyasing, W., Srifuengfung, M., & Lortrakul, J. (2022). Efficacy of Online Mindfulness Program'Mindful Senses' for Depression and Anxiety Reduction in Community Samples: A Randomized Controlled Trial. Journal of the Medical Association of Thailand, 105(11). https://doi.org/10.35755/jmedassocthai.2022.11.13700

131. Viskovich, S., & Pakenham, K. I. (2020). Randomized controlled trial of a web‐based Acceptance and Commitment Therapy (ACT) program to promote mental health in university students. Journal of clinical psychology, 76(6), 929-951. https://doi.org/10.1002/jclp.22848

132. Wallace-Boyd, K., Boggiss, A. L., Ellett, S., Booth, R., Slykerman, R., & Serlachius, A. S. (2023). ACT2COPE: A pilot randomised trial of a brief online acceptance and commitment therapy intervention for people living with chronic health conditions during the COVID-19 pandemic. Cogent Psychology, 10(1), 2208916. https://doi.org/10.1080/23311908.2023.2208916

133. Wang, D., Lin, B., Zhang, S., Xu, W., & Liu, X. (2024). Effectiveness of an Internet-Based Self-Help acceptance and commitment therapy program on medical students’ mental Well-Being: Follow-Up randomized controlled trial. Journal of medical Internet research, 26, e50664. https://doi.org/10.2196/50664

134. Wang, D., Lin, B., Du, J., Liu, W., Sun, T., Li, Q., & Xiao, L. (2025). Acceptance and commitment therapy for nurses’ sleep, rumination, psychological flexibility, and it's neural mechanism: A randomized controlled fNIRS study. International Journal of Clinical and Health Psychology, 25(1), 100543. https://doi.org/10.1016/j.ijchp.2025.100543

135. Watt, M., Hyde, A., Spence, J., Wright, G., Vander Well, S., Johnson, E., ... & Tandon, P. (2022). A randomized control trial evaluating the impact of a web-based mind-body wellness intervention for patients with primary biliary cholangitis. Journal of Hepatology, 77, S338. https://doi.org/10.1016/S0168-8278(22)01038-8

136. Whittingham, K., Sheffield, J., Mak, C., Wright, A., & Boyd, R. N. (2022). Parenting acceptance and commitment therapy: an RCT of an online course with families of children with CP. Behaviour Research and Therapy, 155, 104129. https://doi.org/10.1016/j.brat.2022.104129

137. Xiong, W., Yu, X., Yu, L., & Yang, F. (2025). Effect of digital mindfulness on perceived stress and anxious emotion among college students. Frontiers in Psychiatry, 16, 1644370. https://doi.org/10.3389/fpsyt.2025.1644370

138. Xu, H., Eley, R., Kynoch, K., & Tuckett, A. (2022). Effects of mobile mindfulness on emergency department work stress: a randomised controlled trial. Emergency Medicine Australasia, 34(2), 176-185. https://doi.org/10.1111/1742-6723.13836

139. Yay-Pence, A., Aslan, S., Coldur, M., Atalay, Z., Beba, B., Coskun-Sayin, C., ... & Ekmekci-Ertek, I. (2025). Online mindfulness-based vs. cognitive-behavioural stress reduction for medical students: A randomized controlled trial. Psychiatry Research, 116632. https://doi.org/10.1016/j.psychres.2025.116632

140. Ye, Q., Huang, Y., Ge, X., & Song, X. (2023). Validation of online mindfulness-enhanced course for stress reduction in teachers. Frontiers in psychiatry, 14, 1086142. https://doi.org/10.3389/fpsyt.2023.1086142

141. Young, D. K. W., Carlbring, P., Ng, P. Y. N., Cheng, D. Y. T., Chen, J. Q. R., & Ng, S. M. (2023). Low-intensity online mindfulness-based intervention for university students with anxiety during the COVID-19 pandemic—A randomized controlled trial with 3-month follow-up. Internet Interventions, 34, 100665. https://doi.org/10.1016/j.invent.2023.100665

142. Yslado-Méndez, R., Escobar-Agreda, S., Villarreal-Zegarra, D., Trejo Flores, W. M., Sánchez-Broncano, J. D., Vilela-Estrada, A. L., ... & Rojas-Mezarina, L. (2025). Effectiveness, usability, and satisfaction of a self-administered digital intervention for reducing depression, anxiety, and stress in a university community in the Andean Region of Peru: randomized controlled trial. JMIR Formative Research, 9, e71465. https://doi.org/10.2196/71465

143. Zhang, Y., Liu, C., Chen, X., Zhang, Y., Li, Y., & Hu, X. (2024). Effects of Web‐Based Acceptance and Commitment Therapy on Health‐Related Outcomes Among Patients With Lung Cancer: A Feasibility Randomized Controlled Trial. Psycho‐Oncology, 33(12), e70045. https://doi.org/10.1002/pon.70045

144. Zhao, C., Wampold, B. E., Ren, Z., Zhang, L., & Jiang, G. (2022). The efficacy and optimal matching of an Internet‐based acceptance and commitment therapy intervention for depressive symptoms among university students: A randomized controlled trial in China. Journal of Clinical Psychology, 78(7), 1354-1375. https://doi.org/10.1002/jclp.23329

145. Zhao, C., Ren, Z., Jiang, G., & Zhang, L. (2022). Mechanisms of change in an Internet-Based ACT study for depression in China. Journal of Contextual Behavioral Science, 24, 51-59. https://doi.org/10.1016/j.jcbs.2022.03.004

146. Zhao, C., Zhao, Z., Levin, M. E., Lai, L., Shi, C., Hu, J., ... & Ren, Z. (2023). Efficacy and acceptability of mobile application-delivered acceptance and commitment therapy for posttraumatic stress disorder in China: A randomized controlled trial. Behaviour Research and Therapy, 171, 104440. https://doi.org/10.1016/j.brat.2023.104440

147. Zhu, T., Zhang, L., Weng, W., Gan, R., Sun, L., Wei, Y., ... & Chen, S. (2025). Effectiveness of an Internet-Based, Self-Guided, Short-Term Mindfulness Training (ISSMT) Program for Relieving Depressive Symptoms in the Adult Population in China: Single-Blind, Randomized Controlled Trial. Journal of Medical Internet Research, 27, e55583. https://doi.org/10.2196/55583
