# Supplementary material for: Impacts of digital third-wave interventions for adults on wellbeing and mental health outcomes: A systematic review and meta-analysis of randomized controlled trials
Source: PLOS Digit Health. 2026 Oct 1;5(10):e0001764. doi: 10.1371/journal.pdig.0001764 (PMC13630215; doi:10.1371/journal.pdig.0001764)
Supplement: Supplementary file 7 [file pdig.0001764.s007.docx]

**S7 Appendix. Full subgroup analyses.**

Text A.

To provide full transparency of moderator findings, detailed subgroup analyses are presented in Tables A–D. These tables report all subgroup estimates across outcomes and follow-up periods, including pooled effect sizes (Hedges’ g), 95% confidence intervals, prediction intervals, heterogeneity statistics (I², τ², Q), and study counts.

**Comparator Subgroups**

Full comparator-stratified results are presented in Table A. Across outcomes, effects were consistently smaller when digital third-wave interventions were compared against active control conditions than against inactive controls. This attenuation was most pronounced at post-intervention for depression, anxiety, and stress, where active-control comparisons yielded modest pooled effects relative to the larger effects observed versus waitlist or usual care. At follow-up, comparator differences persisted in several domains, although fewer trials contributed to longer-term estimates.

Table A.

| Outcome | Timepoint | Subgroup Level | k | Effect (g) | 95% CI | p | I² (%) | Prediction interval | τ² | Q | df(Q) | p(Q) |
| --- | --- | --- | --- | --- | --- | --- | --- | --- | --- | --- | --- | --- |
| Wellbeing | Post-intervention | Active | 12 | 0.15 | 0.06 to 0.24 | .002 | 32 | -0.04 to 0.33 | 0.007 | 16.17 | 11 | .135 |
| Wellbeing | Post-intervention | Inactive | 38 | 0.44 | 0.27 to 0.60 | <.001 | 83 | -0.47 to 1.34 | 0.206 | 219.93 | 37 | <.001 |
| Wellbeing | Short-term | Active | 6 | 0.02 | -0.06 to 0.09 | .615 | 0 | -0.06 to 0.09 | 0 | 2.94 | 5 | .709 |
| Wellbeing | Short-term | Inactive | 12 | 0.33 | 0.12 to 0.53 | .002 | 64 | -0.26 to 0.91 | 0.078 | 30.47 | 11 | .001 |
| Wellbeing | Medium-term | Active | 4 | 0.14 | -0.08 to 0.36 | .219 | 49 | -0.24 to 0.52 | 0.024 | 5.89 | 3 | .117 |
| Wellbeing | Medium-term | Inactive | 5 | 0.11 | -0.11 to 0.34 | .328 | 50 | -0.31 to 0.53 | 0.032 | 7.96 | 4 | .093 |
| Wellbeing | Long-term | Active | 2 | 0.26 | -0.06 to 0.59 | .114 | 0 | -0.06 to 0.59 | 0 | 0.2 | 1 | .658 |
| Wellbeing | Long-term | Inactive | 1 | 0.17 | -0.09 to 0.42 | .207 | 0 |  | 0 | 0 | 0 |  |
| Depression | Post-intervention | Active | 30 | 0.2 | 0.11 to 0.30 | <.001 | 57 | -0.16 to 0.56 | 0.032 | 66.72 | 29 | <.001 |
| Depression | Post-intervention | Inactive | 89 | 0.51 | 0.42 to 0.60 | <.001 | 76 | -0.18 to 1.20 | 0.122 | 365.07 | 88 | <.001 |
| Depression | Short-term | Active | 13 | 0.2 | 0.07 to 0.32 | .002 | 47 | -0.10 to 0.49 | 0.019 | 22.5 | 12 | .032 |
| Depression | Short-term | Inactive | 30 | 0.44 | 0.32 to 0.57 | <.001 | 61 | -0.07 to 0.96 | 0.065 | 73.62 | 29 | <.001 |
| Depression | Medium-term | Active | 8 | 0.28 | -0.04 to 0.61 | .087 | 87 | -0.61 to 1.18 | 0.183 | 55.89 | 7 | <.001 |
| Depression | Medium-term | Inactive | 14 | 0.31 | 0.17 to 0.45 | <.001 | 38 | -0.02 to 0.64 | 0.023 | 20.84 | 13 | .076 |
| Depression | Long-term | Active | 3 | -0.07 | -0.62 to 0.48 | .807 | 72 | -1.05 to 0.91 | 0.17 | 7.12 | 2 | .028 |
| Depression | Long-term | Inactive | 2 | 0.34 | 0.02 to 0.66 | .040 | 35 | -0.11 to 0.78 | 0.024 | 1.54 | 1 | .215 |
| Anxiety | Post-intervention | Active | 29 | 0.16 | 0.11 to 0.21 | <.001 | 0 | 0.11 to 0.21 | 0 | 25.16 | 28 | .619 |
| Anxiety | Post-intervention | Inactive | 74 | 0.47 | 0.37 to 0.57 | <.001 | 79 | -0.27 to 1.21 | 0.14 | 355.07 | 73 | <.001 |
| Anxiety | Short-term | Active | 12 | 0.21 | 0.05 to 0.37 | .009 | 65 | -0.21 to 0.63 | 0.039 | 31.85 | 11 | <.001 |
| Anxiety | Short-term | Inactive | 26 | 0.46 | 0.30 to 0.62 | <.001 | 76 | -0.26 to 1.18 | 0.129 | 104.13 | 25 | <.001 |
| Anxiety | Medium-term | Active | 7 | 0.15 | -0.03 to 0.34 | .098 | 60 | -0.25 to 0.56 | 0.035 | 15.03 | 6 | .020 |
| Anxiety | Medium-term | Inactive | 11 | 0.28 | 0.17 to 0.38 | <.001 | 0 | 0.17 to 0.38 | 0 | 7.33 | 10 | .694 |
| Anxiety | Long-term | Active | 2 | -0.14 | -0.50 to 0.23 | .457 | 30 | -0.60 to 0.33 | 0.022 | 1.42 | 1 | .233 |
| Anxiety | Long-term | Inactive | 1 | 0.38 | 0.16 to 0.59 | <.001 | 0 |  | 0 | 0 | 0 |  |
| Stress | Post-intervention | Active | 23 | 0.3 | 0.18 to 0.42 | <.001 | 69 | -0.14 to 0.74 | 0.046 | 71.37 | 22 | <.001 |
| Stress | Post-intervention | Inactive | 50 | 0.52 | 0.40 to 0.63 | <.001 | 77 | -0.20 to 1.23 | 0.129 | 213.47 | 49 | <.001 |
| Stress | Short-term | Active | 9 | 0.07 | -0.01 to 0.15 | .094 | 8 | -0.04 to 0.18 | 0.001 | 8.67 | 8 | .371 |
| Stress | Short-term | Inactive | 18 | 0.51 | 0.33 to 0.69 | <.001 | 69 | -0.12 to 1.14 | 0.095 | 54.36 | 17 | <.001 |
| Stress | Medium-term | Active | 5 | 0.31 | 0.08 to 0.55 | .009 | 63 | -0.15 to 0.78 | 0.042 | 10.74 | 4 | .030 |
| Stress | Medium-term | Inactive | 5 | 0.31 | 0.14 to 0.48 | <.001 | 0 | 0.14 to 0.48 | 0 | 2.91 | 4 | .574 |
| Stress | Long-term | Active | 2 | -0.17 | -0.94 to 0.60 | .666 | 71 | -1.37 to 1.03 | 0.221 | 3.4 | 1 | .065 |
| Stress | Long-term | Inactive | 1 | 0.31 | 0.07 to 0.56 | .013 | 0 |  | 0 | 0 | 0 |  |
| Psychological flexibility | Post-intervention | Active | 11 | 0.05 | -0.16 to 0.27 | .614 | 62 | -0.53 to 0.64 | 0.076 | 26.65 | 10 | .003 |
| Psychological flexibility | Post-intervention | Inactive | 43 | 0.47 | 0.32 to 0.61 | <.001 | 82 | -0.39 to 1.32 | 0.185 | 230.17 | 42 | <.001 |
| Psychological flexibility | Short-term | Active | 5 | 0.35 | 0.14 to 0.56 | .001 | 0 | 0.14 to 0.56 | 0 | 3.54 | 4 | .472 |
| Psychological flexibility | Short-term | Inactive | 16 | 0.58 | 0.22 to 0.94 | .001 | 90 | -0.81 to 1.97 | 0.471 | 157.74 | 15 | <.001 |
| Psychological flexibility | Medium-term | Active | 5 | -0.01 | -0.38 to 0.37 | .972 | 78 | -0.83 to 0.81 | 0.138 | 17.95 | 4 | .001 |
| Psychological flexibility | Medium-term | Inactive | 7 | 0.09 | -0.18 to 0.36 | .523 | 69 | -0.55 to 0.73 | 0.088 | 19.67 | 6 | .003 |
| Psychological flexibility | Long-term | Active | 3 | 0.11 | -0.45 to 0.67 | .693 | 68 | -0.86 to 1.09 | 0.166 | 6.25 | 2 | .044 |
| Psychological flexibility | Long-term | Inactive | 1 | 0.1 | -0.17 to 0.36 | .470 | 0 |  | 0 | 0 | 0 |  |
| Quality of life | Post-intervention | Active | 6 | 0.15 | -0.06 to 0.36 | .170 | 9 | -0.11 to 0.41 | 0.006 | 5.46 | 5 | .362 |
| Quality of life | Post-intervention | Inactive | 27 | 0.32 | 0.19 to 0.45 | <.001 | 62 | -0.21 to 0.84 | 0.068 | 68.97 | 26 | <.001 |
| Quality of life | Short-term | Active | 3 | 0.13 | -0.17 to 0.44 | .395 | 0 | -0.17 to 0.44 | 0 | 0.45 | 2 | .800 |
| Quality of life | Short-term | Inactive | 8 | 0.58 | 0.19 to 0.96 | .003 | 77 | -0.45 to 1.60 | 0.234 | 30.84 | 7 | <.001 |
| Quality of life | Medium-term | Active | 1 | -0.01 | -0.48 to 0.46 | .971 | 0 |  | 0 | 0 | 0 |  |
| Quality of life | Medium-term | Inactive | 6 | 0.09 | -0.13 to 0.30 | .424 | 38 | -0.30 to 0.47 | 0.026 | 8.12 | 5 | .150 |
| Quality of life | Long-term | Active | 1 | -0.42 | -0.93 to 0.09 | .105 | 0 |  | 0 | 0 | 0 |  |
| Quality of life | Long-term | Inactive | 2 | 0.27 | -0.03 to 0.57 | .082 | 69 | -0.20 to 0.74 | 0.034 | 3.27 | 1 | .071 |

**Population Subgroups**

Population-stratified analyses are presented in Table B. Effects were generally observed across Healthy, mental health condition, and medical condition samples at post-intervention, although magnitudes varied by outcome. In several cases, medical condition samples demonstrated effect sizes comparable to, or larger than, those observed in mental health samples. At follow-up timepoints, subgroup estimates were based on fewer contributing trials, and between-population differences were less consistent. No single population category uniformly drove effects across all outcomes.

Table B.

| Outcome | Timepoint | Subgroup Level | k | Effect (g) | 95% CI | p | I² (%) | Prediction interval | τ² | Q | df(Q) | p(Q) |
| --- | --- | --- | --- | --- | --- | --- | --- | --- | --- | --- | --- | --- |
| Wellbeing | Post-intervention | Healthy | 19 | 0.32 | 0.19 to 0.46 | <.001 | 73 | -0.14 to 0.79 | 0.052 | 66.29 | 18 | <.001 |
| Wellbeing | Post-intervention | Mental health condition | 12 | 0.36 | 0.19 to 0.54 | <.001 | 57 | -0.09 to 0.82 | 0.047 | 25.48 | 11 | .008 |
| Wellbeing | Post-intervention | Medical condition | 15 | 0.53 | 0.13 to 0.93 | .010 | 91 | -0.99 to 2.05 | 0.558 | 162.31 | 14 | <.001 |
| Wellbeing | Short-term | Healthy | 6 | 0.09 | -0.06 to 0.25 | .246 | 62 | -0.22 to 0.40 | 0.019 | 13.14 | 5 | .022 |
| Wellbeing | Short-term | Mental health condition | 5 | 0.28 | 0.04 to 0.51 | .021 | 52 | -0.16 to 0.71 | 0.036 | 8.38 | 4 | .079 |
| Wellbeing | Short-term | Medical condition | 6 | 0.23 | -0.15 to 0.61 | .240 | 76 | -0.66 to 1.11 | 0.167 | 21.07 | 5 | <.001 |
| Wellbeing | Medium-term | Healthy | 2 | 0.1 | -0.18 to 0.38 | .474 | 65 | -0.33 to 0.53 | 0.028 | 2.87 | 1 | .090 |
| Wellbeing | Medium-term | Mental health condition | 2 | 0.41 | -0.05 to 0.88 | .082 | 40 | -0.23 to 1.05 | 0.051 | 1.66 | 1 | .198 |
| Wellbeing | Medium-term | Medical condition | 3 | -0.03 | -0.38 to 0.31 | .857 | 58 | -0.60 to 0.54 | 0.054 | 4.8 | 2 | .091 |
| Wellbeing | Long-term | Healthy | 1 | 0.17 | -0.09 to 0.42 | .207 | 0 |  | 0 | 0 | 0 |  |
| Wellbeing | Long-term | Mental health condition | 2 | 0.26 | -0.06 to 0.59 | .114 | 0 | -0.06 to 0.59 | 0 | 0.2 | 1 | .658 |
| Depression | Post-intervention | Healthy | 37 | 0.46 | 0.35 to 0.56 | <.001 | 74 | -0.07 to 0.99 | 0.07 | 139.04 | 36 | <.001 |
| Depression | Post-intervention | Mental health condition | 36 | 0.44 | 0.31 to 0.56 | <.001 | 75 | -0.20 to 1.08 | 0.103 | 141.69 | 35 | <.001 |
| Depression | Post-intervention | Medical condition | 37 | 0.42 | 0.24 to 0.60 | <.001 | 82 | -0.55 to 1.40 | 0.239 | 197.39 | 36 | <.001 |
| Depression | Short-term | Healthy | 13 | 0.27 | 0.15 to 0.39 | <.001 | 40 | 0.00 to 0.54 | 0.015 | 20.13 | 12 | .065 |
| Depression | Short-term | Mental health condition | 15 | 0.39 | 0.18 to 0.60 | <.001 | 78 | -0.32 to 1.10 | 0.121 | 64.96 | 14 | <.001 |
| Depression | Short-term | Medical condition | 13 | 0.4 | 0.22 to 0.59 | <.001 | 49 | -0.10 to 0.90 | 0.056 | 23.76 | 12 | .022 |
| Depression | Medium-term | Healthy | 2 | 0.73 | -0.53 to 1.99 | .258 | 97 | -1.44 to 2.90 | 0.81 | 36.39 | 1 | <.001 |
| Depression | Medium-term | Mental health condition | 6 | 0.2 | 0.00 to 0.39 | .049 | 36 | -0.15 to 0.54 | 0.02 | 7.81 | 5 | .167 |
| Depression | Medium-term | Medical condition | 11 | 0.3 | 0.11 to 0.49 | .002 | 53 | -0.18 to 0.77 | 0.05 | 21.13 | 10 | .020 |
| Depression | Long-term | Mental health condition | 3 | 0.29 | -0.03 to 0.62 | .080 | 55 | -0.24 to 0.82 | 0.045 | 4.42 | 2 | .110 |
| Depression | Long-term | Medical condition | 2 | -0.28 | -0.94 to 0.38 | .411 | 68 | -1.30 to 0.74 | 0.157 | 3.17 | 1 | .075 |
| Anxiety | Post-intervention | Healthy | 37 | 0.36 | 0.27 to 0.46 | <.001 | 67 | -0.07 to 0.80 | 0.047 | 107.86 | 36 | <.001 |
| Anxiety | Post-intervention | Mental health condition | 26 | 0.44 | 0.27 to 0.62 | <.001 | 83 | -0.34 to 1.23 | 0.152 | 150.82 | 25 | <.001 |
| Anxiety | Post-intervention | Medical condition | 30 | 0.37 | 0.18 to 0.56 | <.001 | 82 | -0.57 to 1.31 | 0.222 | 157.37 | 29 | <.001 |
| Anxiety | Short-term | Healthy | 14 | 0.24 | 0.13 to 0.35 | <.001 | 32 | 0.01 to 0.47 | 0.01 | 19.06 | 13 | .121 |
| Anxiety | Short-term | Mental health condition | 10 | 0.49 | 0.16 to 0.82 | .004 | 89 | -0.51 to 1.49 | 0.231 | 78.38 | 9 | <.001 |
| Anxiety | Short-term | Medical condition | 12 | 0.4 | 0.14 to 0.66 | .003 | 73 | -0.41 to 1.21 | 0.152 | 40.68 | 11 | <.001 |
| Anxiety | Medium-term | Healthy | 2 | 0.41 | -0.21 to 1.02 | .193 | 89 | -0.62 to 1.44 | 0.177 | 9.4 | 1 | .002 |
| Anxiety | Medium-term | Mental health condition | 4 | 0.06 | -0.15 to 0.28 | .568 | 43 | -0.29 to 0.41 | 0.02 | 5.3 | 3 | .151 |
| Anxiety | Medium-term | Medical condition | 9 | 0.23 | 0.10 to 0.35 | <.001 | 0 | 0.10 to 0.35 | 0 | 5.77 | 8 | .673 |
| Anxiety | Long-term | Mental health condition | 2 | 0.23 | -0.13 to 0.58 | .211 | 64 | -0.31 to 0.76 | 0.043 | 2.79 | 1 | .095 |
| Anxiety | Long-term | Medical condition | 1 | -0.37 | -0.88 to 0.14 | .152 | 0 |  | 0 | 0 | 0 |  |
| Stress | Post-intervention | Healthy | 43 | 0.42 | 0.33 to 0.50 | <.001 | 63 | -0.01 to 0.84 | 0.044 | 113.81 | 42 | <.001 |
| Stress | Post-intervention | Mental health condition | 12 | 0.44 | 0.26 to 0.63 | <.001 | 68 | -0.09 to 0.97 | 0.064 | 34.77 | 11 | <.001 |
| Stress | Post-intervention | Medical condition | 11 | 0.76 | 0.21 to 1.32 | .007 | 94 | -1.09 to 2.61 | 0.81 | 170.96 | 10 | <.001 |
| Stress | Short-term | Healthy | 15 | 0.24 | 0.12 to 0.36 | <.001 | 47 | -0.06 to 0.55 | 0.02 | 26.49 | 14 | .022 |
| Stress | Short-term | Mental health condition | 7 | 0.48 | 0.14 to 0.83 | .006 | 85 | -0.40 to 1.36 | 0.171 | 39.03 | 6 | <.001 |
| Stress | Short-term | Medical condition | 3 | 0.31 | -0.58 to 1.20 | .496 | 93 | -1.42 to 2.03 | 0.571 | 28.14 | 2 | <.001 |
| Stress | Medium-term | Healthy | 5 | 0.29 | 0.11 to 0.47 | .002 | 49 | -0.04 to 0.63 | 0.021 | 7.91 | 4 | .095 |
| Stress | Medium-term | Mental health condition | 1 | 0.71 | 0.04 to 1.39 | .039 | 0 |  | 0 | 0 | 0 |  |
| Stress | Medium-term | Medical condition | 4 | 0.28 | 0.02 to 0.54 | .035 | 35 | -0.12 to 0.68 | 0.024 | 4.6 | 3 | .204 |
| Stress | Long-term | Healthy | 1 | 0.31 | 0.07 to 0.56 | .013 | 0 |  | 0 | 0 | 0 |  |
| Stress | Long-term | Mental health condition | 1 | 0.26 | -0.41 to 0.92 | .453 | 0 |  | 0 | 0 | 0 |  |
| Stress | Long-term | Medical condition | 1 | -0.54 | -1.05 to -0.02 | .040 | 0 |  | 0 | 0 | 0 |  |
| Psychological flexibility | Post-intervention | Healthy | 14 | 0.38 | 0.18 to 0.57 | <.001 | 67 | -0.22 to 0.98 | 0.084 | 39.22 | 13 | <.001 |
| Psychological flexibility | Post-intervention | Mental health condition | 19 | 0.46 | 0.19 to 0.72 | <.001 | 88 | -0.64 to 1.55 | 0.294 | 154.71 | 18 | <.001 |
| Psychological flexibility | Post-intervention | Medical condition | 18 | 0.36 | 0.13 to 0.58 | .002 | 75 | -0.47 to 1.18 | 0.165 | 67.14 | 17 | <.001 |
| Psychological flexibility | Short-term | Healthy | 7 | 0.46 | 0.26 to 0.65 | <.001 | 8 | 0.21 to 0.70 | 0.005 | 6.51 | 6 | .368 |
| Psychological flexibility | Short-term | Mental health condition | 4 | 0.87 | 0.28 to 1.47 | .004 | 89 | -0.38 to 2.13 | 0.319 | 27.56 | 3 | <.001 |
| Psychological flexibility | Short-term | Medical condition | 10 | 0.45 | -0.09 to 0.98 | .101 | 92 | -1.25 to 2.14 | 0.675 | 108.84 | 9 | <.001 |
| Psychological flexibility | Medium-term | Healthy | 1 | 0.08 | -0.22 to 0.38 | .591 | 0 |  | 0 | 0 | 0 |  |
| Psychological flexibility | Medium-term | Mental health condition | 4 | -0.23 | -0.50 to 0.04 | .090 | 38 | -0.66 to 0.19 | 0.028 | 4.85 | 3 | .183 |
| Psychological flexibility | Medium-term | Medical condition | 4 | 0.15 | -0.22 to 0.52 | .422 | 57 | -0.51 to 0.81 | 0.078 | 7.02 | 3 | .071 |
| Psychological flexibility | Long-term | Healthy | 1 | 0.1 | -0.17 to 0.36 | .470 | 0 |  | 0 | 0 | 0 |  |
| Psychological flexibility | Long-term | Mental health condition | 2 | -0.05 | -0.71 to 0.61 | .888 | 67 | -1.07 to 0.97 | 0.158 | 3.07 | 1 | .080 |
| Psychological flexibility | Long-term | Medical condition | 1 | 0.48 | -0.16 to 1.11 | .139 | 0 |  | 0 | 0 | 0 |  |
| Quality of life | Post-intervention | Healthy | 4 | 0.36 | 0.14 to 0.58 | .001 | 18 | 0.07 to 0.65 | 0.009 | 3.66 | 3 | .301 |
| Quality of life | Post-intervention | Mental health condition | 11 | 0.25 | 0.04 to 0.47 | .023 | 69 | -0.36 to 0.87 | 0.086 | 32.54 | 10 | <.001 |
| Quality of life | Post-intervention | Medical condition | 17 | 0.31 | 0.14 to 0.48 | <.001 | 57 | -0.23 to 0.85 | 0.069 | 37.08 | 16 | .002 |
| Quality of life | Short-term | Healthy | 2 | 0.07 | -0.33 to 0.47 | .733 | 0 | -0.33 to 0.47 | 0 | 0.06 | 1 | .806 |
| Quality of life | Short-term | Mental health condition | 3 | 0.14 | -0.21 to 0.49 | .424 | 0 | -0.21 to 0.49 | 0 | 0.44 | 2 | .802 |
| Quality of life | Short-term | Medical condition | 6 | 0.73 | 0.28 to 1.17 | .001 | 81 | -0.33 to 1.79 | 0.242 | 25.71 | 5 | <.001 |
| Quality of life | Medium-term | Healthy | 1 | -0.01 | -0.30 to 0.29 | .972 | 0 |  | 0 | 0 | 0 |  |
| Quality of life | Medium-term | Mental health condition | 1 | -0.01 | -0.48 to 0.46 | .971 | 0 |  | 0 | 0 | 0 |  |
| Quality of life | Medium-term | Medical condition | 5 | 0.11 | -0.17 to 0.40 | .432 | 47 | -0.40 to 0.63 | 0.047 | 7.51 | 4 | .111 |
| Quality of life | Long-term | Healthy | 1 | 0.11 | -0.15 to 0.36 | .413 | 0 |  | 0 | 0 | 0 |  |
| Quality of life | Long-term | Mental health condition | 1 | 0.42 | 0.20 to 0.64 | <.001 | 0 |  | 0 | 0 | 0 |  |
| Quality of life | Long-term | Medical condition | 1 | -0.42 | -0.93 to 0.09 | .105 | 0 |  | 0 | 0 | 0 |  |

**Guidance Subgroups**

Guidance-stratified analyses are presented in Table C. At post-intervention, both guided and unguided interventions demonstrated beneficial effects across most outcomes, with guided formats often associated with moderately larger pooled estimates. This pattern was particularly evident for depression and anxiety at short-term follow-up. However, unguided interventions also showed clinically meaningful effects in several domains, indicating that therapist support was not a necessary condition for benefit. Differences between guidance formats were not uniform across timepoints or outcomes, and confidence intervals frequently overlapped.

Table C.

| Outcome | Timepoint | Subgroup Level | k | Effect (g) | 95% CI | p | I² (%) | Prediction interval | τ² | Q | df(Q) | p(Q) |
| --- | --- | --- | --- | --- | --- | --- | --- | --- | --- | --- | --- | --- |
| Wellbeing | Post-intervention | Guided | 25 | 0.49 | 0.27 to 0.72 | <.001 | 87 | -0.56 to 1.55 | 0.278 | 181.76 | 24 | <.001 |
| Wellbeing | Post-intervention | Unguided | 21 | 0.28 | 0.16 to 0.41 | <.001 | 71 | -0.17 to 0.73 | 0.048 | 68.89 | 20 | <.001 |
| Wellbeing | Short-term | Guided | 6 | 0.38 | 0.09 to 0.68 | .012 | 67 | -0.27 to 1.04 | 0.089 | 15.22 | 5 | .009 |
| Wellbeing | Short-term | Unguided | 11 | 0.09 | -0.04 to 0.23 | .168 | 58 | -0.23 to 0.42 | 0.023 | 23.7 | 10 | .008 |
| Wellbeing | Medium-term | Guided | 6 | 0.16 | -0.07 to 0.39 | .163 | 55 | -0.30 to 0.62 | 0.041 | 11.18 | 5 | .048 |
| Wellbeing | Medium-term | Unguided | 1 | -0.02 | -0.17 to 0.14 | .852 | 0 | -0.17 to 0.14 | 0 | 0 | 0 |  |
| Wellbeing | Long-term | Guided | 3 | 0.2 | 0.00 to 0.41 | .049 | 0 | 0.00 to 0.41 | 0 | 0.4 | 2 | .819 |
| Depression | Post-intervention | Guided | 65 | 0.47 | 0.35 to 0.58 | <.001 | 80 | -0.32 to 1.25 | 0.159 | 322.61 | 64 | <.001 |
| Depression | Post-intervention | Unguided | 45 | 0.42 | 0.33 to 0.52 | <.001 | 73 | -0.11 to 0.95 | 0.071 | 165.82 | 44 | <.001 |
| Depression | Short-term | Guided | 20 | 0.49 | 0.34 to 0.64 | <.001 | 54 | 0.01 to 0.97 | 0.055 | 41.23 | 19 | .002 |
| Depression | Short-term | Unguided | 21 | 0.28 | 0.14 to 0.42 | <.001 | 72 | -0.21 to 0.78 | 0.059 | 71.39 | 20 | <.001 |
| Depression | Medium-term | Guided | 17 | 0.34 | 0.15 to 0.52 | <.001 | 74 | -0.31 to 0.98 | 0.1 | 61.23 | 16 | <.001 |
| Depression | Medium-term | Unguided | 2 | 0.08 | -0.07 to 0.24 | .282 | 0 | -0.07 to 0.24 | 0 | 0.41 | 1 | .522 |
| Depression | Long-term | Guided | 5 | 0.08 | -0.30 to 0.46 | .670 | 75 | -0.73 to 0.90 | 0.134 | 16.2 | 4 | .003 |
| Anxiety | Post-intervention | Guided | 61 | 0.42 | 0.31 to 0.53 | <.001 | 80 | -0.32 to 1.16 | 0.14 | 297.11 | 60 | <.001 |
| Anxiety | Post-intervention | Unguided | 32 | 0.36 | 0.25 to 0.48 | <.001 | 77 | -0.19 to 0.92 | 0.076 | 134.65 | 31 | <.001 |
| Anxiety | Short-term | Guided | 20 | 0.48 | 0.32 to 0.64 | <.001 | 62 | -0.07 to 1.03 | 0.073 | 49.54 | 19 | <.001 |
| Anxiety | Short-term | Unguided | 16 | 0.3 | 0.10 to 0.51 | .004 | 87 | -0.44 to 1.05 | 0.134 | 114.57 | 15 | <.001 |
| Anxiety | Medium-term | Guided | 14 | 0.25 | 0.14 to 0.37 | <.001 | 33 | -0.01 to 0.52 | 0.015 | 19.4 | 13 | .111 |
| Anxiety | Medium-term | Unguided | 1 | 0.12 | -0.04 to 0.28 | .151 | 0 | -0.04 to 0.28 | 0 | 0 | 0 |  |
| Anxiety | Long-term | Guided | 3 | 0.05 | -0.36 to 0.47 | .800 | 76 | -0.70 to 0.81 | 0.101 | 8.4 | 2 | .015 |
| Stress | Post-intervention | Guided | 35 | 0.5 | 0.33 to 0.68 | <.001 | 85 | -0.45 to 1.45 | 0.225 | 222.71 | 34 | <.001 |
| Stress | Post-intervention | Unguided | 31 | 0.43 | 0.33 to 0.53 | <.001 | 69 | -0.01 to 0.86 | 0.047 | 97.71 | 30 | <.001 |
| Stress | Short-term | Guided | 13 | 0.43 | 0.15 to 0.72 | .003 | 79 | -0.51 to 1.37 | 0.208 | 55.83 | 12 | <.001 |
| Stress | Short-term | Unguided | 12 | 0.26 | 0.10 to 0.43 | .002 | 79 | -0.23 to 0.76 | 0.056 | 51.98 | 11 | <.001 |
| Stress | Medium-term | Guided | 8 | 0.37 | 0.21 to 0.52 | <.001 | 21 | 0.11 to 0.62 | 0.011 | 8.91 | 7 | .259 |
| Stress | Medium-term | Unguided | 2 | 0.12 | -0.03 to 0.26 | .125 | 0 | -0.03 to 0.26 | 0 | 0.06 | 1 | .801 |
| Stress | Long-term | Guided | 3 | 0.02 | -0.53 to 0.58 | .931 | 77 | -0.98 to 1.02 | 0.18 | 8.67 | 2 | .013 |
| Psychological flexibility | Post-intervention | Guided | 33 | 0.34 | 0.17 to 0.51 | <.001 | 80 | -0.53 to 1.21 | 0.188 | 160.03 | 32 | <.001 |
| Psychological flexibility | Post-intervention | Unguided | 18 | 0.52 | 0.32 to 0.73 | <.001 | 80 | -0.26 to 1.31 | 0.149 | 86.26 | 17 | <.001 |
| Psychological flexibility | Short-term | Guided | 15 | 0.38 | 0.09 to 0.67 | .010 | 81 | -0.66 to 1.42 | 0.258 | 74.68 | 14 | <.001 |
| Psychological flexibility | Short-term | Unguided | 6 | 0.96 | 0.33 to 1.59 | .003 | 92 | -0.62 to 2.54 | 0.55 | 66.63 | 5 | <.001 |
| Psychological flexibility | Medium-term | Guided | 9 | 0.05 | -0.17 to 0.26 | .680 | 63 | -0.49 to 0.58 | 0.063 | 21.41 | 8 | .006 |
| Psychological flexibility | Long-term | Guided | 4 | 0.08 | -0.25 to 0.41 | .648 | 55 | -0.51 to 0.66 | 0.06 | 6.68 | 3 | .083 |
| Quality of life | Post-intervention | Guided | 25 | 0.27 | 0.14 to 0.39 | <.001 | 53 | -0.18 to 0.72 | 0.048 | 51.01 | 24 | .001 |
| Quality of life | Post-intervention | Unguided | 7 | 0.42 | 0.11 to 0.73 | .008 | 72 | -0.33 to 1.17 | 0.12 | 21.2 | 6 | .002 |
| Quality of life | Short-term | Guided | 8 | 0.48 | 0.10 to 0.86 | .013 | 76 | -0.52 to 1.47 | 0.22 | 29.55 | 7 | <.001 |
| Quality of life | Short-term | Unguided | 3 | 0.42 | -0.25 to 1.09 | .220 | 78 | -0.81 to 1.65 | 0.276 | 9.24 | 2 | .010 |
| Quality of life | Medium-term | Guided | 7 | 0.08 | -0.11 to 0.26 | .409 | 28 | -0.23 to 0.39 | 0.017 | 8.29 | 6 | .218 |
| Quality of life | Long-term | Guided | 3 | 0.09 | -0.29 to 0.48 | .635 | 80 | -0.61 to 0.80 | 0.091 | 9.99 | 2 | .007 |

**Intervention-Family Subgroups**

Intervention-family analyses are presented in Table D. Acceptance and Commitment Therapy (ACT) was the most frequently represented intervention type and demonstrated consistent effects across outcomes at post-intervention. Mindfulness-based interventions (MBI/MBCT) also showed beneficial effects in several domains, although estimates were derived from fewer trials in some strata. Estimates for less represented approaches (e.g., DBT-informed or compassion-focused digital programmes) were based on small numbers of studies and exhibited wider confidence intervals. Accordingly, these results should be interpreted cautiously as exploratory comparisons rather than definitive evidence of superiority among third-wave approaches.

Table D.

| Outcome | Timepoint | Subgroup Level | k | Effect (g) | 95% CI | p | I² (%) | Prediction interval | τ² | Q | df(Q) | p(Q) |
| --- | --- | --- | --- | --- | --- | --- | --- | --- | --- | --- | --- | --- |
| Wellbeing | Post-intervention | ACT | 20 | 0.34 | 0.23 to 0.45 | <.001 | 35 | 0.03 to 0.64 | 0.021 | 29.4 | 19 | .060 |
| Wellbeing | Post-intervention | CFT | 4 | 2.35 | 0.67 to 4.04 | .006 | 98 | -1.35 to 6.06 | 2.829 | 175.75 | 3 | <.001 |
| Wellbeing | Post-intervention | DBT | 3 | 0.18 | -0.13 to 0.50 | .254 | 46 | -0.31 to 0.68 | 0.038 | 3.72 | 2 | .156 |
| Wellbeing | Post-intervention | MBCT | 5 | 0.21 | 0.12 to 0.31 | <.001 | 0 | 0.12 to 0.31 | 0 | 0.55 | 4 | .969 |
| Wellbeing | Post-intervention | MBI | 10 | 0.26 | 0.05 to 0.46 | .015 | 71 | -0.30 to 0.81 | 0.068 | 30.55 | 9 | <.001 |
| Wellbeing | Post-intervention | MBSR | 3 | 0.34 | -0.24 to 0.92 | .254 | 73 | -0.70 to 1.38 | 0.194 | 7.43 | 2 | .024 |
| Wellbeing | Post-intervention | Mindfulness–CBT integrative programmes | 1 | 0.26 | -0.38 to 0.90 | .433 | 0 |  | 0 | 0 | 0 |  |
| Wellbeing | Short-term | ACT | 5 | 0.36 | 0.14 to 0.57 | .001 | 36 | -0.01 to 0.72 | 0.022 | 6.26 | 4 | .180 |
| Wellbeing | Short-term | CFT | 2 | 0.35 | -0.64 to 1.33 | .492 | 91 | -1.31 to 2.00 | 0.46 | 10.65 | 1 | .001 |
| Wellbeing | Short-term | DBT | 1 | -0.07 | -0.35 to 0.21 | .610 | 0 |  | 0 | 0 | 0 |  |
| Wellbeing | Short-term | MBCT | 2 | 0.01 | -0.12 to 0.14 | .869 | 0 | -0.12 to 0.14 | 0 | 0.01 | 1 | .934 |
| Wellbeing | Short-term | MBI | 4 | 0.13 | -0.09 to 0.36 | .251 | 54 | -0.26 to 0.53 | 0.027 | 6.59 | 3 | .086 |
| Wellbeing | Short-term | MBSR | 2 | 0.36 | -0.90 to 1.63 | .574 | 90 | -1.75 to 2.48 | 0.749 | 10.11 | 1 | .001 |
| Wellbeing | Short-term | Mindfulness–CBT integrative programmes | 1 | 0.56 | -0.12 to 1.25 | .107 | 0 |  | 0 | 0 | 0 |  |
| Wellbeing | Medium-term | ACT | 4 | 0.24 | 0.06 to 0.42 | .010 | 4 | 0.04 to 0.44 | 0.002 | 3.13 | 3 | .371 |
| Wellbeing | Medium-term | DBT | 1 | 0.21 | -0.25 to 0.67 | .366 | 0 |  | 0 | 0 | 0 |  |
| Wellbeing | Medium-term | MBCT | 2 | -0.16 | -0.51 to 0.20 | .389 | 66 | -0.71 to 0.40 | 0.047 | 2.96 | 1 | .086 |
| Wellbeing | Long-term | ACT | 3 | 0.2 | 0.00 to 0.40 | .049 | 0 | 0.00 to 0.40 | 0 | 0.4 | 2 | .819 |
| Depression | Post-intervention | ACT | 53 | 0.4 | 0.31 to 0.50 | <.001 | 63 | -0.13 to 0.94 | 0.071 | 141.02 | 52 | <.001 |
| Depression | Post-intervention | CFT | 1 | 6.44 | 5.39 to 7.48 | <.001 | 0 |  | 0 | 0 | 0 |  |
| Depression | Post-intervention | DBT | 5 | 0.33 | 0.08 to 0.58 | .010 | 43 | -0.11 to 0.77 | 0.033 | 6.96 | 4 | .138 |
| Depression | Post-intervention | MBCT | 7 | 0.73 | 0.40 to 1.07 | <.001 | 88 | -0.13 to 1.59 | 0.163 | 49.73 | 6 | <.001 |
| Depression | Post-intervention | MBI | 31 | 0.43 | 0.30 to 0.57 | <.001 | 75 | -0.18 to 1.05 | 0.095 | 122.11 | 30 | <.001 |
| Depression | Post-intervention | MBSR | 6 | 0.12 | -0.09 to 0.33 | .249 | 31 | -0.23 to 0.47 | 0.02 | 7.22 | 5 | .205 |
| Depression | Post-intervention | Mindfulness–CBT integrative programmes | 1 | 0.31 | -0.01 to 0.62 | .058 | 0 |  | 0 | 0 | 0 |  |
| Depression | Post-intervention | Mindfulness–CBT integrative programmes | 6 | 0.34 | 0.09 to 0.58 | .006 | 67 | -0.20 to 0.87 | 0.058 | 15.25 | 5 | .009 |
| Depression | Short-term | ACT | 19 | 0.4 | 0.27 to 0.52 | <.001 | 43 | 0.02 to 0.77 | 0.032 | 31.31 | 18 | .026 |
| Depression | Short-term | DBT | 1 | -0.02 | -0.30 to 0.26 | .873 | 0 |  | 0 | 0 | 0 |  |
| Depression | Short-term | MBCT | 3 | 0.4 | 0.06 to 0.74 | .022 | 84 | -0.23 to 1.03 | 0.073 | 12.77 | 2 | .002 |
| Depression | Short-term | MBI | 14 | 0.44 | 0.21 to 0.67 | <.001 | 80 | -0.32 to 1.20 | 0.138 | 66.32 | 13 | <.001 |
| Depression | Short-term | MBSR | 2 | 0.1 | -0.46 to 0.67 | .717 | 50 | -0.70 to 0.90 | 0.084 | 2.02 | 1 | .156 |
| Depression | Short-term | Mindfulness–CBT integrative programmes | 2 | 0.24 | -0.10 to 0.59 | .170 | 0 | -0.10 to 0.59 | 0 | 0.05 | 1 | .829 |
| Depression | Medium-term | ACT | 9 | 0.29 | 0.06 to 0.52 | .013 | 62 | -0.27 to 0.85 | 0.068 | 20.87 | 8 | .008 |
| Depression | Medium-term | DBT | 1 | 0.47 | 0.00 to 0.93 | .049 | 0 |  | 0 | 0 | 0 |  |
| Depression | Medium-term | MBCT | 5 | 0.44 | 0.04 to 0.84 | .029 | 90 | -0.47 to 1.36 | 0.175 | 38.25 | 4 | <.001 |
| Depression | Medium-term | MBI | 2 | 0.28 | -0.40 to 0.95 | .426 | 65 | -0.75 to 1.30 | 0.156 | 2.82 | 1 | .093 |
| Depression | Medium-term | Mindfulness–CBT integrative programmes | 2 | 0.04 | -0.23 to 0.30 | .784 | 0 | -0.23 to 0.30 | 0 | 0.27 | 1 | .606 |
| Depression | Long-term | ACT | 3 | -0.07 | -0.62 to 0.48 | .807 | 72 | -1.05 to 0.91 | 0.17 | 7.12 | 2 | .028 |
| Depression | Long-term | MBCT | 2 | 0.34 | 0.02 to 0.66 | .040 | 35 | -0.11 to 0.78 | 0.024 | 1.54 | 1 | .215 |
| Anxiety | Post-intervention | ACT | 45 | 0.38 | 0.27 to 0.49 | <.001 | 69 | -0.21 to 0.98 | 0.089 | 143.91 | 44 | <.001 |
| Anxiety | Post-intervention | CFT | 1 | 5.49 | 4.57 to 6.41 | <.001 | 0 |  | 0 | 0 | 0 |  |
| Anxiety | Post-intervention | DBT | 4 | 0.17 | -0.01 to 0.34 | .060 | 8 | -0.04 to 0.38 | 0.004 | 3.27 | 3 | .352 |
| Anxiety | Post-intervention | MBCT | 4 | 0.39 | 0.20 to 0.58 | <.001 | 58 | 0.05 to 0.73 | 0.02 | 7.11 | 3 | .068 |
| Anxiety | Post-intervention | MBI | 27 | 0.39 | 0.26 to 0.52 | <.001 | 74 | -0.17 to 0.95 | 0.077 | 99.52 | 26 | <.001 |
| Anxiety | Post-intervention | MBSR | 7 | 0.09 | -0.16 to 0.34 | .472 | 61 | -0.47 to 0.65 | 0.065 | 15.2 | 6 | .019 |
| Anxiety | Post-intervention | Mindfulness–CBT integrative programmes | 4 | 0.28 | -0.08 to 0.64 | .123 | 73 | -0.41 to 0.97 | 0.092 | 10.93 | 3 | .012 |
| Anxiety | Post-intervention | Mindfulness–CBT integrative programmes | 1 | 0.51 | 0.20 to 0.83 | .002 | 0 |  | 0 | 0 | 0 |  |
| Anxiety | Short-term | ACT | 17 | 0.38 | 0.16 to 0.61 | .001 | 80 | -0.47 to 1.24 | 0.177 | 79.42 | 16 | <.001 |
| Anxiety | Short-term | DBT | 1 | 0.11 | -0.17 to 0.39 | .452 | 0 |  | 0 | 0 | 0 |  |
| Anxiety | Short-term | MBCT | 3 | 0.29 | 0.00 to 0.57 | .049 | 78 | -0.22 to 0.80 | 0.046 | 8.94 | 2 | .011 |
| Anxiety | Short-term | MBI | 12 | 0.45 | 0.22 to 0.68 | <.001 | 79 | -0.24 to 1.14 | 0.111 | 52.19 | 11 | <.001 |
| Anxiety | Short-term | MBSR | 2 | 0.17 | -0.78 to 1.13 | .721 | 82 | -1.38 to 1.72 | 0.389 | 5.59 | 1 | .018 |
| Anxiety | Short-term | Mindfulness–CBT integrative programmes | 1 | 0.87 | 0.01 to 1.73 | .046 | 0 |  | 0 | 0 | 0 |  |
| Anxiety | Medium-term | ACT | 7 | 0.11 | -0.06 to 0.29 | .195 | 36 | -0.21 to 0.44 | 0.019 | 9.32 | 6 | .156 |
| Anxiety | Medium-term | DBT | 1 | 0.12 | -0.33 to 0.58 | .598 | 0 |  | 0 | 0 | 0 |  |
| Anxiety | Medium-term | MBCT | 3 | 0.2 | 0.06 to 0.34 | .004 | 16 | 0.03 to 0.38 | 0.003 | 2.39 | 2 | .302 |
| Anxiety | Medium-term | MBI | 3 | 0.38 | -0.12 to 0.87 | .134 | 68 | -0.48 to 1.23 | 0.127 | 6.18 | 2 | .046 |
| Anxiety | Medium-term | Mindfulness–CBT integrative programmes | 1 | 0.14 | -0.16 to 0.44 | .367 | 0 |  | 0 | 0 | 0 |  |
| Anxiety | Long-term | ACT | 2 | -0.14 | -0.50 to 0.23 | .457 | 30 | -0.60 to 0.33 | 0.022 | 1.42 | 1 | .233 |
| Anxiety | Long-term | MBCT | 1 | 0.38 | 0.16 to 0.59 | <.001 | 0 |  | 0 | 0 | 0 |  |
| Stress | Post-intervention | ACT | 22 | 0.34 | 0.23 to 0.45 | <.001 | 33 | 0.04 to 0.64 | 0.02 | 31.22 | 21 | .070 |
| Stress | Post-intervention | CFT | 2 | 3.53 | -3.27 to 10.32 | .309 | 99 | -8.22 to 15.27 | 23.888 | 141.15 | 1 | <.001 |
| Stress | Post-intervention | DBT | 3 | 0.19 | -0.08 to 0.46 | .170 | 48 | -0.24 to 0.61 | 0.028 | 3.84 | 2 | .147 |
| Stress | Post-intervention | MBCT | 4 | 0.47 | 0.04 to 0.90 | .031 | 87 | -0.42 to 1.37 | 0.161 | 23.5 | 3 | <.001 |
| Stress | Post-intervention | MBI | 26 | 0.47 | 0.36 to 0.58 | <.001 | 55 | 0.08 to 0.86 | 0.037 | 55.67 | 25 | <.001 |
| Stress | Post-intervention | MBSR | 4 | 0.57 | -0.02 to 1.16 | .057 | 89 | -0.68 to 1.82 | 0.315 | 27.19 | 3 | <.001 |
| Stress | Post-intervention | Mindfulness–CFT integrative programmes | 3 | 0.37 | -0.16 to 0.89 | .176 | 87 | -0.64 to 1.37 | 0.191 | 15.91 | 2 | <.001 |
| Stress | Post-intervention | Mindfulness–CBT integrative programmes | 2 | 0.57 | 0.31 to 0.83 | <.001 | 0 | 0.31 to 0.83 | 0 | 0.13 | 1 | .720 |
| Stress | Short-term | ACT | 11 | 0.26 | 0.02 to 0.50 | .035 | 69 | -0.42 to 0.94 | 0.105 | 32.5 | 10 | <.001 |
| Stress | Short-term | CFT | 1 | 0.04 | -0.19 to 0.26 | .762 | 0 |  | 0 | 0 | 0 |  |
| Stress | Short-term | DBT | 1 | 0.02 | -0.26 to 0.30 | .898 | 0 |  | 0 | 0 | 0 |  |
| Stress | Short-term | MBCT | 2 | 0.36 | -0.39 to 1.11 | .349 | 91 | -0.90 to 1.62 | 0.267 | 10.97 | 1 | <.001 |
| Stress | Short-term | MBI | 8 | 0.45 | 0.16 to 0.73 | .002 | 82 | -0.31 to 1.20 | 0.127 | 39.81 | 7 | <.001 |
| Stress | Short-term | MBSR | 2 | 0.97 | 0.41 to 1.54 | <.001 | 55 | 0.15 to 1.80 | 0.094 | 2.23 | 1 | .135 |
| Stress | Medium-term | ACT | 3 | 0.49 | 0.15 to 0.82 | .004 | 33 | 0.00 to 0.97 | 0.032 | 2.96 | 2 | .227 |
| Stress | Medium-term | DBT | 1 | 0.27 | -0.19 to 0.73 | .253 | 0 |  | 0 | 0 | 0 |  |
| Stress | Medium-term | MBCT | 3 | 0.26 | 0.02 to 0.51 | .037 | 53 | -0.14 to 0.66 | 0.026 | 4.29 | 2 | .117 |
| Stress | Medium-term | MBI | 2 | 0.37 | -0.02 to 0.76 | .061 | 52 | -0.18 to 0.93 | 0.041 | 2.08 | 1 | .150 |
| Stress | Medium-term | Mindfulness–CBT integrative programmes | 1 | 0.06 | -0.24 to 0.36 | .698 | 0 |  | 0 | 0 | 0 |  |
| Stress | Long-term | ACT | 3 | 0.02 | -0.53 to 0.58 | .931 | 77 | -0.98 to 1.02 | 0.18 | 8.67 | 2 | .013 |
| Psychological flexibility | Post-intervention | ACT | 47 | 0.38 | 0.24 to 0.52 | <.001 | 81 | -0.48 to 1.23 | 0.186 | 245.96 | 46 | <.001 |
| Psychological flexibility | Post-intervention | MBCT | 1 | 0.97 | 0.52 to 1.41 | <.001 | 0 |  | 0 | 0 | 0 |  |
| Psychological flexibility | Post-intervention | MBI | 2 | 0.43 | -0.83 to 1.69 | .502 | 94 | -1.71 to 2.57 | 0.779 | 16.31 | 1 | <.001 |
| Psychological flexibility | Post-intervention | MBSR | 1 | 0.64 | 0.17 to 1.10 | .007 | 0 |  | 0 | 0 | 0 |  |
| Psychological flexibility | Short-term | ACT | 20 | 0.53 | 0.23 to 0.83 | <.001 | 89 | -0.75 to 1.81 | 0.403 | 169.45 | 19 | <.001 |
| Psychological flexibility | Short-term | MBCT | 1 | 0.75 | 0.31 to 1.18 | <.001 | 0 |  | 0 | 0 | 0 |  |
| Psychological flexibility | Medium-term | ACT | 8 | -0.01 | -0.27 to 0.26 | .965 | 70 | -0.67 to 0.66 | 0.097 | 23.05 | 7 | .002 |
| Psychological flexibility | Medium-term | MBI | 1 | -0.1 | -0.57 to 0.37 | .684 | 0 |  | 0 | 0 | 0 |  |
| Psychological flexibility | Long-term | ACT | 4 | 0.08 | -0.25 to 0.41 | .648 | 55 | -0.50 to 0.66 | 0.06 | 6.68 | 3 | .083 |
| Quality of life | Post-intervention | ACT | 22 | 0.3 | 0.14 to 0.45 | <.001 | 63 | -0.28 to 0.87 | 0.08 | 56.05 | 21 | <.001 |
| Quality of life | Post-intervention | DBT | 1 | 0.19 | -0.53 to 0.91 | .610 | 0 |  | 0 | 0 | 0 |  |
| Quality of life | Post-intervention | MBCT | 1 | 0.56 | 0.34 to 0.77 | <.001 | 0 |  | 0 | 0 | 0 |  |
| Quality of life | Post-intervention | MBI | 5 | 0.21 | -0.03 to 0.46 | .091 | 17 | -0.12 to 0.54 | 0.013 | 4.79 | 4 | .309 |
| Quality of life | Post-intervention | MBSR | 1 | 0.66 | 0.27 to 1.06 | <.001 | 0 |  | 0 | 0 | 0 |  |
| Quality of life | Post-intervention | Mindfulness–CBT integrative programmes | 2 | 0.24 | -0.02 to 0.49 | .068 | 28 | -0.08 to 0.56 | 0.01 | 1.39 | 1 | .239 |
| Quality of life | Short-term | ACT | 8 | 0.34 | 0.09 to 0.60 | .009 | 49 | -0.22 to 0.91 | 0.066 | 13.82 | 7 | .055 |
| Quality of life | Short-term | MBI | 2 | 0.38 | -0.19 to 0.95 | .187 | 36 | -0.37 to 1.14 | 0.063 | 1.56 | 1 | .212 |
| Quality of life | Short-term | MBSR | 1 | 1.5 | 1.07 to 1.94 | <.001 | 0 |  | 0 | 0 | 0 |  |
| Quality of life | Medium-term | ACT | 5 | 0.07 | -0.17 to 0.30 | .581 | 47 | -0.36 to 0.49 | 0.032 | 7.58 | 4 | .108 |
| Quality of life | Medium-term | MBI | 2 | 0.09 | -0.31 to 0.50 | .654 | 0 | -0.31 to 0.50 | 0 | 0.7 | 1 | .401 |
| Quality of life | Long-term | ACT | 2 | -0.11 | -0.62 to 0.40 | .675 | 70 | -0.90 to 0.68 | 0.097 | 3.29 | 1 | .070 |
| Quality of life | Long-term | MBCT | 1 | 0.42 | 0.20 to 0.64 | <.001 | 0 |  | 0 | 0 | 0 |  |
