# Supplementary material for: Impacts of digital third-wave interventions for adults on wellbeing and mental health outcomes: A systematic review and meta-analysis of randomized controlled trials
Source: PLOS Digit Health. 2026 Oct 1;5(10):e0001764. doi: 10.1371/journal.pdig.0001764 (PMC13630215; doi:10.1371/journal.pdig.0001764)
Supplement: Supplementary file 8 [file pdig.0001764.s008.docx]

**S8 Appendix. Meta-regression results.**

Text A.

Random-effects meta-regression models (Method of Moments/DerSimonian-Laird) were used to investigate categorical moderators for all primary outcomes at post-intervention and short-term follow-up. Four moderators were evaluated: intervention family (ACT, MBCT, CFT, etc.), population type (healthy, medical, or mental health condition), therapist involvement (guided vs. unguided), and comparator type (active vs. inactive). To address dependencies in multi-arm trials, a Split-N correction was applied to the shared intervention group by dividing its sample size across relevant comparisons before calculating effect size variance. This ensured that participants were not double-counted and that the precision of these trials was not artificially inflated.

The analysis confirmed that comparator type was a robust predictor of efficacy; inactive controls yielded significantly larger effects than active controls across several outcomes, including post-intervention depression (p = .003) and short-term stress (p < .001). Population type was non-significant for all outcomes at post-intervention (ps > .35), providing statistical validation for the transdiagnostic applicability of these interventions. While therapist involvement did not predict post-intervention outcomes, it significantly predicted the maintenance of depression gains at short-term follow-up (p = .048), highlighting the role of human support in durability.

Table A.

| Outcome | Timepoint | Moderator | k | QM | df | p-value | Tau² |
| --- | --- | --- | --- | --- | --- | --- | --- |
| Wellbeing | Post | Intervention Type | 46 | 6.02 | 6 | .437 | 0.127 |
| Wellbeing | Post | Population Type | 46 | 0.04 | 2 | .979 | 0.127 |
| Wellbeing | Post | Therapist Involvement | 46 | 0.56 | 1 | .460 | 0.127 |
| Wellbeing | Post | Comparator Type | 50 | 1.15 | 1 | .290 | 0.125 |
| Wellbeing | Short-term | Comparator Type | 18 | 5.16 | 1 | .037 | 0.040 |
| Depression | Post | Intervention Type | 110 | 126.12 | 7 | <.001 | 0.112 |
| Depression | Post | Population Type | 110 | 0.43 | 2 | .806 | 0.112 |
| Depression | Post | Therapist Involvement | 110 | 0.13 | 1 | .719 | 0.112 |
| Depression | Post | Comparator Type | 119 | 9.42 | 1 | .003 | 0.111 |
| Depression | Short-term | Therapist Involvement | 41 | 4.17 | 1 | .048 | 0.066 |
| Depression | Short-term | Comparator Type | 43 | 4.45 | 1 | .041 | 0.065 |
| Anxiety | Post | Intervention Type | 93 | 106.30 | 7 | <.001 | 0.109 |
| Anxiety | Post | Population Type | 93 | 0.76 | 2 | .686 | 0.109 |
| Anxiety | Post | Therapist Involvement | 93 | 0.21 | 1 | .646 | 0.109 |
| Anxiety | Post | Comparator Type | 103 | 9.96 | 1 | .002 | 0.108 |
| Anxiety | Short-term | Comparator Type | 38 | 3.67 | 1 | .063 | 0.108 |
| Stress | Post | Intervention Type | 66 | 8.00 | 7 | .350 | 0.111 |
| Stress | Post | Population Type | 66 | 0.30 | 2 | .863 | 0.111 |
| Stress | Post | Therapist Involvement | 66 | 0.08 | 1 | .777 | 0.111 |
| Stress | Post | Comparator Type | 73 | 2.33 | 1 | .132 | 0.109 |
| Stress | Short-term | Comparator Type | 27 | 14.03 | 1 | <.001 | 0.097 |
| Psych Flex | Post | Comparator Type | 54 | 5.37 | 1 | .021 | 0.185 |
| Psych Flex | Post | Therapist Involvement | 51 | 1.44 | 1 | .236 | 0.184 |
| Qual. of Life | Post | Therapist Involvement | 32 | 1.00 | 1 | .324 | 0.060 |
| Qual. of Life | Short-term | Intervention Type | 11 | 9.77 | 2 | .041 | 0.201 |
