# Supplementary material for: Impacts of digital third-wave interventions for adults on wellbeing and mental health outcomes: A systematic review and meta-analysis of randomized controlled trials
Source: PLOS Digit Health. 2026 Oct 1;5(10):e0001764. doi: 10.1371/journal.pdig.0001764 (PMC13630215; doi:10.1371/journal.pdig.0001764)
Supplement: Supplementary file 9 [file pdig.0001764.s009.docx]

**S9 Appendix. High-risk-of-bias exclusion sensitivity analysis.**

Text A.

We conducted sensitivity analyses to assess the robustness of the primary meta-analyses by repeating each pooled analysis after excluding results judged to be at overall high risk of bias using RoB 2. This sensitivity analysis was undertaken to evaluate whether the main findings were dependent on studies with serious methodological limitations and to inform interpretation of robustness and certainty of evidence.

For each outcome and time window (post-intervention; short-term ≤3 months; medium-term >3–6 months; long-term >6 months), we excluded any study contribution where the study’s overall RoB 2 judgement was rated “High”. All other analytic decisions were held constant, including the use of Hedges’ *g* as the effect size metric, direction-normalisation so that positive values consistently indicated improvement, the handling of multi-arm trials and unit-of-analysis constraints, and the use of random-effects DerSimonian–Laird models. Results are reported as pooled Hedges’ *g* with 95% confidence intervals; heterogeneity statistics and 95% prediction intervals are also presented.

Across the 24 planned primary meta-analyses (six outcomes across up to four time windows), eight analyses contained no studies rated at overall high risk of bias and therefore yielded identical pooled estimates to the corresponding primary analyses by definition. These included all long-term follow-up analyses for anxiety, depression, stress, wellbeing and quality of life, as well as medium-term analyses for stress, wellbeing and psychological flexibility. In the remaining analyses, exclusion of high risk-of-bias results reduced the number of contributing studies but generally produced pooled effects that were similar in magnitude and direction to the primary analyses.

One exception was wellbeing at short-term follow-up, where exclusion of high risk-of-bias results reduced the pooled estimate from *g =* 0.18 to *g =* 0.14 and widened the confidence interval, with the *p value* changing from .006 to .051. For quality of life at medium-term follow-up, the estimate was reduced from *g =* 0.08 to *g =* 0.05 but was not statistically significant in either the primary or sensitivity analysis. In all other outcome–time window combinations, exclusion of high risk-of-bias results did not materially alter the substantive conclusions of the review, and effect direction and overall patterns of statistically supported results were stable.

Table A. Random-effects pooled estimates after exclusion of high risk-of-bias results for each outcome and time window, alongside the corresponding number of included studies, the number of excluded studies, heterogeneity statistics and 95% prediction intervals.

| **Outcome** | **Timepoint** | **K** | **Excluded (High RoB)** | **g (95% CI)** | **p** | **I² (%)** | **95% PI** |
| --- | --- | --- | --- | --- | --- | --- | --- |
| Anxiety | Long-term | 3 | 0 | 0.05 (−0.36 to 0.47) | .800 | 76 | −0.70 to 0.80 |
| Anxiety | Medium-term | 14 | 1 | 0.22 (0.11 to 0.34) | <.001 | 37 | −0.05 to 0.49 |
| Anxiety | Post-intervention | 79 | 14 | 0.42 (0.33 to 0.51) | <.001 | 80 | −0.27 to 1.11 |
| Anxiety | Short-term | 31 | 5 | 0.39 (0.25 to 0.54) | <.001 | 82 | −0.31 to 1.09 |
| Depression | Long-term | 5 | 0 | 0.08 (−0.30 to 0.46) | .670 | 75 | −0.73 to 0.90 |
| Depression | Medium-term | 18 | 1 | 0.29 (0.11 to 0.46) | .001 | 74 | −0.33 to 0.91 |
| Depression | Post-intervention | 94 | 16 | 0.47 (0.38 to 0.55) | <.001 | 79 | −0.24 to 1.17 |
| Depression | Short-term | 36 | 5 | 0.38 (0.27 to 0.49) | <.001 | 67 | −0.11 to 0.87 |
| Psychological flexibility | Long-term | 3 | 1 | −0.01 (−0.36 to 0.34) | .962 | 57 | −0.58 to 0.57 |
| Psychological flexibility | Medium-term | 9 | 0 | 0.05 (−0.17 to 0.26) | .680 | 63 | −0.49 to 0.58 |
| Psychological flexibility | Post-intervention | 45 | 6 | 0.39 (0.24 to 0.54) | <.001 | 83 | −0.49 to 1.28 |
| Psychological flexibility | Short-term | 17 | 4 | 0.53 (0.18 to 0.87) | .003 | 91 | −0.85 to 1.90 |
| Quality of life | Long-term | 3 | 0 | 0.09 (−0.30 to 0.48) | .635 | 80 | −0.61 to 0.80 |
| Quality of life | Medium-term | 6 | 1 | 0.05 (−0.20 to 0.29) | .708 | 35 | −0.37 to 0.47 |
| Quality of life | Post-intervention | 25 | 7 | 0.31 (0.17 to 0.46) | <.001 | 61 | −0.23 to 0.86 |
| Quality of life | Short-term | 10 | 1 | 0.50 (0.17 to 0.84) | .003 | 75 | −0.46 to 1.47 |
| Stress | Long-term | 3 | 0 | 0.02 (−0.53 to 0.58) | .931 | 77 | −0.98 to 1.02 |
| Stress | Medium-term | 10 | 0 | 0.30 (0.16 to 0.44) | <.001 | 37 | 0.01 to 0.59 |
| Stress | Post-intervention | 57 | 9 | 0.48 (0.38 to 0.59) | <.001 | 81 | −0.19 to 1.16 |
| Stress | Short-term | 21 | 4 | 0.34 (0.18 to 0.49) | <.001 | 80 | −0.27 to 0.95 |
| Wellbeing | Long-term | 3 | 0 | 0.20 (0.00 to 0.40) | .049 | 0 | 0.00 to 0.40 |
| Wellbeing | Medium-term | 7 | 0 | 0.12 (−0.06 to 0.30) | .203 | 56 | −0.27 to 0.50 |
| Wellbeing | Post-intervention | 38 | 8 | 0.38 (0.24 to 0.53) | <.001 | 85 | −0.39 to 1.16 |
| Wellbeing | Short-term | 12 | 5 | 0.14 (−0.00 to 0.29) | .051 | 63 | −0.23 to 0.52 |

Table B. Specific studies excluded from each sensitivity re-analysis.

| **Outcome** | **Time window** | **Studies excluded (overall high RoB)** |
| --- | --- | --- |
| Anxiety | Post-intervention | Aikman2025; Backman2025; Barcaccia2024; Eustis2018; Lin2017; Listiyandini2025; Lothes2025; Park2025; Pinto2025; Roepke2015; Segal2025; Torok2022; YayPence2025; YsladoMendez2025 |
| Anxiety | Short-term | Eustis2018; Listiyandini2025; Park2025; Roepke2015; Torok2022 |
| Anxiety | Medium-term | Lin2017 |
| Anxiety | Long-term | None |
| Depression | Post-intervention | Aikman2025; Barcaccia2024; Bruhns2021; Buhrman2023; Eustis2018; Lin2017; Listiyandini2025; Lothes2025; Park2025; Pinto2025; Roepke2015; Segal2025; Torok2022; YayPence2025; YsladoMendez2025; Zhu2025 |
| Depression | Short-term | Eustis2018; Listiyandini2025; Park2025; Roepke2015; Torok2022 |
| Depression | Medium-term | Lin2017 |
| Depression | Long-term | None |
| Stress | Post-intervention | Aikman2025; Eustis2018; Harper2025; Listiyandini2025; Lothes2025; Segal2025; Torok2022; YayPence2025; YsladoMendez2025 |
| Stress | Short-term | Eustis2018; Harper2025; Listiyandini2025; Torok2022 |
| Stress | Medium-term | None |
| Stress | Long-term | None |
| Wellbeing | Post-intervention | Adler2025; Aikman2025; Badaghi2025; Krieger2019; Levin2025; Listiyandini2025; Roepke2015; Torok2022 |
| Wellbeing | Short-term | Badaghi2025; Levin2025; Listiyandini2025; Roepke2015; Torok2022 |
| Wellbeing | Medium-term | None |
| Wellbeing | Long-term | None |
| Psychological flexibility | Post-intervention | Bendelin2021; Buhrman2023; Eustis2018; Harper2025; Levin2025; Pinto2025 |
| Psychological flexibility | Short-term | Bendelin2021; Eustis2018; Harper2025; Levin2025 |
| Psychological flexibility | Medium-term | None |
| Psychological flexibility | Long-term | Bendelin2021 |
| Quality of life | Post-intervention | Backman2025; Bruhns2021; Buhrman2023; Eustis2018; Lin2017; Pinto2025; Shahidi2023 |
| Quality of life | Short-term | Eustis2018 |
| Quality of life | Medium-term | Lin2017 |
| Quality of life | Long-term | None |
