# Supplementary material for: Impacts of digital third-wave interventions for adults on wellbeing and mental health outcomes: A systematic review and meta-analysis of randomized controlled trials
Source: PLOS Digit Health. 2026 Oct 1;5(10):e0001764. doi: 10.1371/journal.pdig.0001764 (PMC13630215; doi:10.1371/journal.pdig.0001764)
Supplement: Supplementary file 10 [file pdig.0001764.s010.docx]

**S10 Appendix. Sensitivity analyses using change-score standardised mean differences and standard deviation imputations.**

Text A.

We conducted sensitivity analyses using between-group change-from-baseline scores to assess the robustness of the primary post-intervention findings to the choice of effect metric. For each outcome, post-intervention effects were re-estimated using standardised mean differences (Hedges’ *g*) calculated from the difference in change scores between intervention and control groups.

Change scores were computed as the difference between baseline and post-intervention means within each group. Where the standard deviation of the change score was not reported, it was derived from baseline and post-intervention standard deviations assuming a pre–post correlation of *r* = 0.50, as specified in the protocol. To examine sensitivity to this assumption, additional analyses were conducted using correlations of *r* = 0.30 and *r* = 0.70. All other analytic decisions were held constant, including direction-normalisation (so that positive values indicate improvement), handling of multi-arm trials, and the use of random-effects DerSimonian–Laird models.

Across outcomes, pooled post-intervention effects estimated using change-from-baseline scores were consistent in direction and statistical support with the primary final-value analyses. Effect size magnitudes increased modestly as the assumed pre–post correlation increased, as expected due to the corresponding reduction in the standard deviation of change scores. However, no outcome showed a reversal in effect direction or a substantive change in statistical interpretation across correlation assumptions. Heterogeneity estimates and prediction intervals were broadly comparable to those observed in the primary analyses, indicating persistent between-study variability.

Overall, these findings indicate that the primary post-intervention results are robust to the use of change-from-baseline scores and to reasonable variation in the assumed pre–post correlation, supporting the stability of the main conclusions.

Table A.

| **Outcome** | **r** | **k** | **Hedges’ g** | **95% CI** | **p** | **I² (%)** | **95% Prediction interval** |
| --- | --- | --- | --- | --- | --- | --- | --- |
| Wellbeing | 0.30 | 46 | 0.32 | 0.22 to 0.42 | <.001 | 73.0 | −0.23 to 0.87 |
|  | 0.50 | 46 | 0.38 | 0.27 to 0.48 | <.001 | 77.1 | −0.24 to 0.99 |
|  | 0.70 | 46 | 0.47 | 0.35 to 0.59 | <.001 | 81.9 | −0.24 to 1.19 |
| Depression | 0.30 | 110 | 0.40 | 0.34 to 0.47 | <.001 | 70.3 | −0.14 to 0.95 |
|  | 0.50 | 110 | 0.47 | 0.40 to 0.55 | <.001 | 76.6 | −0.17 to 1.12 |
|  | 0.70 | 110 | 0.60 | 0.51 to 0.68 | <.001 | 83.4 | −0.21 to 1.40 |
| Anxiety | 0.30 | 93 | 0.35 | 0.28 to 0.41 | <.001 | 71.5 | −0.19 to 0.88 |
|  | 0.50 | 93 | 0.40 | 0.33 to 0.48 | <.001 | 77.5 | −0.23 to 1.04 |
|  | 0.70 | 93 | 0.51 | 0.42 to 0.60 | <.001 | 84.2 | −0.28 to 1.31 |
| Stress | 0.30 | 66 | 0.42 | 0.32 to 0.51 | <.001 | 78.3 | −0.22 to 1.05 |
|  | 0.50 | 66 | 0.49 | 0.39 to 0.59 | <.001 | 82.7 | −0.25 to 1.22 |
|  | 0.70 | 66 | 0.62 | 0.50 to 0.74 | <.001 | 87.7 | −0.28 to 1.52 |
| Psychological Flexibility | 0.30 | 51 | 0.34 | 0.23 to 0.46 | <.001 | 74.2 | −0.36 to 1.05 |
|  | 0.50 | 51 | 0.40 | 0.27 to 0.53 | <.001 | 80.7 | −0.45 to 1.25 |
|  | 0.70 | 51 | 0.50 | 0.34 to 0.67 | <.001 | 87.2 | −0.60 to 1.61 |
| Quality of Life | 0.30 | 32 | 0.26 | 0.17 to 0.34 | <.001 | 26.8 | −0.02 to 0.53 |
|  | 0.50 | 32 | 0.31 | 0.20 to 0.41 | <.001 | 46.6 | −0.10 to 0.72 |
|  | 0.70 | 32 | 0.40 | 0.27 to 0.53 | <.001 | 66.4 | −0.21 to 1.02 |
