# Supplementary material for: Impacts of digital third-wave interventions for adults on wellbeing and mental health outcomes: A systematic review and meta-analysis of randomized controlled trials
Source: PLOS Digit Health. 2026 Oct 1;5(10):e0001764. doi: 10.1371/journal.pdig.0001764 (PMC13630215; doi:10.1371/journal.pdig.0001764)
Supplement: Supplementary file 11 [file pdig.0001764.s011.docx]

**S11 Appendix. Sensitivity analyses comparing fixed-effect and random-effects models.**

Text A.

As an additional sensitivity analysis, we examined the robustness of the primary findings to the choice of meta-analytic model by comparing pooled estimates obtained using fixed-effect and random-effects models. For each primary outcome and time window included in the main analyses, pooled effect sizes were extracted from the corresponding fixed-effect and random-effects models estimated using the same study sets and effect size metric (Hedges’ *g*).

Across all outcome–time window combinations, fixed-effect and random-effects models yielded effects in the same direction. All immediate post-intervention estimates remained statistically significant under both models. At long-term follow-up, however, anxiety, depression, and quality of life were statistically significant under fixed-effect models but not under random-effects models. These long-term estimates were based on three to five studies and had substantial heterogeneity. Confidence intervals were generally narrower under fixed-effect models, whereas random-effects models produced wider confidence intervals and prediction intervals where heterogeneity was substantial.

Overall, the direction of effects and the immediate post-intervention conclusions were not sensitive to model choice. The differences for the limited long-term analyses support the use of random-effects models as the primary approach and reinforce the need for caution when interpreting long-term findings.

Table A. Fixed-effect versus random-effects pooled estimates for main analyses

| **Outcome** | **Time window** | **k** | **Fixed-effect g (95% CI)** | **p** | **Random-effects g (95% CI)** | **p** | **I² (%)** | **τ²** | **95% Prediction interval** |
| --- | --- | --- | --- | --- | --- | --- | --- | --- | --- |
| Anxiety | Post-intervention | 93 | 0.35 (0.32 to 0.38) | <.001 | 0.40 (0.32 to 0.48) | <.001 | 78.7 | 0.108 | −0.25 to 1.04 |
|  | Short-term | 36 | 0.31 (0.26 to 0.37) | <.001 | 0.40 (0.27 to 0.53) | <.001 | 79.7 | 0.11 | −0.26 to 1.06 |
|  | Medium-term | 15 | 0.22 (0.14 to 0.30) | <.001 | 0.23 (0.13 to 0.34) | <.001 | 35.1 | 0.014 | −0.02 to 0.49 |
|  | Long-term | 3 | 0.20 (0.03 to 0.38) | .024 | 0.05 (−0.36 to 0.47) | .800 | 76.2 | 0.101 | −0.70 to 0.80 |
| Depression | Post-intervention | 110 | 0.40 (0.36 to 0.43) | <.001 | 0.44 (0.37 to 0.52) | <.001 | 77.8 | 0.111 | −0.21 to 1.10 |
|  | Short-term | 41 | 0.31 (0.26 to 0.36) | <.001 | 0.38 (0.27 to 0.48) | <.001 | 69.0 | 0.066 | −0.14 to 0.89 |
|  | Medium-term | 19 | 0.26 (0.19 to 0.34) | <.001 | 0.30 (0.14 to 0.46) | <.001 | 73.8 | 0.085 | −0.30 to 0.89 |
|  | Long-term | 5 | 0.21 (0.05 to 0.37) | .011 | 0.08 (−0.30 to 0.46) | .670 | 75.3 | 0.134 | −0.73 to 0.90 |
| Stress | Post-intervention | 66 | 0.36 (0.32 to 0.40) | <.001 | 0.46 (0.36 to 0.55) | <.001 | 79.9 | 0.11 | −0.20 to 1.11 |
|  | Short-term | 25 | 0.25 (0.19 to 0.31) | <.001 | 0.35 (0.20 to 0.50) | <.001 | 80.5 | 0.099 | −0.28 to 0.99 |
|  | Medium-term | 10 | 0.25 (0.15 to 0.35) | <.001 | 0.30 (0.16 to 0.44) | <.001 | 37.4 | 0.017 | 0.01 to 0.59 |
|  | Long-term | 3 | 0.16 (−0.05 to 0.37) | .128 | 0.02 (−0.53 to 0.58) | .931 | 76.9 | 0.18 | −0.98 to 1.02 |
| Wellbeing | Post-intervention | 46 | 0.26 (0.22 to 0.31) | <.001 | 0.36 (0.24 to 0.49) | <.001 | 82.3 | 0.125 | −0.34 to 1.07 |
|  | Short-term | 17 | 0.10 (0.04 to 0.16) | .002 | 0.18 (0.05 to 0.31) | .006 | 65.8 | 0.04 | −0.23 to 0.59 |
|  | Medium-term | 7 | 0.08 (−0.03 to 0.18) | .146 | 0.12 (−0.06 to 0.30) | .203 | 56.0 | 0.03 | −0.27 to 0.50 |
|  | Long-term | 3 | 0.20 (0.00 to 0.40) | .049 | 0.20 (0.00 to 0.40) | .049 | 0.0 | 0 | 0.00 to 0.40 |
| Psychological flexibility | Post-intervention | 51 | 0.43 (0.38 to 0.49) | <.001 | 0.41 (0.27 to 0.54) | <.001 | 81.4 | 0.183 | −0.44 to 1.26 |
|  | Short-term | 21 | 0.62 (0.53 to 0.72) | <.001 | 0.54 (0.25 to 0.83) | <.001 | 88.2 | 0.38 | −0.70 to 1.78 |
|  | Medium-term | 9 | 0.06 (−0.06 to 0.19) | .313 | 0.05 (−0.17 to 0.26) | .680 | 62.6 | 0.063 | −0.49 to 0.58 |
|  | Long-term | 4 | 0.04 (−0.16 to 0.23) | .708 | 0.08 (−0.25 to 0.41) | .648 | 55.1 | 0.06 | −0.50 to 0.66 |
| Quality of life | Post-intervention | 32 | 0.28 (0.21 to 0.36) | <.001 | 0.30 (0.19 to 0.42) | <.001 | 57.9 | 0.06 | −0.19 to 0.79 |
|  | Short-term | 11 | 0.50 (0.34 to 0.65) | <.001 | 0.47 (0.15 to 0.78) | .003 | 74.2 | 0.201 | −0.47 to 1.40 |
|  | Medium-term | 7 | 0.09 (−0.06 to 0.23) | .250 | 0.08 (−0.11 to 0.26) | .409 | 27.6 | 0.017 | −0.23 to 0.39 |
|  | Long-term | 3 | 0.22 (0.06 to 0.38) | .007 | 0.09 (−0.30 to 0.48) | .635 | 80.0 | 0.091 | −0.61 to 0.80 |
