# Supplementary material for: Impacts of digital third-wave interventions for adults on wellbeing and mental health outcomes: A systematic review and meta-analysis of randomized controlled trials
Source: PLOS Digit Health. 2026 Oct 1;5(10):e0001764. doi: 10.1371/journal.pdig.0001764 (PMC13630215; doi:10.1371/journal.pdig.0001764)
Supplement: Supplementary file 12 [file pdig.0001764.s012.docx]

**S12 Appendix. Full forest plots and funnel plots for all immediate post-intervention meta-analyses.**

Fig A. Forest plot of post-intervention effects of digital third-wave interventions on wellbeing.

Fig B. Funnel plot of post-intervention wellbeing effect sizes.

Fig C. Forest plot of post-intervention effects of digital third-wave interventions on depressive symptoms.

Fig D. Funnel plot of post-intervention depression effect sizes.

Fig E. Forest plot of post-intervention effects of digital third-wave interventions on anxiety symptoms.

Fig F. Funnel plot of post-intervention anxiety effect sizes.

Fig G. Forest plot of post-intervention effects of digital third-wave interventions on perceived stress.

Fig H. Funnel plot of post-intervention stress effect sizes.

Fig I. Forest plot of post-intervention effects of digital third-wave interventions on psychological flexibility.

Fig J. Funnel plot of post-intervention psychological flexibility effect sizes.

Fig K. Forest plot of post-intervention effects of digital third-wave interventions on quality of life.

Fig L. Funnel plot of post-intervention quality of life effect sizes.
