# Supplementary material for: Impacts of digital third-wave interventions for adults on wellbeing and mental health outcomes: A systematic review and meta-analysis of randomized controlled trials
Source: PLOS Digit Health. 2026 Oct 1;5(10):e0001764. doi: 10.1371/journal.pdig.0001764 (PMC13630215; doi:10.1371/journal.pdig.0001764)
Supplement: Supplementary file 13 [file pdig.0001764.s013.docx]

**S13 Appendix. Influence diagnostics and leave-one-out analyses.**

Text A.

Influence diagnostics and leave-one-out sensitivity analyses were conducted to assess whether the primary findings were disproportionately driven by individual studies. These analyses were restricted to main outcome–timepoint combinations exhibiting substantial between-study heterogeneity (I² ≥ 75%) and including a sufficient number of studies to permit meaningful influence assessment (k ≥ 10).

For each eligible meta-analysis, study influence was evaluated using a leave-one-out approach, whereby the pooled random-effects estimate (Hedges’ g) and heterogeneity statistics were recalculated iteratively following removal of each study in turn. Studies were flagged as potentially influential when their exclusion resulted in a comparatively large absolute change in the pooled effect size and/or a marked reduction in between-study heterogeneity relative to other leave-one-out iterations. On this basis, the single study producing the largest absolute change in the pooled estimate was identified for each analysis.

All studies identified as influential through this procedure were subsequently examined in detail to verify data extraction accuracy, outcome direction, scale handling, and analytic decisions (including multi-arm handling). No errors or inconsistencies were identified. Accordingly, no studies were excluded from the primary analyses, and exclusion of the most influential study was treated as a diagnostic sensitivity analysis to evaluate robustness of the pooled estimates.

Across all analyses examined, exclusion of the most influential individual study resulted in small-to-moderate reductions in pooled effect size magnitude and consistent reductions in between-study heterogeneity (Table A). Importantly, in no case did study exclusion alter the direction of the pooled effect or its statistical interpretation; all pooled effects that were statistically significant in the primary analyses remained statistically significant following exclusion. The largest reductions in heterogeneity were observed in analyses with the highest initial I² values (e.g., Wellbeing and Depression), indicating that certain studies (e.g., Sengupta et al., 2025) contributed disproportionately to between-study variance without driving the overall statistical conclusion.

Table A. Impact of excluding the single most influential study on pooled effect size and heterogeneity

| **Outcome** | **Time window** | **Study excluded** | **Original g (95% CI)** | **Excluded g (95% CI)** | **Δg** | **I² original → excluded** |
| --- | --- | --- | --- | --- | --- | --- |
| Anxiety | Post-intervention | Sengupta2025 | 0.40 (0.32 to 0.48) | 0.37 (0.30 to 0.44) | −0.03 | 79% → 71% |
| Anxiety | Short-term | Wang2024 | 0.40 (0.27 to 0.53) | 0.35 (0.25 to 0.46) | −0.05 | 80% → 63% |
| Depression | Post-intervention | Sengupta2025 | 0.44 (0.37 to 0.52) | 0.42 (0.36 to 0.49) | −0.02 | 78% → 70% |
| Psychological flexibility | Post-intervention | Garcia2024 | 0.41 (0.27 to 0.54) | 0.43 (0.30 to 0.56) | +0.02 | 81% → 80% |
| Psychological flexibility | Short-term | Zhang2024a | 0.54 (0.25 to 0.83) | 0.44 (0.19 to 0.68) | −0.10 | 88% → 84% |
| Stress | Post-intervention | Sengupta2025 | 0.46 (0.36 to 0.55) | 0.42 (0.34 to 0.49) | −0.04 | 80% → 66% |
| Stress | Short-term | Turkoglu2024 | 0.35 (0.20 to 0.50) | 0.31 (0.17 to 0.46) | −0.04 | 80% → 77% |
| Wellbeing | Post-intervention | Sengupta2025 | 0.36 (0.24 to 0.49) | 0.30 (0.22 to 0.39) | −0.06 | 82% → 60% |
